# Enantioselective synthesis of tricyclic amino acid derivatives based on a rigid 4-azatricyclo[5.2.1.0^2,6^]decane skeleton

**DOI:** 10.3762/bjoc.5.81

**Published:** 2009-12-21

**Authors:** Matthias Breuning, Tobias Häuser, Christian Mehler, Christian Däschlein, Carsten Strohmann, Andreas Oechsner, Holger Braunschweig

**Affiliations:** 1 Institut für Organische Chemie, Universität Würzburg, Am Hubland, 97074 Würzburg, Germany; 2 Anorganische Chemie, Universität Dortmund, Otto-Hahn-Str. 6, 44227 Dortmund, Germany; 3 Institut für Anorganische Chemie, Universität Würzburg, Am Hubland, 97074 Würzburg, Germany

**Keywords:** amino acid, enantioselective synthesis, norbornane, polycyclic compounds, pyrrolidine

## Abstract

An enantioselective route to four tricyclic amino acids and *N*-tosylamides, composed of a central norbornane framework with a 2-*endo*,3-*endo*-annelated pyrrolidine ring and a 5-*endo*-C_1_ or -C_2_ side chain, has been developed. A key intermediate was the chiral, *N*-Boc-protected ketone (1*R*,2*S*,6*S*,7*R*)-4-azatricyclo[5.2.1.0^2,6^]decan-8-one, available from inexpensive *endo*-carbic anhydride in five steps and 47% yield. The rigid scaffold makes these amino acid derivatives promising candidates for β-turn-inducing building blocks in peptidomimetics and for chiral auxiliaries in asymmetric organocatalysis.

## Introduction

**Figure 1:**
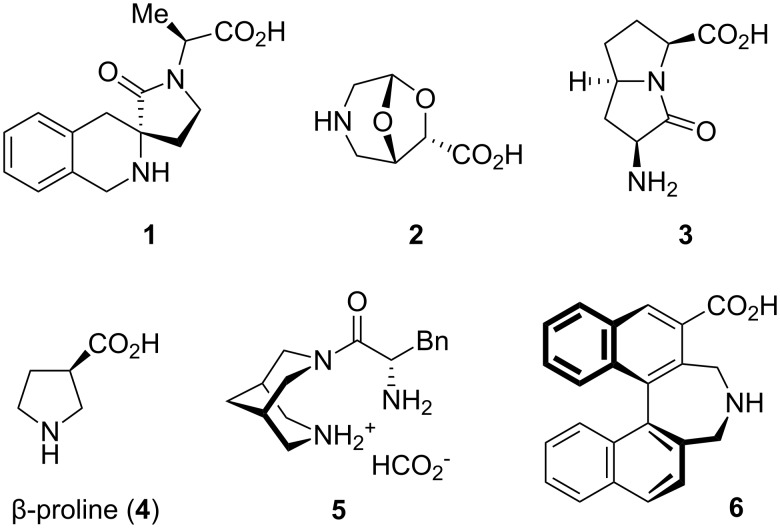
The conformationally rigid amino acid derivatives **1**–**3** (β-turn-inducing building blocks) and **4**–**6** (successful organocatalysts).

Unnatural amino acids with a rigid bowl-shaped backbone have received considerable interest in recent years. Incorporated in peptides or proteins, they may increase the metabolic stability and allow the introduction of novel structural motifs [1–4]. ß-Turns, for example, result if conformationally constrained spiro- or bicyclic amino acids such as **1** [5], **2** [6], and **3** [7–8] are embedded in peptidomimetics (Figure 1). Enantioselective organocatalysis [9–17] is another field of application for conformationally rigid amino acid derivatives. In this context, focus was also put on derivatives in which the activating acidic group is anchored at a more remote position of the molecule, but still in close spatial proximity to the amino function. Examples are β-proline (**4**) [18–19], the bispidinium salt **5** [20], and the binaphthyl-derived amino acid **6** [21–23], which provided excellent enantioselectivities in several aldol and Mannich reactions.

**Figure 2:**
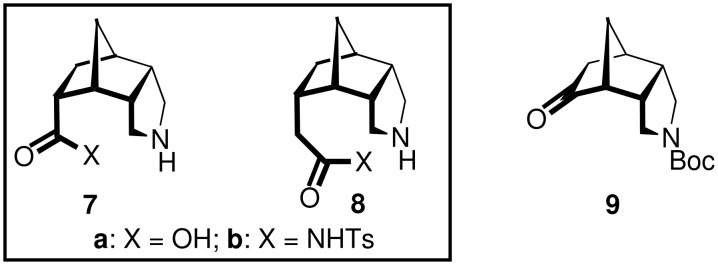
The targeted tricyclic amino acid derivatives **7** and **8**, and the key intermediate **9**.

Our studies targeted the chiral, tricyclic amino acid derivatives **7** and **8** (Figure 2), which possess a central norbornane framework equipped with a 2-*endo*,3-*endo*-annelated pyrrolidine ring. Due to the constrained, bowl-shaped backbone, these compounds may possess high potential as β-turn-inducing peptide building blocks and as bifunctional organocatalysts. In this paper we report on the first enantioselective synthesis of **7** and **8**, which was achieved via the chiral ketone **9** as the key intermediate.

## Results and Discussion

**Scheme 1:**
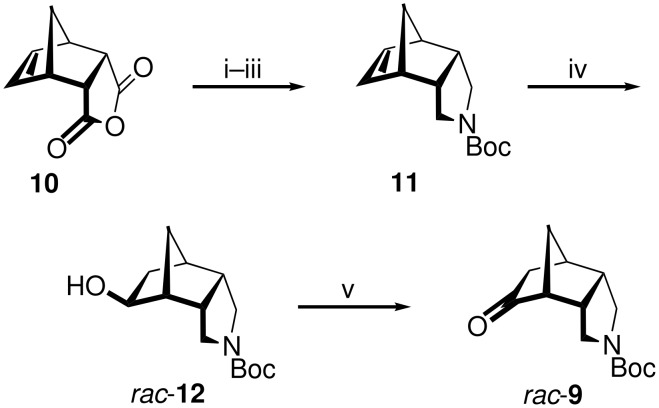
Synthesis of the racemic ketone *rac*-**9**. i) NH_4_OAc, HOAc, Δ, 4 d, 100%; ii) LiAlH_4_, THF, Δ, 1 d, 87% [24]; iii) Boc_2_O, CH_2_Cl_2_, rt, 16 h, 85%; iv) NaBH_4_, Me_2_SO_4_, THF, rt, 6 h, then NaOH, H_2_O_2_, Δ, 90 min, 75%; v) PCC, Celite^®^, CH_2_Cl_2_, rt, 16 h, 79%.

The key intermediate, the tricyclic amino ketone **9**, was first prepared in racemic form starting from inexpensive *endo*-carbic anhydride (**10**, Scheme 1). Conversion of the succinyl anhydride moiety in **10** into the pyrrolidine ring in **11** was accomplished in three steps and 74% yield by imide formation, reduction [24], and N-protection. Hydroboration/oxidation of the alkene function of **11** delivered the *exo*-configured alcohol *rac*-**12**, which was oxidized with PCC furnishing *rac*-**9** in 79% yield.

**Scheme 2:**
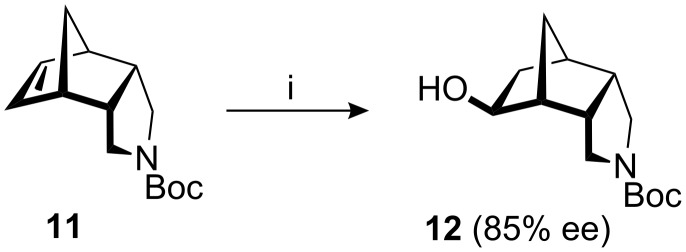
Enantioselective hydrosilylation/oxidation of **11**. i) HSiCl_3_, [Pd(C_3_H_5_)Cl]_2_ (0.06 mol %), (*R*)-MOP (0.25 mol %), toluene, rt, 3 d, then evaporate, then KF, KHCO_3_, H_2_O_2_, THF/MeOH, rt, 1 d, 81%.

**Figure 3:**
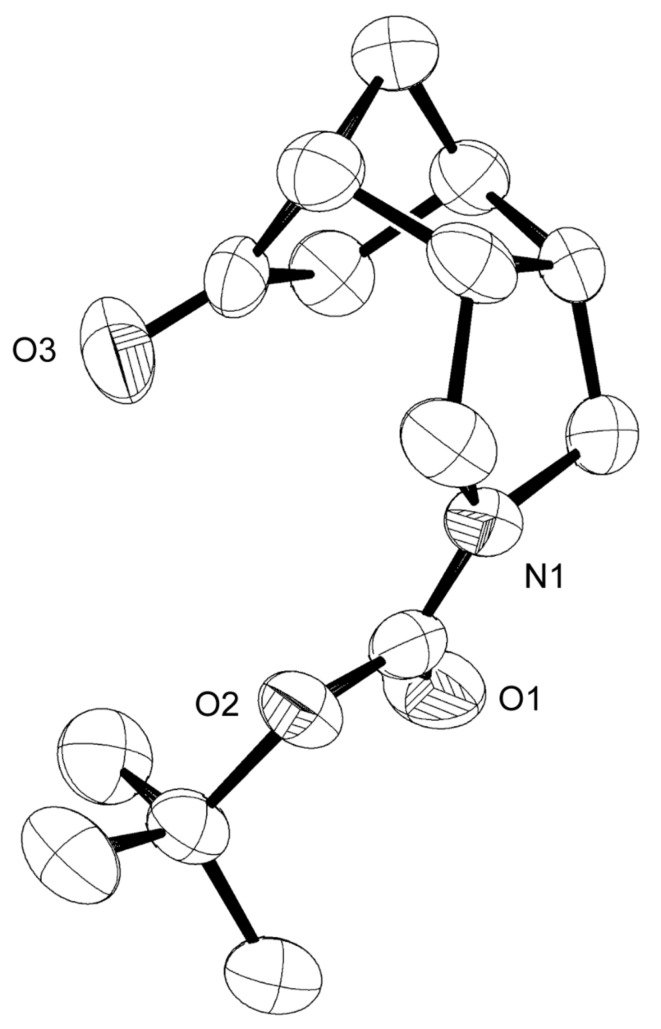
X-ray crystal structure of **9**. X-ray data have been deposited with the Cambridge Crystallographic Data Centre (CCDC 743050).

The asymmetric synthesis of the ketone **9** was realized by enantioselective hydration of the *meso*-alkene **11** using Hayashi’s method (Scheme 2) [25–30]: Hydrosilylation with trichlorosilane in the presence of a catalytic amount of [Pd(C_3_H_5_)Cl]_2_ and (*R*)-MOP [(*R*)-2-diphenylphosphino-2′-methoxy-1,1′-binaphthyl], followed by SiCl_3_/OH exchange, delivered the *exo*-alcohol **12** in 81% yield and 85% ee, as determined from the (*S*)- and (*R*)-Mosher esters of **12**. After oxidation (see Scheme 1), the chiral ketone **9** was thus available in overall five steps and 47% yield from **10**. The X-ray crystal structure of **9** is shown in Figure 3.

**Scheme 3:**
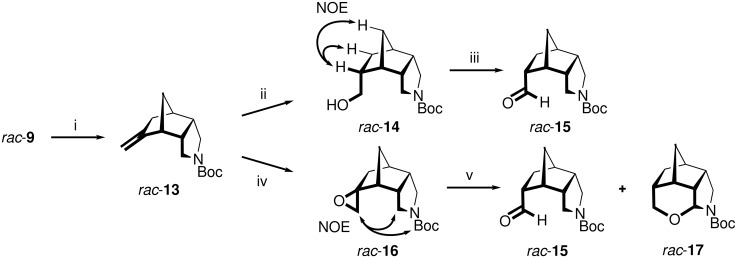
Initial route to the aldehyde *rac*-**15**. i) MePPh_3_^+^Br^−^, *t*-BuOK, toluene, Δ, 7 h, 77% or Mg, TiCl_4_, CH_2_Cl_2_, 0 °C → rt, 2 h, 55%; ii) NaBH_4_, Me_2_SO_4_, THF, 0 °C → rt, 18 h, then NaOH, H_2_O_2_, rt, 3 h, 34%; iii) PCC, CH_2_Cl_2_, rt, 6 h, 51%; iv) MCPBA, CH_2_Cl_2_, rt, 3 h, 60%; v) BF_3_•OEt_2_, toluene, 0 °C, 5 min, 35% (*rac*-**17**) and 26% (*rac*-**15**).

Initial studies on the installation of the functionalized C_1_ side chain, as required for the amino acid derivatives **7**, were done on racemic material and aimed at an oxidation of the alkene *rac*-**13** (Scheme 3), which was available from the ketone *rac*-**9** either by Wittig reaction or by a Tebbe-type olefination [31] using Mg, TiCl_4_, and CH_2_Cl_2_. Hydroboration/oxidation of *rac*-**13** occurred highly diastereoselectively on the *exo*-side providing the desired *endo*-alcohol *rac*-**14**, as determined by NOE measurements. Further oxidation with PCC gave the aldehyde *rac*-**15**, albeit in low 13% overall yield from *rac*-**9**. As an alternative, the epoxidation of *rac*-**13** with MCPBA was investigated, which delivered the spirocyclic *exo*-configured epoxide *rac*-**16** in 46% overall yield from *rac*-**9** as the sole diastereomer. Lewis acid-catalyzed rearrangement of *rac*-**16** with BF_3_ etherate [32] furnished the desired aldehyde *rac*-**15** in 26% yield (12% overall yield from *rac*-**9**) and the tetracyclic *N*,*O*-acetal *rac*-**17** in 35% yield. The latter compound is presumably formed from *rac*-**15** by a Lewis acid-catalyzed, intramolecular, and thus proximity-facilitated tandem hydride transfer/cyclization sequence [33].

**Scheme 4:**
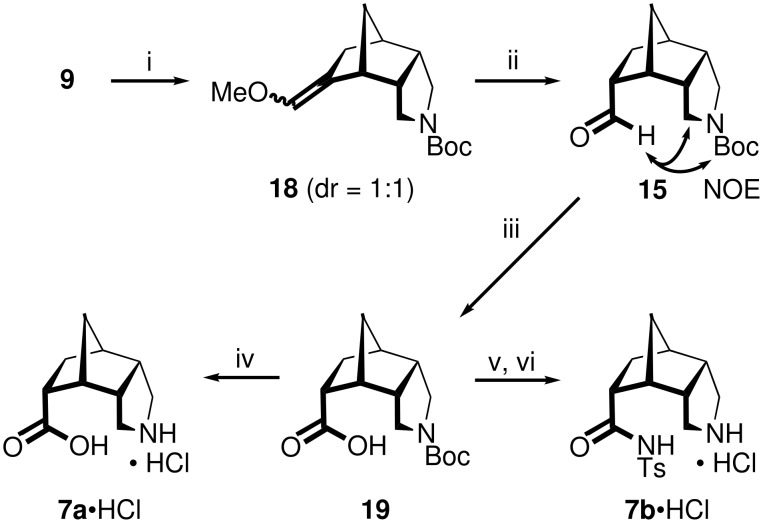
Assembly of the amino acid **7a**•HCl and the *N*-tosylamide **7b**•HCl. i) MeOCH_2_PPh_3_^+^Cl^−^, *t*-BuOK, toluene/THF, rt, 1 d, 84%; ii) Cl_3_CCO_2_H, H_2_O, CH_2_Cl_2_, rt, 1.5 h, 76%; iii) NaClO_2_, H_2_O_2_, KH_2_PO_4_, H_2_O/MeCN, rt, 6 h, 75%; iv) HCl, H_2_O, Δ, 1 d, 79%; v) TsNH_2_, DCC, DMAP, CH_2_Cl_2_, rt, 1 d, 64%; vi) HCl, Et_2_O, MeOH, rt, 3 h, 38%.

Since the yields of *rac*-**15** from the alkene *rac*-**13** were low, we turned our attention to an alternative approach via the enol ether **18**, which was available from **9** as a 1:1 mixture of *E*/*Z*-isomers by Wittig reaction with MeOCH=PPh_3_ (Scheme 4). The selective hydrolysis of the enol ether moiety in **18** in the presence of the *N*-Boc-protective group was achieved by using trichloroacetic acid. The desired *endo*-configured aldehyde **15** was thus available in only two steps in good 64% overall yield from **9**. After oxidation of **15** to the acid **19**, the target amino acid **7a**•HCl was obtained by N-deprotection with aqueous HCl in overall four steps and 38% yield from **9**. The *N*-tosylamide **7b**•HCl was accessed from **19** by condensation with TsNH_2_ under Steglich conditions followed by N-deprotection with ethereal HCl (overall five steps and 12% yield from **9**).

**Scheme 5:**
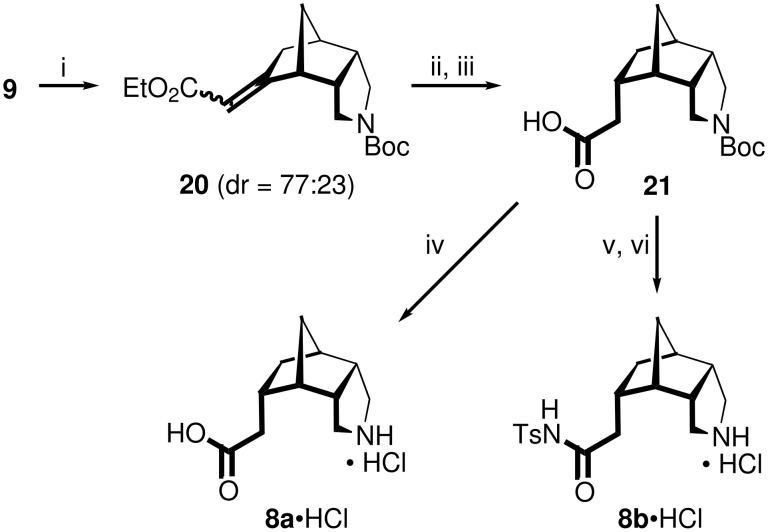
Preparation of the amino acid **8a**•HCl and the *N*-tosylamide **8b**•HCl. i) TMSCH_2_CO_2_Et, LDA, THF, −78 °C → rt, 19 h, 50%; ii) Mg, MeOH, rt, 16 h, 76%; iii) KOH, EtOH/H_2_O, Δ, 1 d, 90%; iv) HCl, H_2_O, Δ, 1 d, 71%; v) TsNH_2_, DCC, DMAP, CH_2_Cl_2_, rt, 4 d, 72%; vi) HCl, Et_2_O, rt, 20 h, 42%.

**Figure 4:**
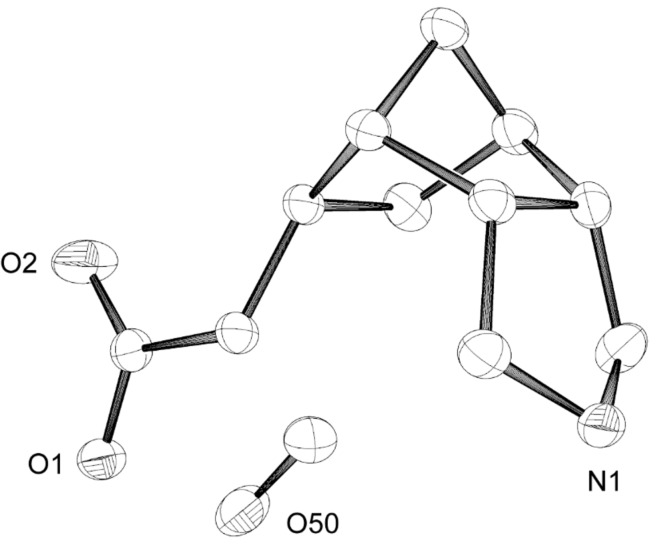
X-ray crystal structure of **8a**•MeOH. X-ray data have been deposited with the Cambridge Crystallographic Data Centre (CCDC 742656).

The preparation of the amino acid **8a**•HCl and the *N*-tosylamide **8b**•HCl required the attachment of an *endo*-oriented acetic acid substituent at the position of the keto group in **9** (Scheme 5). Initial attempts to introduce such a side chain by Wittig or Horner-Wadsworth-Emmons reactions, for example with MeO_2_CCH=PPh_3_ or MeO_2_CCH_2_P(O)(OEt)_2_/*n*-BuLi, failed. By contrast, Peterson-type olefination using TMSCH_2_CO_2_Et/LDA cleanly afforded the α,β-unsaturated ester **20** as a 77:23 mixture of the *E*/*Z*-isomers in 50% yield. The reduction of the conjugated double bond with Mg in methanol furnished, after saponification, the *endo*-configured acid **21** as a single diastereomer. The further conversion of **21** into the target molecules was carried out in analogy to the preparation of **7a**/**b**•HCl from **19** (see Scheme 4), giving **8a**•HCl in overall 24% yield from **9** (four steps) and **8b**•HCl in overall 10% yield (five steps). The required *endo*-orientation of the acetic acid moiety in **8a**•HCl was confirmed by the X-ray structure of the corresponding free base **8a**•MeOH (Figure 4).

A first evaluation of the bowl-shaped amino acid derivatives **7** and **8** in standard organocatalytic aldol and Mannich reactions showed that these compounds are capable of promoting these reactions, albeit with low yields and enantioselectivities. Further investigations on this issue and on the use of **7** and **8** as β-turns are in progress.

## Conclusion

The enantioselective syntheses of the bowl-shaped, tricyclic amino acids and *N*-tosylamides **7** and **8** were successfully accomplished in 9–10 steps starting with inexpensive *endo*-carbic anhydride (**10**). The key stereochemical step was the desymmetrization of the *meso*-alkene **11** using Hayashi’s hydrosilylation/oxidation procedure, which provided the *endo*-alcohol **12** in 85% ee. The target molecules are promising candidates as ß-turn-inducing building blocks in peptidomimetics and as chiral auxiliaries in organocatalysis.

## Supporting Information

File 1 Full experimental details and characterization data for all new compounds.

File 2 NMR spectra of all new compounds.

File 3 Crystallographic data of the compounds **8a**•MeOH and **9**.
