# Supplementary material for: Enantioselective synthesis of tricyclic amino acid derivatives based on a rigid 4-azatricyclo[5.2.1.02,6]decane skeleton
Source: Beilstein J Org Chem. 2009 Dec 21;5:81. doi: 10.3762/bjoc.5.81 (PMC2839531; doi:10.3762/bjoc.5.81)
Supplement: File 1 — Full experimental details and characterization data for all new compounds. [file Beilstein_J_Org_Chem-05-81-s001.pdf]

Supporting Information File 1:

Full experimental details and characterization data for all new compounds

Enantioselective synthesis of tricyclic amino acid derivatives based on a rigid 4-azatricyclo[5.2.1.0^{2,6}]decane skeleton

Matthias Breuning*[,1], Tobias Häuser[1], Christian Mehler[1], Christian Däschlein[2], Carsten Strohmann[2], Andreas Oechsner[3] and Holger Braunschweig[3]

Address: [1]Institut für Organische Chemie, Universität Würzburg, Am Hubland, 97074 Würzburg, Germany, [2]Anorganische Chemie, Universität Dortmund, Otto-Hahn-Str. 6, 44227 Dortmund, Germany and [3]Institut für Anorganische Chemie, Universität Würzburg, Am Hubland, 97074 Würzburg, Germany

Email: Matthias Breuning* - breuning@chemie.uni-wuerzburg.de

* Corresponding author

Table of Contents

1. General Information S3
2. Synthesis of the racemic ketone *rac*-**9** S4
3. Synthesis of the enantiomerically enriched ketone **9** S5
4. Synthesis of the racemic aldehyde *rac*-**15** S7
5. Synthesis of the amino acid **7a**•HCl and of the *N*-tosyl amide **7b**•HCl S10
6. Synthesis of the amino acid **8a**•HCl and of the *N*-tosyl amide **8b**•HCl S13

1. General Information

All reactions were carried out in flame dried flasks under an argon atmosphere with anhydrous solvents. Anhydrous tetrahydrofuran (THF), dichloromethane (CH_2Cl_2), diethyl ether (Et_2O), methanol (MeOH), dimethyl sulfoxide (DMSO), toluene, and acetone were prepared using standard procedures [1].

All reactions were monitored by thin layer chromatography (TLC) on precoated silica gel (Merck F254); spots were visualized by UV light (254 nm) or by staining with aqueous $KMnO_4$. For column chromatography, silica gel (Merck, particle size 63–200 µm) was used.

Carbic anhydride (**10**), (*R*)-MOP [(*R*)-2-diphenylphosphino-2'-methoxy-1,1'-binaphthyl], $[Pd(C_3H_5)Cl]_2$, trichlorosilane ($HSiCl_3$), methyltriphenylphosphonium bromide, pyridinium-chlorochromate (PCC), *meta*-chloroperbenzoic acid (MCPBA), boron trifluoride diethyl etherate (BF_3•OEt_2), (methoxymethyl)triphenylphosphonium chloride, *para*-toluene sulfonamide ($TsNH_2$), *n*-butyllithium (*n*BuLi, 1.6 M in hexanes) and ethyl 2-(trimethylsilyl)acetate are commercially available and were used as received.

Melting point ranges (mp) and decomposition points (dp) were measured on a Reichert Kofler-Heiztisch microscope and are uncorrected. Optical rotations ($[\alpha]_D^T$) were recorded on a Jasco P-1020 polarimeter (10 cm cell). NMR spectra were taken on Bruker Avance 400 and Bruker DMX 600 instruments and calibrated using the residual undeuterated solvent as an internal reference. The peak assignments in the ^1H and ^{13}C NMR data were made on basis of 2D NMR methods (COSY, HSQC, HMBC, NOESY). The following symbols were used for the description of the multiplicities: s = singulett, d = dublett, t = triplett, m = multiplett, quin = quintett, br = broad. Infrared (IR) spectra were recorded on a Jasco FT-IR-3410 spectrometer, high resolution mass spectra (HRMS) on a Bruker Daltonics micrOTOF focus mass spectrometer using ESI (electronspray ionization).

1. Armarego, W. L. F.; Perrin, D. D. *Purification of Laboratory Chemicals*, 4th ed., Butterworth-Heinemann, Oxford, 2000.

2. Synthesis of the racemic ketone *rac*-9

2.1 3,5-Dioxo-*endo*-4-azatricyclo[5.2.1.0^{2,6}]dec-8-ene (I)

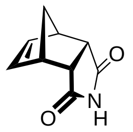

I

A solution of *endo*-carbic anhydride (**10**, 25.2 g, 154 mmol) and NH_4OAc (35.5 g, 461 mmol) in acetic acid (500 mL) was stirred at 140 °C for 4 d. The solvent was evaporated, water (200 mL) was added, and the mixture was extracted with EtOAc (4 × 100 mL). The combined organic layers were dried over $MgSO_4$ and concentrated under reduced pressure. The imide **I** (25.1 g, 154 mmol, 100%) was obtained as a white solid and used in the next step without further purification.

The analytical data of **I** were in accordance with those given in ref. [2].

2.2 *endo*-4-Azatricyclo[5.2.1.0^{2,6}]dec-8-ene (II)

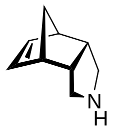

II

$LiAlH_4$ (23.3 g, 614 mmol) was suspended in anhydrous THF (200 mL) and the imide **I** (25.1 g, 154 mmol), dissolved in THF (300 mL), was added dropwise at 0 °C. After 1 d heating at 90 °C, water (60 mL) was added at 0 °C, and the mixture was filtered through a pad of Celite® and washed with EtOAc (500 mL). The solvent was removed in vacuo to deliver the crude amine **II** (18.1 g, 134 mmol, white solid, 87%), which was used in the following step without further purification.

The analytical data of **II** were in accordance with those given in ref. [2].

2.3 4-*tert*-Butoxycarbonyl-*endo*-4-azatricyclo[5.2.1.0^{2,6}]dec-8-ene (11)

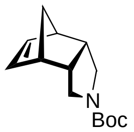

11

A solution of the amine **II** (14.2 g, 105 mmol), Boc_2O (25.1 g, 115 mmol), and DMAP (1.28 mg, 10.5 mmol) in CH_2Cl_2 (500 mL) was stirred for 16 h at rt. Water (200 mL) was added and the mixture was extracted with CH_2Cl_2 (2 × 120 mL). The combined organic layers were washed with brine (200 mL) and dried over $MgSO_4$. After evaporation of the solvent under reduced pressure, the crude product was purified by column chromatography (silica gel, *n*-pentane/Et_2O 1:0 → 4:1) to give **11** as a white solid (21.3 g, 90.5 mmol, 85%). Mp = 35–37 °C; R_f = 0.75 (CH_2Cl_2); ^{1}H NMR (600 MHz, $CDCl_3$, partial signal doubling due to the rotationally hindered *N*-Boc-group): δ = 1.39 (d, 1 H, J = 8.4 Hz, 10-H), 1.40 [s, 9 H, $C(CH_3)_3$], 1.51 (dt, 1 H, J = 8.4, 1.5 Hz, 10-H'), 2.83 (m, 2 H, 2-H, 6-H), 2.86 (br s, 1 H, 1/7-H), 2.88 (br s, 1 H, 1/7-H), 2.97 (dd, 1 H, J = 11.7, 2.1 Hz, 3/5-H), 3.06 (dd, 1 H, J = 11.8, 2.6 Hz, 3/5-H), 3.20 (m, 2 H, 3/5-H'), 6.14 (m, 1 H, 8/9-H), 6.19 (m, 1 H, 8/9-H); ^{13}C NMR (150 MHz, $CDCl_3$, partial signal doubling due to the rotationally hindered *N*-Boc-group): δ = 28.5 [$C(CH_3)_3$], 44.5 (C-2/6), 45.6 (C-2/6), 46.5 (C-1/7), 46.5 (C-1/7), 48.0 (C-3/5), 48.4 (C-3/5), 51.8 (C-10), 78.7 [$C(CH_3)_3$], 134.9 (C-8/9), 135.5 (C-8/9), 153.9 (CO_2N); IR (KBr): $\tilde{\nu}$ = 2967, 2871, 1741, 1697, 1406, 1254, 1176, 1136, 1114, 878, 715, 567 cm^{-1}; HRMS (ESI, pos.): *m/z* calcd for $C_{14}H_{21}NNaO_2$

2. Michaelis, S.; Blechert, S. *Chem.-Eur. J.* **2007**, *13*, 2358–2368.

[M + Na]$^+$: 258.1465; found: 258.1465; Elemental analysis (%) calcd for $C_{14}H_{21}NO_2$ (253.32): C 71.46, H 8.99, N 5.95; found: C 71.18, H 8.98, N, 5.88.

2.4 4-*tert*-Butoxycarbonyl-2-*endo*,6-*endo*,8-*exo*-4-azatricyclo[5.2.1.0^{2,6}]decan-8-ol (*rac*-12)

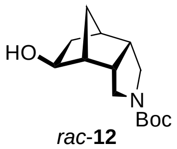

rac-**12**

To a solution of the alkene **11** (4.44 g, 18.9 mmol) in anhydrous THF (80 mL), $NaBH_4$ (927 mg, 24.5 mmol) was added in one portion at 0 °C. Me_2SO_4 (4.05 g, 3.04 mL, 32.1 mmol) was introduced dropwise within 15 min and the reaction mixture was stirred for 6 h at rt. Aqueous H_2O_2 (35%, 44 mL), 1 N NaOH (22 mL), and water (33 mL) were added at 0 °C and the mixture was heated to reflux for 90 min. THF was evaporated and the remaining aqueous phase was extracted at 0 °C with CH_2Cl_2 (4 × 50 mL). The combined organic layers were washed with brine (50 mL), dried over $MgSO_4$, and concentrated under reduced pressure. Purification of the crude material by column chromatography (silica gel, *n*-pentane/Et_2O 4:3 → 2:3) gave the racemic alcohol *rac*-**12** (3.59 g, 14.2 mmol, 75%) as a colorless oil.

The spectroscopic data of *rac*-**12** were identical to those of **12** given in section 3.1.

2.5 4-*tert*-Butoxycarbonyl-*endo*-4-azatricyclo[5.2.1.0^{2,6}]decan-8-one (*rac*-9)

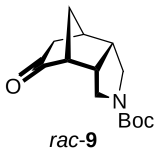

rac-**9**

PCC (6.08 g, 28.2 mmol) and Celite® (26.0 g) were suspended in CH_2Cl_2 (130 mL). A solution of *rac*-**12** (3.57 g, 14.1 mmol) in CH_2Cl_2 (130 mL) was added dropwise. The mixture was stirred overnight and then filtered through a pad of Celite®. The filter cake was washed with EtOAc (300 mL) and the combined organic layers were dried over $MgSO_4$ and evaporated. The residue was purified by column chromatography (silica gel, *n*-pentane/Et_2O 1:0 → 0:1) to give the ketone *rac*-**9** (2.78 g, 11.1 mmol, 79%) as a white solid.

The spectroscopic data of *rac*-**9** were identical to those of **9** given in section 3.3.

3. Synthesis of the enantiomerically enriched ketone 9

3.1 (1*R*,2*S*,6*S*,7*R*,8*S*)-4-*tert*-Butoxycarbonyl-4-azatricyclo[5.2.1.0^{2,6}]decan-8-ol (12)

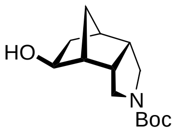

12

The alkene **11** (2.39 g, 10.2 mmol) was dissolved in anhydrous toluene (4.8 mL) under an argon atmosphere and cooled to 0 °C. (*R*)-MOP (12.0 mg, 25.6 µmol), $[Pd(C_3H_5)Cl]_2$ (2.30 mg, 6.29 µmol), and trichlorosilane (4.43 g, 3.31 mL, 32.7 mmol) were added consecutively. The reaction was warmed to rt and stirred for 3 d. After evaporation of the solvent, the residue was re-dissolved in THF (22 mL) and MeOH (22 mL) and poured at 0 °C into a suspension of KF (4.74 g, 81.6 mmol) and $KHCO_3$ (10.2 g, 102 mmol) in THF (22 mL) and MeOH (22 mL). Aqueous H_2O_2 (30%, 12.3 mL) was added and the reaction mixture was stirred for 1 d at rt. The suspension was filtered and the filter cake was washed with MeOH (2 × 50 mL). The filtrate was concentrated under reduced pressure and water (50 mL) and Et_2O (50 mL) were added. The aqueous layer was extracted with Et_2O (3 × 50 mL), and the combined organic layers were dried over $MgSO_4$ and evaporated under reduced pressure. The crude

product was purified by column chromatography (silica gel, *n*-pentane/Et_2O 4:3 → 3:4) to give **12** (2.08 g, 8.21 mmol, 81%) as a colorless oil. $[\alpha]_D^{22}$ = 12.8 (*c* = 0.46, CH_2Cl_2); R_f = 0.25 (CH_2Cl_2/MeOH 95:5); ^{1}H NMR (600 MHz, $CDCl_3$, 1:1 mixture of rotamers): δ = 1.18 (t, 1 H, *J* = 10.0 Hz, 9-H), 1.37 (m, 1 H, 10-H), 1.45 [s, 9 H, $C(CH_3)_3$], 1.79 (t, 1 H, *J* = 8.7 Hz, 10-H'), 1.94 (dd, 0.5 H, *J* = 12.4, 6.5 Hz, 9-H'), 2.03 (dd, 0.5 H, *J* = 13.2, 6.0 Hz, 9-H'), 2.25 (m, 2 H, 1-H, 7-H), 2.42 (m, 1 H, 2-H), 2.54 (s, 1 H, 6-H), 2.99 (m, 2 H, 3-H, 5-H), 3.44 (d, 0.5 H, *J* = 12.1 Hz, 3-H'), 3.52 (d, 0.5 H, *J* = 11.9 Hz, 3-H'), 3.59 (d, 0.5 H, *J* = 12.1 Hz, 5-H'), 3.69 (d, 0.5 H, *J* = 12.0 Hz, 5-H'), 3.90 (br s, 0.5 H, 8-H), 3.92 (br s, 0.5 H, 8-H); ^{13}C NMR (150 MHz, $CDCl_3$, 1:1 mixture of rotamers): δ = 28.5 [$C(CH_3)_3$], 35.6 (C-9), 36.0 (C-9), 38.3 (C-10), 40.5 (C-1), 41.2 (C-2), 41.6 (C-6), 42.2 (C-2), 42.6 (C-6), 45.2 (C-5), 45.7 (C-5), 46.1 (C-3), 46.5 (C-3), 49.4 (C-7), 69.1 (C-8), 69.4 (C-8), 79.3 [*C*(CH_3)], 154.0 (CO_2N); IR (film): $\tilde{\nu}$ = 3426, 2957, 2872, 1674, 1420, 1240, 1172, 1116, 874, 454 cm^{-1}; HRMS (ESI, pos.): *m/z* calcd for $C_{14}H_{23}NNaO_3$ [M + Na]$^+$: 276.1570; found: 276.1572.

3.2 General procedure GP1 (synthesis of the Mosher esters of 12)

(*R*)-(–)- or (*S*)-(+)-α-Methoxy-α-trifluoromethyl phenylacetic acid chloride (2.00 equiv) were added at rt to a solution of **12**, NEt_3 (2.70 equiv), and a catalytic amount of DMAP in anhydrous CH_2Cl_2 (37 mL/mmol **12**). After 18 h, water (187 mL/mmol **12**) was added and the solution was extracted with Et_2O (3 × 375 mL/mmol **12**). The combined organic layers were dried over $MgSO_4$ and evaporated under reduced pressure. Purification of the crude product by column chromatography (silica gel, *n*-pentane/Et_2O 1:0 → 4:1) delivered the Mosher esters (*S*)-**III** or (*R*)-**III** as colorless liquids.

3.2.1 (*S*)-Mosher ester of 12

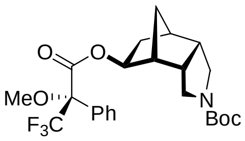

(*S*)-**III**

The (*S*)-Mosher ester (*S*)-**III** (20.0 mg, 42.5 µmol, 63%, colorless liquid) was obtained in 85% de (according to line shape analysis) from **12** (17.0 mg, 67.1 µmol) and the (*R*)-Mosher acid chloride (34.0 mg, 25.1 µL, 134 µmol) according to GP1. ^{1}H NMR (400 MHz, $CDCl_3$): δ = 1.35 (m, 1 H), 1.43 (m, 1 H), 1.49 [s, 9 H, $C(CH_3)_3$], 1.57 (br s, 1 H), 1.68 (br d, 1 H, *J* = 10.2 Hz), 2.08 (br s, 1H), 2.47 (m, 2 H), 2.61 (m, 1 H), 3.03 (m, 2 H), 3.42–2.62 (m, 4 H), 3.78 (d, 1 H, *J* = 12.2 Hz), 7.39 (m, 3 H, Ph), 7.51 (m, 2 H, Ph).

3.2.2 (*R*)-Mosher ester of 12

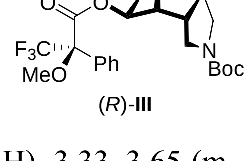

(*R*)-**III**

The (*S*)-Mosher acid chloride (34.0 mg, 25.1 µL, 134 µmol) was treated with **12** (17.0 mg, 67.1 µmol) following GP1 to give (*R*)-**III** (20.3 mg, 43.1 µmol, 64%) as a colorless liquid in 85% de (according to line shape analysis). ^{1}H NMR (400 MHz, $CDCl_3$): δ = 1.42 (m, 2 H), 1.48 [s, 9 H, $C(CH_3)_3$], 1.59 (m, 2 H), 2.10 (m, 1 H), 2.30 (br s, 1 H), 2.45 (m, 2 H), 2.60 (m, 1 H), 3.04 (m, 2 H), 3.33–3.65 (m, 4 H), 3.78 (d, 1 H, *J* = 12.2 Hz), 7.39 (m, 3 H, Ph), 7.51 (m, 2 H, Ph).

3.3 (1*R*,2*S*,6*S*,7*R*)-4-*tert*-Butoxycarbonyl-4-azatricyclo[5.2.1.0^{2,6}]decan-8-one (9)

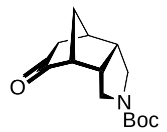

9

The ketone **9** (849 mg, 3.38 mmol, 86%) was synthesized from **12** (1.00 g, 3.95 mmol), according to the procedure described for *rac*-**9** in section 2.5. Mp = 111–113 °C; $[\alpha]_D^{22}$ = 99.9 (c = 1.08, CH_2Cl_2); R_f = 0.45 (CH_2Cl_2/MeOH 95:5); ^{1}H NMR (600 MHz, $CDCl_3$): δ = 1.43 [s, 9 H, $C(CH_3)_3$], 1.74 (dd, 1 H, J = 10.3, 3.7 Hz, 10-H), 1.82 (d, 1 H, J = 10.4 Hz, 10-H'), 1.96 (m, 1 H, 9-H), 2.08 (m, 1 H, 9-H'), 2.58 (m, 1 H, 7-H), 2.68 (br s, 1 H, 1-H), 2.76 (m, 1 H, 2-H), 2.85 (m, 1 H, 6-H), 3.09 (m, 2 H, 3-H, 5-H), 3.44 (m, 1 H, 5-H'), 3.61 (d, 1 H, J = 12.0 Hz, 3-H'); ^{13}C NMR (150 MHz, $CDCl_3$, mixture of rotamers): δ = 28.4 [$C(CH_3)_3$], 39.0 (C-1), 39.5 (C-9), 39.7 (C-10), 41.2 (C-2), 42.1 (C-2), 43.8 (C-6), 44.9 (C-6), 46.0 (C-3), 46.2 (C-3), 46.5 (C-5), 46.6 (C-5), 55.5 (C-7), 55.7 (C-7), 79.5 [$C(CH_3)_3$], 79.8 [$C(CH_3)_3$], 153.6 (CO_2N), 154.0 (CO_2N), 214.2 (C-8), 214.8 (C-8); IR (KBr): $\tilde{\nu}$ = 3464, 2967, 2932, 2888, 1741, 1686, 1422, 1166, 1123, 457 cm^{-1}; HRMS (ESI, pos.): m/z calcd for $C_{14}H_{21}NNaO_3$ [M + Na]$^+$: 274.1414; found: 274.1414; Elemental analysis (%) calcd for $C_{14}H_{21}NO_3$ (251.32): C 66.91, H 8.42, N 5.57; found: C 67.03, H 8.18, N 5.47.

4. Synthesis of the racemic aldehyde *rac*-15

4.1 4-*tert*-Butoxycarbonyl-8-methylidene-*endo*-4-azatricyclo[5.2.1.0^{2,6}]decane (*rac*-13)

4.1.1 Methylenation of *rac*-9 by Wittig reaction

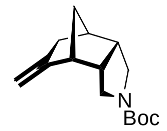

rac-**13**

A suspension of KO*t*Bu (148 mg, 1.29 mmol) and methyltriphenylphosphonium bromide (462 mg, 1.29 mmol) in anhydrous toluene (2 mL) was heated to reflux for 2 h. The racemic ketone *rac*-**9** (250 mg, 995 µmol) was added and heating was continued for 5 h. Water (5 mL) was added and the mixture was extracted with Et_2O (3 × 10 mL). The combined organic layers were washed with brine (10 mL), dried over $MgSO_4$, and evaporated under reduced pressure. The crude material was purified by column chromatography (silica gel, *n*-pentane/Et_2O 1:0 → 4:1) to deliver the alkene *rac*-**13** (190 mg, 762 µmol, 77%) as a colorless oil. R_f = 0.24 (*n*-pentane/Et_2O 4:1); ^{1}H NMR (600 MHz, $CDCl_3$, 1:1 mixture of rotamers): δ = 1.43 [m, 9 H, $C(CH_3)_3$], 1.54 (br d, 1 H, J = 9.5 Hz, 10-H), 1.60 (br d, 1 H, J = 9.3 Hz, 10-H'), 1.99 (m, 1 H, 9-H), 2.10 (m, 1 H, 9-H'), 2.33 (br s, 1 H, 1-H), 2.51–2.63 (m, 3 H, 2-H, 6-H, 7-H), 3.02 (m, 2 H, 3-H, 5-H), 3.47 (m, 1.5 H, 3-H', 5-H'), 3.58 (br d, 0.5 H, J = 12.0 Hz, 3-H', 5-H'), 4.69 (s, 1 H, C=C*H*H), 4.82 (br s, 1 H, C=CH*H*); ^{13}C NMR (150 MHz, $CDCl_3$, 1:1 mixture of rotamers): δ = 28.5 [$C(CH_3)_3$], 31.5 (C-9), 40.8 (C-1), 41.9 (C-10), 42.2 (C-2 or C-6), 43.1 (C-2 or C-6), 44.1 (C-2 or C-6), 45.0 (C-2 or C-6), 45.8 (C-3 or C-5), 46.2 (C-3 or C-5), 46.3 (C-3 or C-5), 46.6 (C-3 or C-5), 50.7 (C-7), 50.8 (C-7), 78.8 [$C(CH_3)_3$], 105.0 (C=CH_2), 105.6 (C=CH_2), 148.6 (C-8), 149.6 (C-8), 153.6 (CO_2N), 153.9 (CO_2N); IR (KBr): $\tilde{\nu}$ = 3069, 2955, 2870, 1697, 1416, 1391, 1364, 1240, 1173, 1111, 877 cm^{-1}; HRMS (ESI, pos.): m/z calcd for $C_{15}H_{23}NNaO_2$ [M + Na]$^+$: 272.1621; found: 272.1623.

4.1.2 Methylenation of *rac*-9 with CH_2Cl_2 promoted by Mg/$TiCl_4$

A solution of the ketone *rac*-**9** (400 mg, 1.59 mmol) in anhydrous THF (3.1 mL) was added dropwise at 0 °C to a suspension of Mg (309 mg, 12.7 mmol) and $TiCl_4$ (604 mg, 339 µL, 3.18 mmol) in anhydrous CH_2Cl_2 (6.3 mL). The reaction mixture was stirred for 1 h at 0 °C and for 1 h at rt. The suspension was cooled to 0 °C, treated with saturated aqueous K_2CO_3 (20 mL), filtered through a pad of Celite®, and washed with CH_2Cl_2 (100 mL). Saturated aqueous K_2CO_3 (20 mL) was added and the organic layer was extracted with CH_2Cl_2 (2 × 100 mL). The organic layers were combined, washed with brine (100 mL), dried over $MgSO_4$, and concentrated under reduced pressure. The product *rac*-**13** (220 mg, 882 µmol, 55%) was obtained by column chromatography (silica gel, *n*-pentane/Et_2O 1:0 → 5:1).

For the spectroscopic data of *rac*-**13**, see the preceding procedure.

4.2 4-*tert*-Butoxycarbonyl-*endo*-8-(hydroxymethyl)-*endo*-4-azatricyclo[5.2.1.0^{2,6}]decane (*rac*-14)

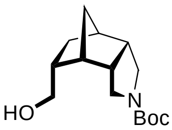

rac-**14**

$NaBH_4$ (37.2 mg, 984 µmol) and Me_2SO_4 (163 mg, 123 µL, 1.29 mmol) were added to a solution of *rac*-**13** (189 mg, 758 µmol) in anhydrous THF (6 mL) at 0 °C. After 18 h at rt, the reaction mixture was cooled to 0 °C and water (1.35 mL), NaOH (1 N, 900 µL), and aqueous H_2O_2 (30%, 1.74 mL) were added. The reaction mixture was stirred for 3 h at rt. Water (20 mL) was added and the mixture was extracted with Et_2O (3 × 60 mL). The combined organic layers were washed with brine (20 mL), dried over $MgSO_4$, and evaporated. Purification by column chromatography (silica gel, *n*-pentane/Et_2O 1:0 → 0:1) gave the racemic alcohol *rac*-**14** (68.4 mg, 256 µmol, 34%) as a colorless oil. R_f = 0.10 (*n*-pentane/Et_2O 1:1); ^{1}H NMR (600 MHz, $CDCl_3$): δ = 1.10 (m, 1 H, 9-H), 1.46 [s, 9 H, $C(CH_3)_3$], 1.50 (m, 1 H, 10-H), 1.54 (d, 1 H, J = 9.5 Hz, 10-H'), 1.67 (m, 1 H, 9-H'), 1.75 (br s, 1 H, OH), 2.16 (br s, 1 H, 8-H), 2.28 (br s, 1 H, 1-H), 2.40 (br d, 1 H, J = 9.3 Hz, 7-H), 2.59 (m, 2 H, 2-H, 6-H), 3.05 (dd, 1 H, J = 12.4, 8.6 Hz, 3-H or 5-H), 3.12 (m, 1 H, 3-H or 5-H), 3.46–3.61 (m, 2 H, 3-H', 5-H'), 3.68 (m, 2 H, CH_2OH); ^{13}C NMR (150 MHz, $CDCl_3$, mixture of rotamers): δ = 25.1 (C-9), 25.3 (C-9), 28.5 [$C(CH_3)_3$], 41.3 (C-1), 43.0 (C-2 or C-6), 43.06 (C-2 or C-6), 43.15 (C-7), 44.0 (C-10), 44.1 (C-2 or C-6), 44.2 (C-8), 44.8 (C-8), 45.7 (C-3 or C-5), 46.0 (C-3 or C-5), 46.5 (C-3 or C-5), 47.0 (C-3 or C-5), 64.2 (CH_2OH), 64.4 (CH_2OH), 79.3 [$C(CH_3)_3$], 154.39 (CO_2N), 154.45 (CO_2N); IR (KBr): $\tilde{\nu}$ = 3417, 2945, 2875, 1691, 1674, 1394, 1365, 1169, 1138, 1105, 1012, 874, 777 cm^{-1}; HRMS (ESI, pos.): *m/z* calcd for $C_{15}H_{25}NNaO_3$ [M + Na]$^+$: 290.1727; found: 290.1727.

4.3 4-*tert*-Butoxycarbonyl-spiro[*endo*-4-azatricyclo[5.2.1.0^{2,6}]decane-8,1'-*exo*-2'-oxacyclo-propane] (*rac*-16)

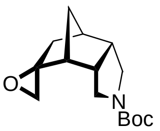

rac-**16**

MCPBA (70%, 212 mg, 860 µmol) and $NaHCO_3$ (515 mg, 6.14 mmol) were added at 0 °C to a solution of the alkene *rac*-**13** (153 mg, 610 µmol) in CH_2Cl_2 (7 mL). The reaction mixture was stirred for 3 h at rt. Excess MCPBA was decomposed by treatment with aqueous Na_2SO_3 (0.5 M, 10 mL). After extraction of the crude reaction mixture with CH_2Cl_2 (3 × 10 mL), the combined organic layers were washed with saturated aqueous $NaHCO_3$ (10 mL), dried with $MgSO_4$, and evaporated. The crude product was purified by column chromatography (silica gel, *n*-pentane/Et_2O 1:0 → 2:1) to afford *rac*-**16** (97.7 mg, 368 µmol, 60%) as a white powder. Mp = 60–62 °C; R_f = 0.33 (*n*-pentane/Et_2O 1:1); ^{1}H NMR (600 MHz, $CDCl_3$, 1:1 mixture of rotamers): δ = 1.45 [s, 9 H, $C(CH_3)_3$], 1.52 (quin, 0.5 H, J = 1.4 Hz, 10-H), 1.53 (quin, 0.5 H, J = 1.4 Hz, 10-H), 1.55 (m, 0.5 H, 9-H), 1.57 (m, 0.5 H, 9-H), 1.76 (br d, 0.5 H, J = 14.3 Hz, 9-H'), 1.82 (br d, 0.5 H, J = 14.0 Hz, 9-H'), 1.85 (m, 1 H, 7-H), 1.89 (t, 0.5 H, J = 1.5 Hz, 10-H'), 1.90 (t, 0.5 H, J = 1.5 Hz, 10-H'), 2.42 (br s, 1 H, 1-H), 2.54 (m, 1 H, 2-H), 2.62 (m, 1 H, 6-H), 2.83 (d, 0.5 H, J = 3.4 Hz, C*H*HO), 2.86 (d, 0.5 H, J = 3.9 Hz, C*H*HO), 2.95 (d, 0.5 H, J = 3.9 Hz, CH*H*O), 3.02 (m, 2.5 H, 3-H, 5-H, CH*H*O), 3.55 (d, 0.5 H, J = 12.2 Hz, 3-H'), 3.61 (d, 0.5 H, J = 11.9 Hz, 3-H'), 3.65 (d, 0.5 H, J = 12.0 Hz, 5-H'), 3.78 (d, 0.5 H, J = 12.1 Hz, 5-H'); ^{13}C NMR (150 MHz, $CDCl_3$, 1:1 mixture of rotamers): δ = 28.50 [$C(CH_3)_3$], 28.54 [$C(CH_3)_3$], 32.08 (C-9), 32.14 (C-9), 40.9 (C-10), 41.1 (C-1), 41.2 (C-1), 41.5 (C-2), 42.4 (C-6), 42.6 (C-2), 43.3 (C-6), 45.3 (C-5), 45.9 (C-5), 46.0 (C-3), 46.5 (C-3), 47.9 (C-7), 51.2 (CH_2O), 51.5 (CH_2O), 63.1 (C-8), 63.3 (C-8), 79.2 [$C(CH_3)_3$], 79.3 [$C(CH_3)_3$], 153.6 (CO_2N), 153.7 (CO_2N); IR (ATR): $\tilde{\nu}$ = 2956, 2868, 1686, 1481, 1426, 1364, 1242, 1164, 1133, 874, 762 cm^{-1}; HRMS (ESI, pos.): *m/z* calcd for $C_{15}H_{24}NO_3$ [M + H]$^+$: 266.1751; found: 266.1751.

4.4 4-*tert*-Butoxycarbonyl-2-*endo*,6-*endo*,8-*endo*-4-azatricyclo[5.2.1.0^{2,6}]decane-8-carbalde-hyde (*rac*-15)

4.4.1 Oxidation of the alcohol *rac*-14

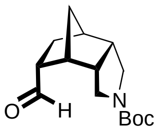

rac-**15**

A solution of *rac*-**14** (35.4 mg, 132 µmol) in CH_2Cl_2 (1.50 mL) was added dropwise at rt to a suspension of PCC (57.0 mg, 264 µmol) and Celite® (251 mg) in CH_2Cl_2 (1.50 mL). The mixture was stirred for 6 h at rt, filtered through a pad of Celite®, and washed with EtOAc (150 mL). The organic layer was dried over $MgSO_4$ and evaporated. The residue was purified by column chromatography (silica gel, *n*-pentane/EtOAc 1:0 → 2:1) to give the aldehyde *rac*-**15** (18.0 mg, 67.8 µmol, 51%) as a colorless oil.

The spectroscopic data of *rac*-**15** were identical to those of **15** given in section 5.2.

4.4.2 4-*tert*-Butoxycarbonyl-2-*endo*,8-*endo*,12-*endo*-4-aza-6-oxatetracyclo[6,2,1,$1^{2,5}$,$0^{9,12}$]-undecane (*rac*-17) and *rac*-15 via Lewis acid-catalyzed rearrangement of *rac*-16

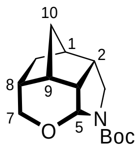

rac-**17**

The epoxide *rac*-**16** (31.2 mg, 118 µmol) was dissolved in anhydrous toluene (11 mL) and BF_3•OEt_2 (4.14 mg, 3.70 µL, 29.3 µmol) was added at 0 °C. After stirring at 0 °C for 5 min, water (10 mL) was added and the mixture was extracted with Et_2O (3 × 10 mL). The combined organic layers were dried over K_2CO_3 and evaporated under reduced pressure. Column chromatographic separation (silica gel, *n*-pentane/Et_2O 1:0 → 2:1) delivered *rac*-**17** (11.0 mg, 41.5 µmol, 35%) as a white solid and *rac*-**15** (8.00 mg, 30.1 µmol, 26%) as a colorless oil. *Rac*-**17**: Mp = 65–67 °C; R_f = 0.40 (*n*-pentane/Et_2O 1:1); ^{1}H NMR (600 MHz, $CDCl_3$, 1:1 mixture of rotamers): δ = 1.45 [s, 9 H, $C(CH_3)_3$], 1.56 (m, 1 H, 11-H), 1.62 (s, 2 H, 10-H, 10-H'), 1.71 (br dd, 1 H, J = 13.0, 4.0 Hz, 11-H'), 1.92 (m, 1 H, 8-H), 2.14 (br s, 1 H, 1-H), 2.19 (br s, 1 H, 9-H), 2.43 (br s, 1 H, 12-H), 2.62 (br s, 1 H, 2-H), 3.30 (dd, 1 H, J = 11.3, 6.2 Hz, 3-H), 3.38–3.60 (m, 2 H, 3-H', 7-H), 3.66 (br d, 1 H, J = 11.2 Hz, 7-H'), 5.20 (br s, 0.5 H, 5-H), 5.33 (br s, 0.5 H, 5-H); ^{13}C NMR (150 MHz, $CDCl_3$, 1:1 mixture of rotamers): δ = 25.6 (C-11), 28.4 [$C(\mathit{C}H_3)_3$], 33.6 (C-9), 37.9 (C-9), 40.1 (C-1), 41.9 (C-12), 43.0 (C-12), 43.5 (C-3), 44.1 (C-3), 44.6 (C-10), 45.1 (C-2), 63.4 (C-7), 79.7 [$\mathit{C}(CH_3)_3$], 80.7 (C-5), 80.9 (C-5), 154.1 (CO_2N), 154.5 (CO_2N); IR (ATR): $\tilde{\nu}$ = 2947, 1697, 1389, 1364, 1344, 1330, 1169, 1151, 1103, 083, 1008, 893 cm^{-1}; HRMS (ESI, pos.): *m/z* calcd for $C_{15}H_{23}NNaO_3$ [M + Na]$^+$: 288.1570; found: 288.1563.

The spectroscopic data of *rac*-**15** were identical to those of **15** given in section 5.2.

5. Synthesis of the amino acid 7a•HCl and of the *N*-tosyl amide 7b•HCl

5.1 (1*R*,2*S*,6*R*,7*R*)-4-*tert*-Butoxycarbonyl-8-methoxymethylidene-4-azatricyclo[5.2.1.$0^{2,6}$]-decane (18)

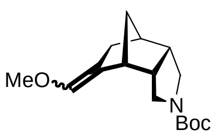

18 (dr = 1:1)

A suspension of (methoxymethyl)triphenylphosphonium chloride (15.9 g, 46.4 mmol) and KO*t*Bu (6.25 g, 55.7 mmol) in anhydrous toluene (375 mL) was stirred at rt for 5 h. A solution of the ketone **9** (1.61 g, 6.41 mmol) in anhydrous THF (90 mL) was added slowly via a syringe and stirring was continued for 1 d at rt. EtOAc (400 mL) was added and the mixture was washed with water (3 × 100 mL) and brine (2 × 100 mL). The organic layer was dried over K_2CO_3 and concentrated under reduced pressure. Purification by column chromatography (silica gel, *n*-pentane/Et_2O 10:1) gave **18** (1.51 g, 5.41 mmol, 84%, 1:1 mixture of *E*/*Z*-isomers) as a colorless oil. $[\alpha]_D^{20}$ = 23.5 (c = 0.56, $CHCl_3$); R_f = 0.26 (*n*-pentane/Et_2O 4:1); ^{1}H NMR (600 MHz, $CDCl_3$, 1:1:1:1 mixture of rotamers and *E*/*Z*-isomers): δ = 1.45 [m, 9 H, $C(CH_3)_3$], 1.53 (m, 1.5 H, 10-H), 1.58 (m, 0.5 H, 10-H), 1.99 (m, 1.5 H, 9-H), 1.99 (m, 0.5 H, 9-H), 2.30 (m, 0.5 H, 1-H), 2.30 (m, 0.5 H, 1-H), 2.47 (m, 0.5 H, 7-H), 2.50–2.67 (m, 2 H, 2-H, 6-H), 2.97–3.10 (m, 2.5 H, 3-H, 5-H, 7-H), 3.35 (m, 0.5, 3-H, 5-H), 3.47 (m, 1 H, 3-H, 5-H), 3.54 (m, 3.5 H, 3-H, 5-H, OCH_3), 5.78 (br s, 0.2 H, C=CH), 5.83 (br s, 0.8 H, C=CH); ^{13}C NMR (150 MHz, $CDCl_3$, 1:1:1:1 mixture of rotamers and *E*/*Z*-conformers): δ = 26.8 (C-9), 27.2 (C-9), 27.5 (C-9), 27.7 (C-9), 28.47 [$C(\mathit{C}H_3)_3$], 28.51 [$C(\mathit{C}H_3)_3$], 28.55 [$C(\mathit{C}H_3)_3$], 28.6

[C(CH$_3$)$_3$], 40.47 (C-1), 40.48 (C-1), 40.57 (C-1), 40.63 (C-1), 41.65 (C-10), 41.66 (C-10), 42.4 (C-10), 42.51 (C-10), 42.53 (C-2 or C-6), 42.55 (C-2 or C-6), 43.3 (C-2 or C-6), 43.5 (C-2 or C-6), 43.9 (C-7), 44.2 (C-7), 44.4 (C-2 or C-6), 44.5 (C-2 or C-6), 44.8 (C-2 or C-6), 45.1 (C-2 or C-6), 45.7 (C-7), 45.8 (C-3), 45.9 (C-3), 46.1 (C-7), 46.2 (C-3), 46.3 (C-5), 46.4 (C-3), 46.7 (C-5), 46.8 (C-5), 47.1 (C-5), 59.2 (OCH_3), 59.4 (OCH_3), 59.5 (OCH_3), 78.5 [*C*(CH_3)$_3$], 78.6 [*C*(CH_3)$_3$], 78.7 [*C*(CH_3)$_3$], 117.0 (C-8), 117.4 (C-8), 118.0 (C-8), 118.1 (C-8), 138.5 (C=*C*H), 138.8 (C=*C*H), 139.7 (C=*C*H), 140.00 (C=*C*H), 153.37 (CO_2N), 153.44 (CO_2N), 153.9 (CO_2N), 154.0 (CO_2N); IR (ATR): $\tilde{\nu}$ = 2944, 2866, 1689, 1412, 1363, 1238, 1217, 1170, 1120, 877 cm^{-1}; HRMS (ESI, pos.): *m/z* calcd for $C_{16}H_{25}NNaO_3$ $[M + Na]^+$: 302.1727; found: 302.1727.

5.2 (1*R*,2*S*,6*R*,7*R*,8*R*)-4-*tert*-Butoxycarbonyl-4-azatricyclo[5.2.1.0^{2,6}]decane-8-carbaldehyde (15)

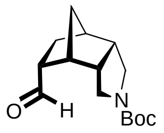

15

Trichloroacetic acid (8.48 g, 51.9 mmol) and water (one drop) were added to a solution of **18** (1.45 g, 5.19 mmol) in CH_2Cl_2 (610 mL). After stirring for 1.5 h at rt, the reaction was treated with saturated aqueous $NaHCO_3$ (570 mL) and extracted with CH_2Cl_2 (3 × 380 mL). The combined organic layers were dried over K_2CO_3 and evaporated under reduced pressure. Column chromatographic purification (silica gel, *n*-pentane/Et_2O 2:1 → 1:2) delivered **15** (1.05 g, 3.96 mmol, 76%) as a colorless oil. $[\alpha]_D^{20}$ = 46.3 (*c* = 0.63, $CHCl_3$); R_f = 0.12 (*n*-pentane/Et_2O 1:1); ^{1}H NMR (400 MHz, $CDCl_3$): δ = 1.42 [s, 9 H, C(CH_3)$_3$], 1.55 (m, 2 H, 10-H, 10-H'), 1.60 (br td, 1 H, *J* = 12.9, 4.6 Hz, 9-H), 1.92 (br dd, 1 H, *J* = 13.3, 5.8 Hz, 9-H'), 2.32 (m, 1 H, 1-H), 2.53 (m, 3 H, 2-H, 6-H, 8-H), 2.78 (br s, 1 H, 7-H), 2.93 (br dd, 1 H, *J* = 12.5, 7.3 Hz, 5-H), 3.00 (dd, 1 H, *J* = 11.9, 8.0 Hz, 3-H), 3.23 (d, 1 H, *J* = 12.3 Hz, 5-H'), 3.53 (br d, 1 H, *J* = 11.6 Hz, 3-H'), 9.79 (s, 1 H, CHO); ^{13}C NMR (100 MHz, $CDCl_3$): δ = 22.4 (C-9), 28.3 [C(CH$_3$)$_3$], 41.0 (C-1), 42.7 (C-2 or C-6), 43.3 (C-10), 43.6 (C-2 or C-6), 43.9 (C-7), 45.8 (C-3), 46.6 (C-5), 53.3 (C-8), 79.4 [*C*(CH_3)$_3$], 154.4 (CO_2N), 203.1 (CHO); IR (ATR): $\tilde{\nu}$ = 2947, 2875, 1691, 1391, 1364, 1230, 1169, 1152, 1140, 1098, 875 cm^{-1}; HRMS (ESI, pos.): *m/z* calcd for $C_{15}H_{23}NNaO_3$ $[M + Na]^+$: 288.1570; found: 288.1570.

5.3 (1*R*,2*S*,6*R*,7*R*,8*R*)-4-*tert*-Butoxycarbonyl-4-azatricyclo[5.2.1.0^{2,6}]decane-8-carboxylic acid (19)

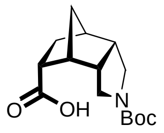

19

The aldehyde **15** (856 mg, 3.23 mmol) was dissolved in MeCN (24 mL). KH_2PO_4 (pH = 4, 2.78 mL) and a solution of H_2O_2 (30%, 1.15 mL, 11.3 mmol) and $NaClO_2$ (642 mg, 7.10 mmol) in water (34 mL) were added. After stirring for 6 h at rt, Na_2SO_3 (350 mg) was added and stirring was continued for 30 min. The solution was acidified to pH = 3 by careful addition of HCl (1 N) and extracted with Et_2O (4 × 100 mL). The combined organic layers were dried over $MgSO_4$ and evaporated under reduced pressure to give the acid **19** (679 mg, 2.41 mmol, 75%) as a white solid after column chromatography (silica gel, CH_2Cl_2/MeOH 99:1 → 95:1). Mp = 64–66 °C; $[\alpha]_D^{20}$ = 4.0 (*c* = 0.28, $CHCl_3$); R_f = 0.17 (CH_2Cl_2/MeOH 95:5); ^{1}H NMR (600 MHz, CD_3OD, 1:1 mixture of rotamers): δ = 1.47 [s, 9 H, C(CH_3)$_3$], 1.53 (m, 3 H, 9-H, 10-H, 10-H'), 1.85 (m, 1 H, 9-H'), 2.29 (br s, 1 H, 1-H), 2.61–2.71 (m,

3 H, 2-H, 6-H, 7-H), 2.79 (m, 1 H, 8-H), 2.97 (m, 1 H, 5-H), 3.06 (m, 1 H, 3-H), 3.50 (br t, 1 H, *J* = 11.4 Hz, 3-H'), 3.72 (d, 0.5 H, *J* = 12.2 Hz, 5-H'), 3.76 (d, 0.5 H, *J* = 12.5 Hz, 5-H'); ^{13}C NMR (150 MHz, CD_3OD, 1:1 mixture of rotamers): δ = 24.1 (C-9), 24.4 (C-9), 28.7 [C(CH_3)$_3$], 29.0 [C(CH_3)$_3$], 42.69 (C-1), 42.73 (C-1), 44.4 (C-2 or C-6), 44.6 (C-2 or C-6), 44.67 (C-10), 44.74 (C-7), 44.78 (C-10), 44.84 (C-7), 45.2 (C-2 or C-6), 45.4 (C-2 or C-6), 45.7 (C-8), 45.9 (C-8), 46.4 (C-5), 46.8 (C-3), 46.9 (C-5), 47.2 (C-3), 80.7 [*C*(CH_3)$_3$], 80.9 [*C*(CH_3)$_3$], 155.9 (CO_2N), 156.4 (CO_2N), 177.4 (CO_2H), 177.2 (CO_2H); IR (ATR): $\tilde{\nu}$ = 2969, 2867, 1726, 1643, 1420, 1365, 1286, 1236, 1194, 1155, 1120, 1095 cm^{-1}; HRMS (ESI, pos.): *m/z* calcd for $C_{15}H_{23}NNaO_4$ [M + Na]$^+$: 304.1519; found: 304.1519.

5.4 (1*R*,2*S*,6*R*,7*R*,8*R*)-4-Azatricyclo[5.2.1.0^{2,6}]decane-8-carboxylic acid hydrochloride (7a•HCl)

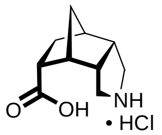

7a•HCl

A suspension of the acid **19** (178 mg, 633μmol) in aqueous HCl (4.8 M, 9.00 mL) was refluxed for 1 d. The solvent was evaporated under reduced pressure, and the crude product was filtered through a pad of silica gel (CH_2Cl_2/MeOH 1:0 → 9:1) affording the amino acid **7a**•HCl (114 mg, 524 μmol, 79%) as a white solid. Mp = 173–175 °C; $[\alpha]_D^{22}$ = –14.5 (*c* = 0.29, MeOH); R_f = 0.06 (MeOH); ^{1}H NMR (600 MHz, CD_3OD): δ= 1.68 (s, 2 H, 10-H, 10-H'), 1.74 (m, 2 H, 9-H, 9-H'), 2.38 (br s, 1 H, 1-H), 2.64 (br s, 1 H, 7-H), 2.78 (m 1 H, 8-H), 2.91 (m, 2 H, 2-H, 6-H), 3.00 (m, 1 H, 5-H), 3.10 (m, 1 H, 3-H), 3.29 (d, 1 H, *J* = 12.6 Hz, 5-H'), 3.52 (d, 1 H, *J* = 12.6 Hz, 3-H'); ^{13}C NMR (150 MHz, CD_3OD): δ = 26.3 (C-9), 42.3 (C-1), 45.14 (C-2 or C-6), 45.15 (C-2 or C-6), 45.4 (C-7), 45.5 (C-10), 46.9 (C-3), 47.0 (C-5), 48.2 (C-8), 183.8 (CO_2H); IR (ATR): $\tilde{\nu}$ = 2950, 2768, 1697, 1558, 1393, 1288, 1206, 1165, 1019, 886 cm^{-1}; HRMS (ESI, pos.): *m/z* calcd for $C_{10}H_{16}NO_2$ [M + H]$^+$: 182.1176; found: 182.1176.

5.5 *N*-[(4-Methylphenyl)sulfonyl] (1*R*,2*S*,6*R*,7*R*,8*R*)-4-*tert*-butoxycarbonyl-4-azatricyclo-[5.2.1.0^{2,6}]decane-8-carboxamide (IV)

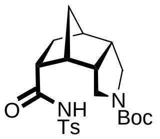

IV

A mixture of acid **19** (177 mg, 629 μmol), DCC (132 mg, 629 μmol) and DMAP (7.00 mg, 62.9 μmol) in anhydrous CH_2Cl_2 (3.0 mL) was stirred at rt for 1 h. $TsNH_2$ (108 mg, 629 μmol) was added and stirring was continued for 1 d. The suspension was filtered through a frit and washed with CH_2Cl_2 (2 × 10 mL). The crude product mixture was filtered through a pad of silica gel (CH_2Cl_2/MeOH 1:0 → 50:1) to give **IV** (175 mg, 403 μmol, 64%) as white solid. Mp = 116–118 °C; $[\alpha]_D^{22}$ = –26.3 (*c* = 0.07, MeOH); R_f = 0.32 (*n*-pentane/EtOAc 1:2); ^{1}H NMR (600 MHz, CD_3OD): δ= 1.38 (m, 1 H, 9-H), 1.52 [m, 9 H, C(CH_3)$_3$], 1.64 (m, 2 H, 10-H, 10-H'), 1.90 (dd, 1 H, *J* = 13.5, 6.6 Hz, 9-H'), 2.24 (m, 1 H, 7-H), 2.42 (s, 3 H, ArCH_3), 2.56 (m, 1 H, 8-H), 2.61–2.78 (m, 3 H, 1-H, 2-H, 6-H), 3.02–3.13 (m, 2 H, 3-H, 5-H), 3.38–3.48 (m, 2 H, 3-H', 5-H'), 7.34 (br d, 2 H, *J* = 8.1 Hz, Ar), 7.86 (br d, 2 H, *J* = 8.2 Hz, Ar); ^{13}C NMR (150 MHz, CD_3OD, mixture of rotamers): δ = 21.6 (ArCH_3), 22.3 (C-9), 28.7 [C(CH_3)$_3$], 42.2 (C-7), 44.9 (CH), 45.1 (CH), 45.7 (C-10), 45.8 (CH), 46.7 (C-3 or C-5), 46.9 (C-3 or C-5), 48.4 (C-8), 81.1 [*C*(CH_3)$_3$], 129.2 (Ar), 130.4 (Ar), 138.5 (Ar), 145.7 (Ar), 156.6 (CO_2N),

173.3 (CONH); IR (ATR): $\tilde{\nu}$ = 2946, 2879, 1691, 1652, 1405, 1363, 1234, 1170, 1120, 1089 cm^{-1}; HRMS (ESI, pos.): *m/z* calcd for $C_{22}H_{30}N_2NaO_5S$ $[M + Na]^+$: 457.1768; found: 457.1768.

5.6 *N*-[(4-Methylphenyl)sulfonyl] (1*R*,2*S*,6*R*,7*R*,8*R*)-4-azatricyclo[5.2.1.0^{2,6}]decane-8-carboxamide hydrochloride (7b•HCl)

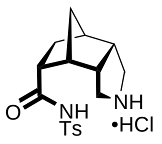

7b•HCl

A solution of the amide **IV** (163 mg, 375 µmol) in ethereal HCl (1.0 M, 16.7 mL, 16.7 mmol) and anhydrous MeOH (1.00 mL) was stirred for 3 h at rt. The precipitate formed was dried in vacuo to yield **7b**•HCl (52.7 mg, 142 µmol, 38%) as a colorless solid. Dp = >210 °C; $[\alpha]_D^{22}$ = –48.1 (*c* = 0.05, MeOH); R_f = 0.47 (MeOH); ^{1}H NMR (600 MHz, CD_3OD): δ = 1.66 (m, 1 H, 9-H), 1.76 (m, 3 H, 9-H', 10-H, 10-H'), 2.38 (br s, 1 H, 1-H), 2.45 (s, 3 H, CH_3), 2.59 (d, 1 H, *J* = 12.1 Hz, 5-H), 2.67 (br s, 1 H, 7-H), 2.83–2.94 (m, 4 H, 2-H, 5-H', 6-H, 8-H), 3.12 (m, 1 H, 3-H), 3.49 (d, 1 H, *J* = 12.1 Hz, 3-H'), 7.42 (dd, 2 H, *J* = 8.6, 0.7 Hz, Ar), 7.92 (dt, 2 H, *J* = 8.5, 1.9 Hz, Ar); ^{13}C NMR (150 MHz, CD_3OD): δ = 21.6 (CH_3), 23.4 (C-9), 41.5 (C-1), 44.9 (C-8), 45.5 (C-6), 45.8 (C-7), 46.1 (C-10), 46.3 (C-5), 47.0 (C-3), 47.1 (C-2), 129.6 (Ar), 130.7 (Ar), 137.6 (Ar), 146.6 (Ar), 177.9 (CONH); IR (ATR): $\tilde{\nu}$ = 2974, 2822, 2710, 2624, 2589, 1671, 1598, 1455, 1335, 1148, 1089, 877, 809 cm^{-1}; HRMS (ESI, pos.): *m/z* calcd for $C_{17}H_{23}N_2O_3S$ $[M + H]^+$: 335.1424; found: 335.1423.

6. Synthesis of the amino acid 8a•HCl and the *N*-tosyl amide 8b•HCl

6.1 (1*R*,2*S*,6*R*,7*R*)-4-*tert*-Butoxycarbonyl-8-(ethoxycarbonylmethylidene)-4-azatricyclo-[5.2.1.0^{2,6}]decane (20)

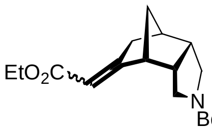

20 (dr = 77:23)

LDA was prepared by adding *n*BuLi (1.6 M in hexanes, 14.4 mL, 23.1 mmol) to a solution of freshly destilled *i*Pr_2NH (2.34 g, 3.24 mL, 23.1 mmol) in anhydrous THF (116 mL) at –78 °C. After 30 min, ethyl 2-(trimethylsilyl)acetate (3.70 g, 4.22 mL, 23.1 mmol) was added dropwise at –78 °C and stirring was continued for 30 min. The ketone **9** (2.90 g, 11.5 mmol), dissolved in anhydrous THF (24 mL), was added slowly to the red reaction mixture at –78 °C and stirring was continued for 1 h. After 18 h at rt, saturated aqueous NH_4Cl (100 mL) was added, and the mixture was extracted with Et_2O (3 × 100 mL). The combined organic layers were dried over $MgSO_4$ and evaporated under reduced pressure. Column chromatographic purification (silica gel, *n*-pentane/Et_2O 4:1 → 3:2) gave **20** (1.87 g, 5.81 mmol, 50%) as a colorless oil. $[\alpha]_D^{20}$ = 232.5 (*c* = 0.36, $CHCl_3$); R_f = 0.33 (*n*-pentane/Et_2O 1:2); ^{1}H NMR (400 MHz, $CDCl_3$, 77:23 mixture of *E*/*Z*-isomers): δ = 1.27 (t, 3 H, *J* = 7.1 Hz, OCH_2CH_3), 1.38 [s, 7 H, $C(CH_3)_3$], 1.42 [s, 2 H, $C(CH_3)_3$], 1.53 (br d, 1 H, *J* = 9.9 Hz, 10-H), 1.60 (br dt, 1 H, *J* = 10.0, 1.6 Hz, 10-H'), 2.11 (m, 1 H, 9-H), 2.23 (m, 1 H, 9-H'), 2.35 (m, 1 H, 1-H), 2.57 (m, 1 H, 2-H), 2.76 (m, 1 H, 6-H), 2.90 (dd, 1 H, *J* = 11.9, 7.7 Hz, 5-H), 2.97 (dd, 1 H, *J* = 12.2, 8.1 Hz, 3-H), 3.42 (d, 1.2 H, *J* = 12.0 Hz, 5-H'), 3.59 (d, 0.8 H, *J* = 12.3 Hz, 3-H'), 3.98 (d, 1 H, *J* = 4.8 Hz, 7-H), 4.07–4.22 (m, 2 H, OCH_2CH_3), 5.62 (br s, 0.2 H, C=CH), 5.65 (br s, 0.8 H, C=CH); ^{13}C NMR (100 MHz, $CDCl_3$, 77:23 mixture of *E*/*Z*-isomers): δ = 14.25 (OCH_2CH_3), 14.32 (OCH_2CH_3), 28.3 [$C(CH_3)_3$], 28.5 [$C(CH_3)_3$], 33.9 (C-9), 34.4 (C-9), 39.3 (C-1), 41.5 (C-10),

41.6 (C-2), 41.7 (C-10), 42.7 (C-2), 44.7 (C-6), 45.7 (C-3), 46.0 (C-3), 46.1 (C-6), 46.4 (C-5), 46.5 (C-5), 47.6 (C-7), 47.8 (C-7), 59.4 (O*C*H_2CH_3), 59.5 (O*C*H_2CH_3), 78.8 [*C*(CH_3)$_3$], 79.0 [*C*(CH_3)$_3$], 112.7 (C=*C*H), 113.2 (C=*C*H), 153.3 (CO_2N), 153.6 (CO_2N), 163.4 (C-8), 165.2 (C-8), 166.7 (*C*O_2CH_2), 166.9 (*C*O_2CH_2); IR (KBr): $\tilde{\nu}$ = 2976, 2901, 2871, 1695, 1661, 1478, 1418, 1366, 1240, 1205, 1176, 1132, 1111, 1038, 876 cm^{-1}; HRMS (ESI, pos.): *m/z* calcd for $C_{18}H_{27}NNaO_4$ $[M + Na]^+$: 344.1832; found: 344.1832.

6.2 Methyl (1*S*,2*S*,6*R*,7*S*,8*S*)-4-*tert*-butoxycarbonyl-4-azatricyclo[5.2.1.0^{2,6}]decane-8-acetate (V)

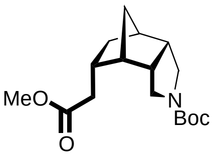

V

Powdered Mg (251 mg, 10.3 mmol) was added at rt to a solution of the α,β-unsaturated ester **20** (1.66 g, 5.16 mmol) in anhydrous MeOH (52 mL). After the gas evolution had ceased, this procedure was repeated several times until all starting material was consumed. The reaction was treated with saturated aqueous NH_4Cl (40 mL) and the aqueous layer was extracted with CH_2Cl_2 (6 × 40 mL). The organic layers were combined, dried over $MgSO_4$, and evaporated under reduced pressure. The methyl ester **V** (1.21 g, 3.91 mmol, 76%) was isolated as a colorless oil after column chromatography (silica gel, *n*-pentane/Et_2O 3:1 → 3:2). $[\alpha]_D^{20}$ = 14.9 (*c* = 0.50, $CHCl_3$); R_f = 0.37 (*n*-pentane/Et_2O 2:1); ^{1}H NMR (600 MHz, $CDCl_3$, 3:2 mixture of rotamers): δ = 1.06 (m, 1 H, 9-H), 1.46 [m, 10 H, C(CH_3)$_3$, 10-H], 1.56 (d, 1 H, *J* = 9.6 Hz, 10-H'), 1.74 (m, 1 H, 9-H'), 2.25 (m, 2 H, 1-H, 7-H), 2.32 (m, 1 H, 8-H), 2.38–2.48 (m, 1 H, C*H*HCO_2), 2.56 (m, 3 H, 2-H, 6-H, CH*H*CO_2), 3.01 (dd, 1 H, *J* = 12.3, 8.2 Hz, 3-H), 3.08 (m, 1 H, 5-H), 3.50 (br d, 0.4 H, *J* = 11.4 Hz, 5-H'), 3.55 (br d, 0.6 H, *J* = 11.9 Hz, 5-H'), 3.64 (m, 3.6 H, 3-H', CO_2CH_3), 3.72 (br d, 0.4 H, *J* = 11.7 Hz, 3-H'); ^{13}C NMR (150 MHz, $CDCl_3$, 3:2 mixture of rotamers): δ = 27.4 (C-9), 28.4 [C(*C*H_3)$_3$], 28.5 [C(*C*H_3)$_3$], 35.9 (*C*H_2CO_2), 36.0 (*C*H_2CO_2), 37.2 (C-8), 37.7 (C-8), 41.4 (C-1 or C-7), 43.0 (C-2 or C-6), 44.0 (C-10), 44.2 (C-2 or C-6), 44.6 (C-1 or C-7), 45.6 (C-5), 45.9 (C-5), 46.1 (C-3), 46.6 (C-3), 51.3 (CO_2*C*H_3), 79.3 [*C*(CH_3)$_3$], 79.4 [*C*(CH_3)$_3$], 154.2 (CO_2N), 174.2 (*C*O_2CH_3), 174.5 (*C*O_2CH_3); IR (ATR): $\tilde{\nu}$ = 2947, 2876, 1735, 1692, 1392, 1365, 1236, 1164, 1125, 1097, 875 cm^{-1}; HRMS (ESI, pos.): *m/z* calcd for $C_{17}H_{27}NNaO_4$ $[M + Na]^+$: 332.1832; found: 332.1832.

6.3 (1*S*,2*S*,6*R*,7*S*,8*S*)-4-*tert*-Butoxycarbonyl-4-azatricyclo[5.2.1.0^{2,6}]decane-8-acetic acid (21)

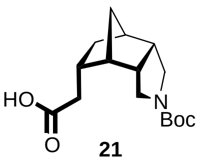

21

A solution of **V** (1.12 g, 3.62 mmol) and KOH (4.06 g, 72.4 mmol) in aqueous EtOH (50%, 24 mL) was refluxed for 1 d. Water (30 mL) was added at rt and the mixture was extracted with Et_2O (2 × 30 mL). The pH of the aqueous layer was adjusted to 4 by addition of HCl (1 N). The white suspension was extracted with EtOAc (2 × 100 mL) and the combined organic layers were dried over $MgSO_4$ and evaporated under reduced pressure to provide the acid **21** (959 mg, 3.25 mmol, 90%) as a colorless solid. Dp = 160 °C; $[\alpha]_D^{20}$ = 14.2 (*c* = 0.41, $CHCl_3$); R_f = 0.24 (*n*-pentane/Et_2O 1:1); ^{1}H NMR (600 MHz, $CDCl_3$): δ = 1.05 (m, 1 H, 9-H), 1.49 [s, 9 H, C(CH_3)$_3$], 1.53 (dd, 1 H, *J* = 9.8, 1.3 Hz, 10-H), 1.59 (d, 1 H, *J* = 9.5 Hz, 10-H'), 1.78 (m, 1 H, 9-H'), 2.29 (m, 3 H, 1-H, 7-H, 8-H), 2.40 (m, 1 H, C*H*HCO_2), 2.53 (m, 1 H, CH*H*CO_2), 2.57–2.69 (m, 2 H, 2-H, 6-H), 2.98–3.14 (m, 2 H, 3-

H, 5-H), 3.53 (m, 1 H, 3-H'), 3.74 (m, 1 H, 5-H'); ^{13}C NMR (150 MHz, $CDCl_3$, mixture of rotamers): δ = 28.6 (C-9), 28.7 [C(CH$_3$)$_3$], 28.8 (C-9), 28.9 [C(CH$_3$)$_3$], 37.1 (CH_2CO_2), 37.2 (CH_2CO_2), 38.8 (C-8), 39.1 (C-8), 42.8 (C-1), 44.4 (C-2), 44.5 (C-2), 45.0 (C-10), 45.4 (C-6), 45.7 (C-7), 46.1 (C-7), 46.7 (C-3), 47.2 (C-3), 47.2 (C-5), 47.7 (C-5), 81.0 [C(CH$_3$)$_3$], 81.3 [C(CH$_3$)$_3$], 156.0 (CO_2N), 177.2 (CO_2H), 177.4 (CO_2H); IR (ATR): $\tilde{\nu}$ = 3219, 2939, 2880, 1733, 1658, 1421, 1241, 1168, 1160, 1143, 1132 cm^{-1}; HRMS (ESI, pos.): *m/z* calcd for $C_{16}H_{25}NNaO_4$ [M + Na]$^+$: 318.1676; found: 318.1676.

6.4 (1*S*,2*S*,6*R*,7*S*,8*S*)-4-Azatricyclo[5.2.1.0^{2,6}]decane-8-acetic acid hydrochloride (8a•HCl)

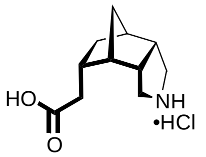

8a•HCl

A suspension of the acid **21** (154 mg, 521 μmol) in aqueous HCl (4.8 M, 6.40 mL) was refluxed for 1d. The solution was concentrated under reduced pressure and the crude product was filtered through a pad of silica gel (CH_2Cl_2/MeOH 1:0 → 9:1). The amino acid **8a**•HCl (86.0 mg, 371 μmol, 71%) was obtained as a white solid. Crystallization from MeOH/Et_2O gave colorless cubic crystals. Mp = 156–158 °C; $[\alpha]_D^{20}$ = 7.5 (*c* = 1.0, $CHCl_3$); R_f = 0.08 (MeOH); ^1H NMR (600 MHz, CD_3OD): δ = 1.19 (ddd, 1 H, *J* = 13.9, 7.0, 1.7 Hz, 9-H), 1.76 (m, 2 H, 10-H, 10-H'), 1.96 (m, 1 H, 9-H'), 2.36 (m, 2 H, 1-H, 7-H), 2.51 (m, 1 H, 8-H), 2.60 (m, 2 H, CH_2CO$_2$), 2.92 (m, 2 H, 2-H, 6-H), 3.25 (m, 3 H, 3-H, 5-H, 5-H'), 3.49 (m, 1 H, 3-H'); ^{13}C NMR (150 MHz, CD_3OD): δ = 28.4 (C-9), 37.9 (CH_2CO_2), 39.0 (C-8), 41.3 (C-1), 44.7 (C-7), 45.7 (C-6), 46.1 (C-5), 46.8 (C-2), 47.0 (C-3), 47.1 (C-10), 174.9 (CO_2H); IR (ATR): $\tilde{\nu}$ = 2883, 2736, 1733, 1429, 1320, 1196, 1175, 1149, 989, 879 cm^{-1}; HRMS (ESI, pos.): *m/z* calcd for $C_{11}H_{18}NO_2$ [M + H]$^+$: 196.1332; found: 196.1336.

6.5 *N*-[(4-Methylphenyl)sulfonyl] (1*S*,2*S*,6*R*,7*S*,8*S*)-4-*tert*-butoxycarbonyl-4-azatricyclo-[5.2.1.0^{2,6}]decane-8-acetamide (VI)

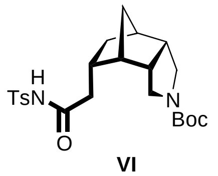

VI

A mixture of the acid **21** (355 mg, 1.20 mmol), DCC (252 mg, 1.20 mmol), and DMAP (13.5 mg, 120 μmol) in anhydrous CH_2Cl_2 (6.0 mL) was stirred at rt for 1 h. $TsNH_2$ (206 mg, 1.20 mmol) was added and stirring was continued for 4 d. The suspension was filtered through a frit and washed with CH_2Cl_2 (2 × 10 mL). The crude product mixture was filtered through a pad of silica gel (CH_2Cl_2/MeOH 1:0 → 50:1) to yield **VI** (390 mg, 870 μmol, 72%) as a white solid. Mp = 84–86 °C; $[\alpha]_D^{21}$ = –15.7 (*c* = 0.1, $CHCl_3$); R_f = 0.24 (*n*-pentane/Et_2O 1:1); ^1H NMR (600 MHz, $CDCl_3$): δ = 0.88 (br s, 1 H, 9-H), 1.41 (br d, 1 H, *J* = 9.5 Hz, 10-H), 1.46 [s, 9 H, C(CH$_3$)$_3$], 1.50 (br d, 1 H, *J* = 9.7 Hz, 10-H'), 1.75 (br s, 1 H, 9-H'), 2.15 (br s, 1 H, 7-H), 2.22 (t, 1 H, *J* = 4.8 Hz, 1-H), 2.28 (m, 1 H, 8-H), 2.36 (m, 2 H, CH_2CONH), 2.42 (s, 3 H, ArCH$_3$), 2.46 (m, 1 H, 6-H), 2.56 (m, 1 H, 2-H), 2.98 (dd, 1 H, *J* = 12.6, 8.9 Hz, 5-H), 3.07 (t, 1 H, *J* = 10.2 Hz, 3-H), 3.48 (d, 1 H, *J* = 12.1 Hz, 3-H'), 3.54 (d, 1 H, *J* = 12.6 Hz, 5-H'), 7.30 (d, 2 H, *J* = 8.1 Hz, Ar), 7.93 (d, 2 H, *J* = 8.3 Hz, Ar); ^{13}C NMR (150 MHz, $CDCl_3$): δ = 21.7 (ArCH$_3$), 27.8 (C-9), 28.6 [C(CH$_3$)$_3$], 35.6 (C-8), 37.9 (CH$_2$CONH), 41.3 (C-1), 42.8 (C-6), 43.8 (C-10), 44.0 (C-2), 44.1 (C-7), 45.9 (C-3), 46.2 (C-5), 80.1 [C(CH$_3$)$_3$], 128.4 (Ar), 129.4 (Ar), 136.0 (Ar), 144.6 (Ar), 154.8 (CO_2N), 170.7 (CONH); IR

(ATR): $\tilde{\nu}$ = 2946, 2875, 1657, 1412, 1343, 1240, 1169, 1130, 1087, 866 cm^{-1}; HRMS (ESI, neg.): *m/z* calcd for $C_{23}H_{31}N_2O_5S$ $[M-H]^-$: 447.1959; found: 447.1960.

6.6 *N*-[(4-Methylphenyl)sulfonyl] (1*S*,2*S*,6*R*,7*S*,8*S*)-4-azatricyclo[5.2.1.0^{2,6}]decane-8-acetamide hydrochloride (8b•HCl)

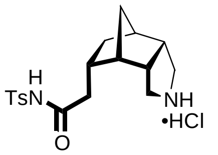

8b•HCl

The amide **VI** (100 mg, 223 μmol) was dissolved in an ethereal solution of HCl (1.0 M, 8.50 mL, 8.50 mmol) and stirred for 20 h at rt. The precipitate formed was collected and dried to give **8b**•HCl (36.3 mg, 94.3 μmol, 42%) as a colorless solid. Mp = 135–137 °C; $[\alpha]_D^{22}$ = –18.9 (*c* = 0.05, MeOH); R_f = 0.56 (MeOH); ^{1}H NMR (600 MHz, CD_3OD): δ = 1.12 (ddd, 1 H, *J* = 13.9, 7.1, 2.1 Hz, 9-H), 1.68 (dt, 1 H, *J* = 9.8, 2.3 Hz, 10-H), 1.71 (dd, 1 H, *J* = 9.9, 1.7 Hz, 10-H'), 1.83 (m, 1 H, 9-H'), 2.18 (br s, 1 H, 7-H), 2.32 (br t, 1 H, *J* = 4.4 Hz, 1-H), 2.36 (m, 1 H, 8-H), 2.44 (m, 4 H, C*H*HCONH, CH_3), 2.48 (dd, 1 H, *J* = 14.8, 9.5 Hz, CH*H*CONH), 2.81–2.92 (m, 2 H, 2-H, 6-H), 3.21 (m, 2 H, 3-H, 5-H), 3.26 (dd, 1 H, *J* = 12.5, 8.6 Hz, 3-H'), 3.46 (dd, 1 H, *J* = 12.9, 6.3 Hz, 5-H'), 7.41 (dd, 2 H, *J* = 8.6, 0.7 Hz, Ar), 7.89 (dd, 2 H, *J* = 8.5, 1.9 Hz, Ar); ^{13}C NMR (150 MHz, CD_3OD): δ = 21.6 (CH_3), 28.2 (C-9), 38.9 (C-8), 39.9 (*C*H_2CONH), 41.2 (C-1), 44.6 (C-7), 45.5 (C-6), 46.0 (C-3), 46.7 (C-2), 46.95 (C-5), 46.97 (C-10), 129.3 (Ar), 130.6 (Ar), 137.9 (Ar), 146.3 (Ar), 172.7 (CONH); IR (ATR): $\tilde{\nu}$ = 2950, 1714, 1595, 1439, 1338, 1167, 1085, 855, 815, 660 cm^{-1}; HRMS (ESI, pos.): *m/z* calcd for $C_{18}H_{25}N_2O_3S$ $[M+H]^+$: 349.15804; found: 349.15804.
