# Supplementary material for: Enantioselective synthesis of tricyclic amino acid derivatives based on a rigid 4-azatricyclo[5.2.1.02,6]decane skeleton
Source: Beilstein J Org Chem. 2009 Dec 21;5:81. doi: 10.3762/bjoc.5.81 (PMC2839531; doi:10.3762/bjoc.5.81)
Supplement: File 2 — NMR spectra of all new compounds. [file Beilstein_J_Org_Chem-05-81-s002.pdf]

Supporting Information File 2:

NMR spectra of all new compounds

Enantioselective synthesis of tricyclic amino acid derivatives based on a rigid 4-azatricyclo[5.2.1.0^{2,6}]decane skeleton

Matthias Breuning*[,1], Tobias Häuser[1], Christian Mehler[1], Christian Däschlein[2], Carsten Strohmann[2], Andreas Oechsner[3] and Holger Braunschweig[3]

Address: [1]Institut für Organische Chemie, Universität Würzburg, Am Hubland, 97074 Würzburg, Germany, [2]Anorganische Chemie, Universität Dortmund, Otto-Hahn-Str. 6, 44227 Dortmund, Germany and [3]Institut für Anorganische Chemie, Universität Würzburg, Am Hubland, 97074 Würzburg, Germany

Email: Matthias Breuning* - breuning@chemie.uni-wuerzburg.de

* Corresponding author

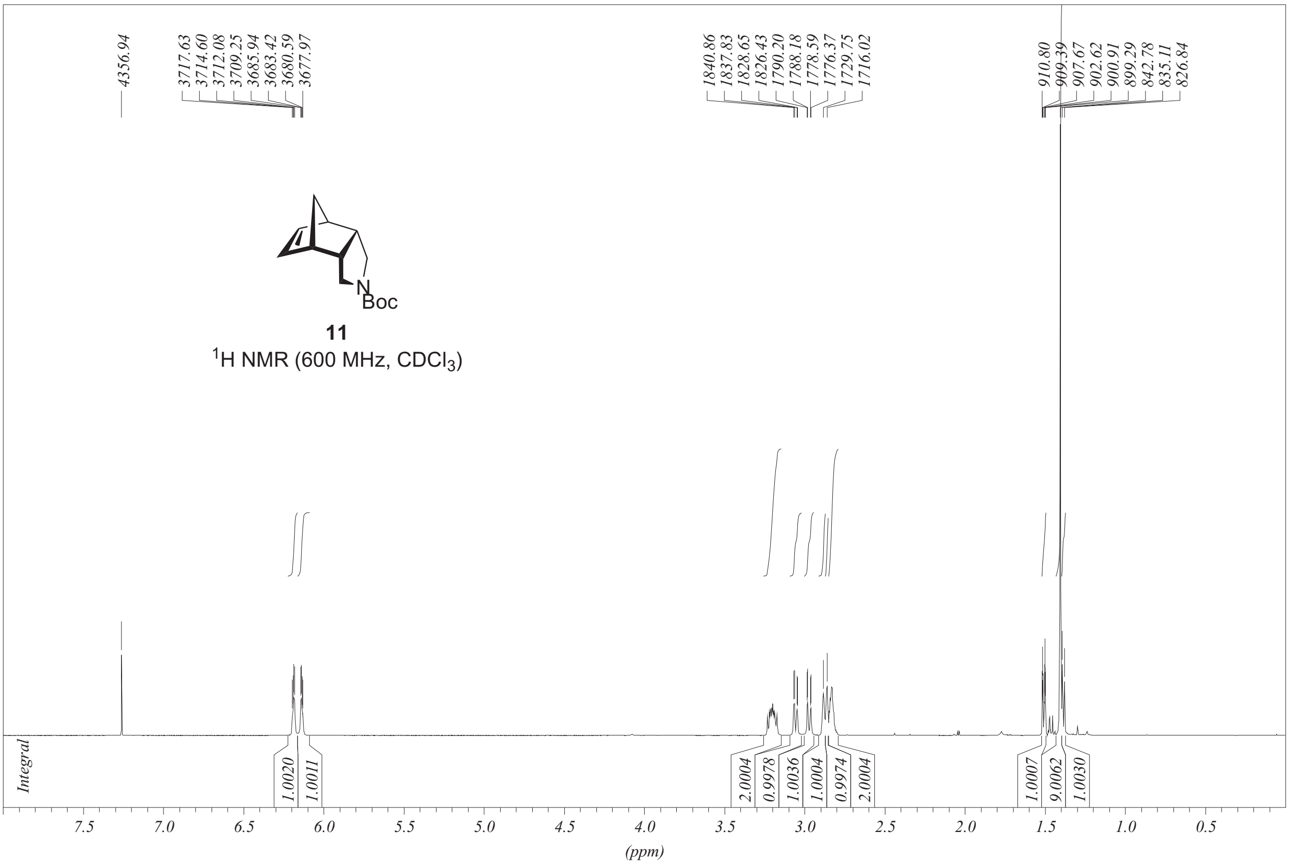
4356.94
3717.63
3714.60
3712.08
3709.25
3685.94
3683.42
3680.59
3677.97
1840.86
1837.83
1828.65
1826.43
1790.20
1788.18
1778.59
1776.37
1729.75
1716.02
910.80
909.39
907.67
902.62
900.91
899.29
842.78
835.11
826.84
N
Boc
11
1H NMR (600 MHz, CDCl3)
Integral
1.0020
1.0011
2.0004
0.9978
1.0036
1.0004
0.9974
2.0004
1.0007
9.0062
1.0030
7.5
7.0
6.5
6.0
5.5
5.0
4.5
4.0
3.5
3.0
2.5
2.0
1.5
1.0
0.5
(ppm)

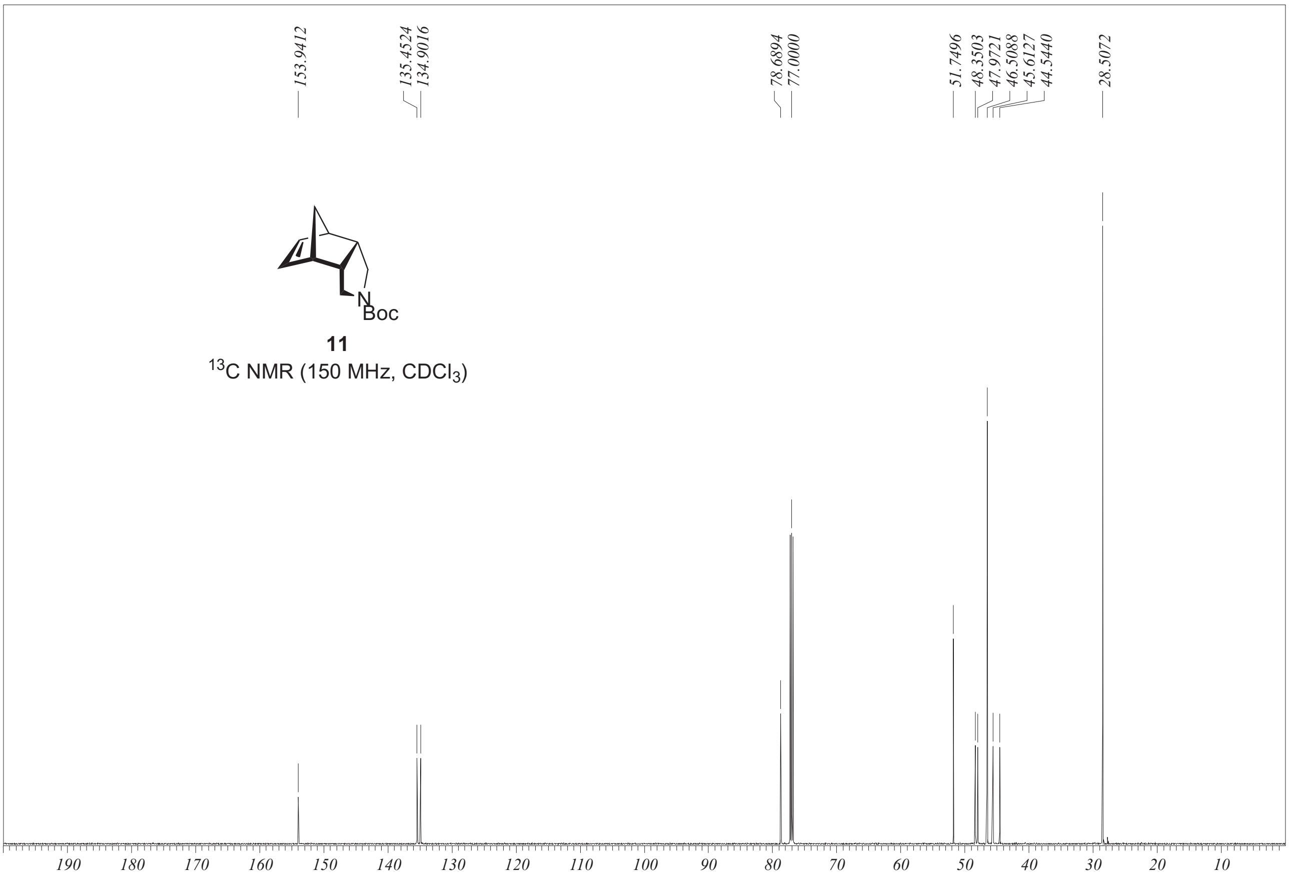
153.9412
135.4524
134.9016
78.6894
77.0000
51.7496
48.3503
47.9721
46.5088
45.6127
44.5440
28.5072
N
Boc
11
13C NMR (150 MHz, CDCl3)
190
180
170
160
150
140
130
120
110
100
90
80
70
60
50
40
30
20
10
(ppm)

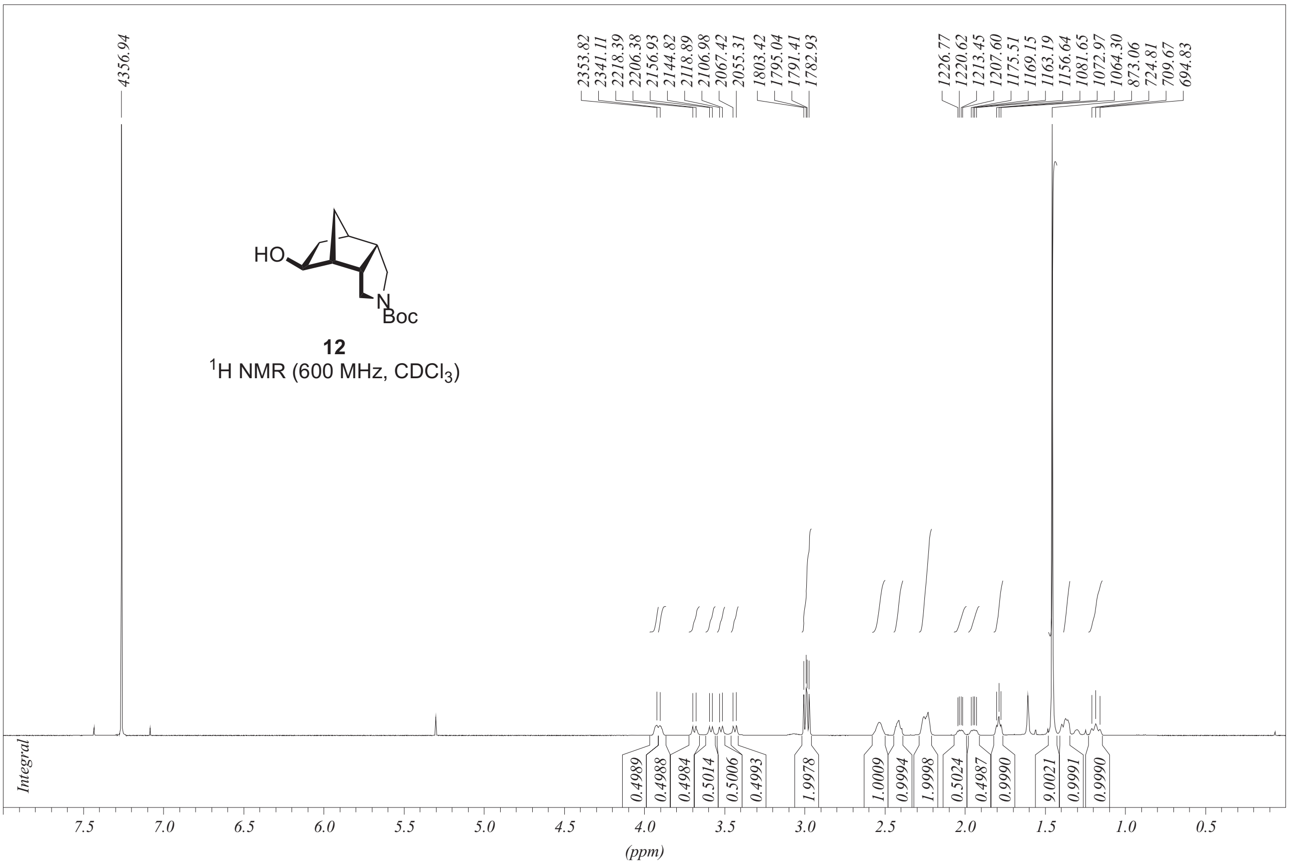
4356.94
2353.82 2341.11 2218.39 2206.38 2156.93 2144.82 2118.89 2106.98 2067.42 2055.31 1803.42 1795.04 1791.41 1782.93
1226.77 1220.62 1213.45 1207.60 1175.51 1169.15 1163.19 1156.64 1081.65 1072.97 1064.30 873.06 724.81 709.67 694.83
HO
N
Boc
12
1H NMR (600 MHz, CDCl3)
Integral
(ppm)

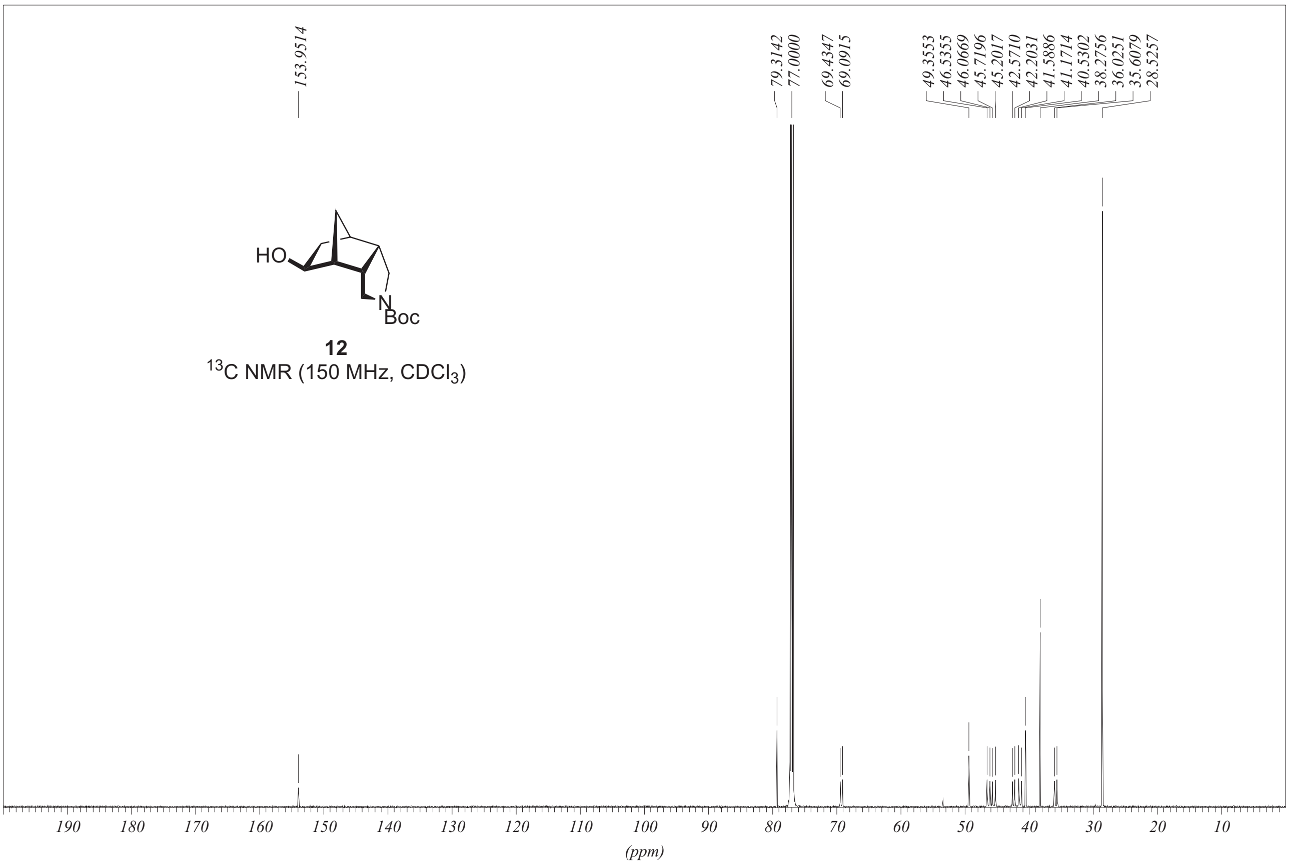
153.9514
79.3142 77.0000 69.4347 69.0915
49.3553 46.5355 46.0669 45.7196 45.2017 42.5710 42.2031 41.5886 41.1714 40.5302 38.2756 36.0251 35.6079 28.5257
HO
N
Boc
12
13C NMR (150 MHz, CDCl3)
(ppm)

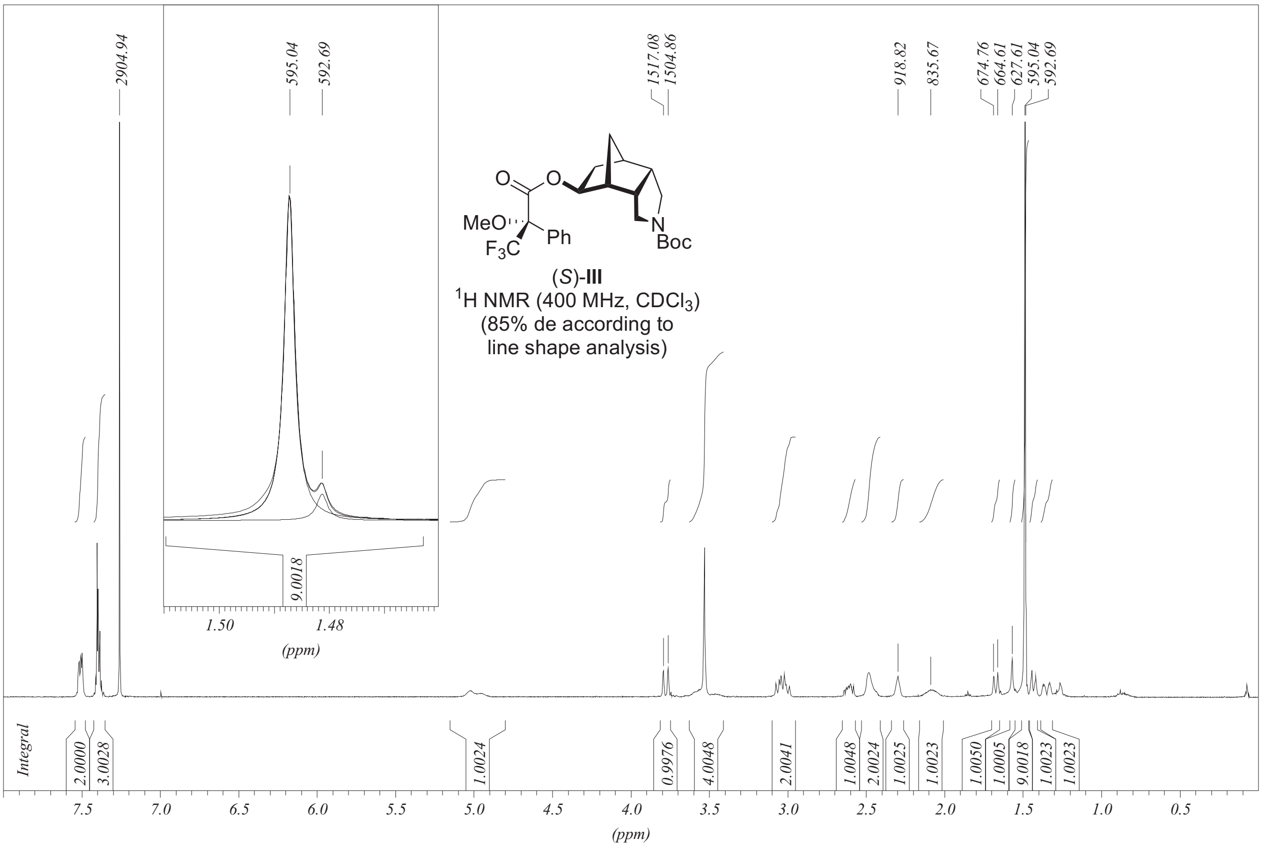
(S)-III
^{1}H NMR (400 MHz, CDCl$_3$)
(85% de according to
line shape analysis)
MeO
F3C
Ph
Boc
N
O
2904.94
595.04
592.69
1517.08
1504.86
918.82
835.67
674.76
664.61
627.61
595.04
592.69
9.0018
1.50
1.48
(ppm)
Integral
2.0000
3.0028
1.0024
0.9976
4.0048
2.0041
1.0048
2.0024
1.0025
1.0023
1.0050
1.0005
9.0018
1.0023
1.0023
7.5
7.0
6.5
6.0
5.5
5.0
4.5
4.0
3.5
3.0
2.5
2.0
1.5
1.0
0.5
(ppm)

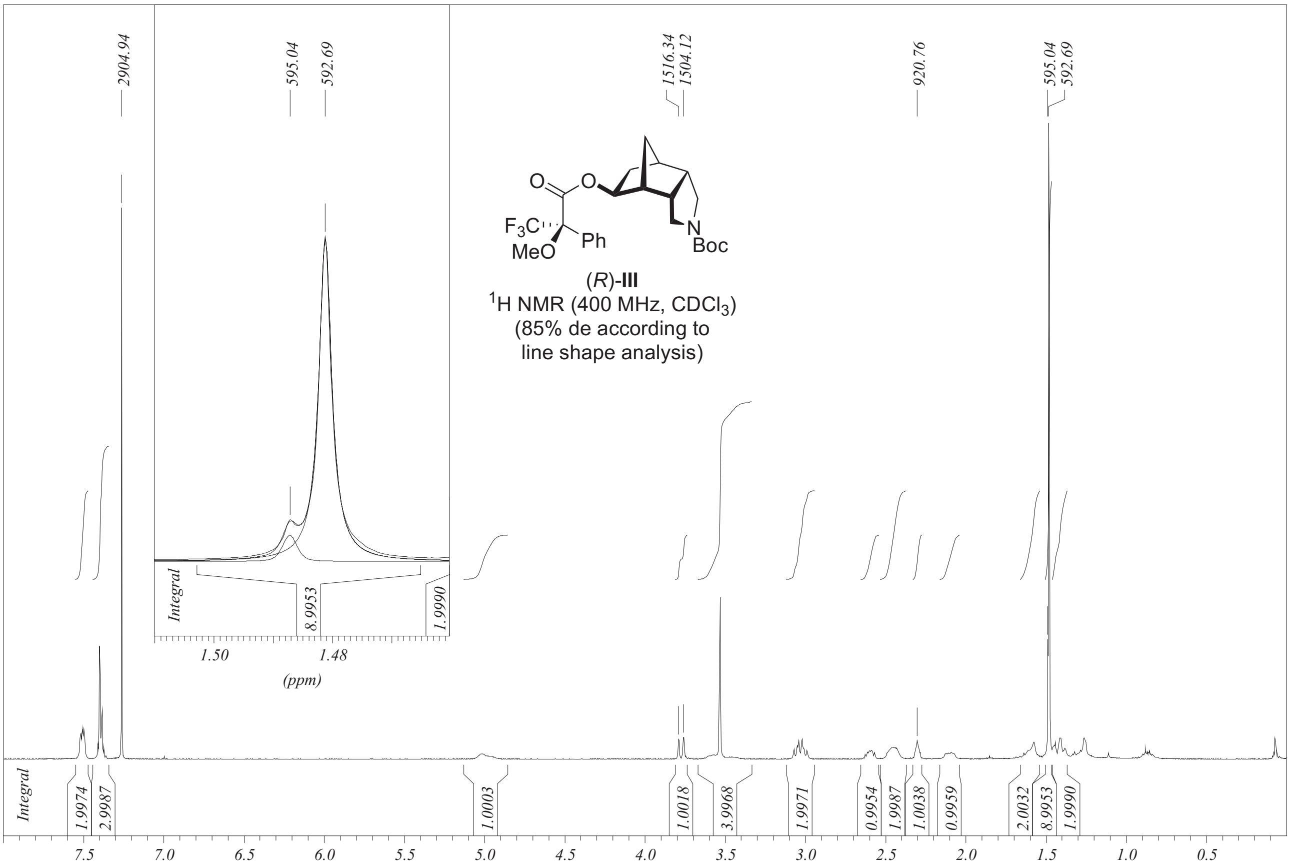
(R)-III
^{1}H NMR (400 MHz, CDCl$_3$)
(85% de according to
line shape analysis)
F3C
MeO
Ph
Boc
N
O
2904.94
595.04
592.69
1516.34
1504.12
920.76
595.04
592.69
Integral
8.9953
1.9990
1.50
1.48
(ppm)
Integral
1.9974
2.9987
1.0003
1.0018
3.9968
1.9971
0.9954
1.9987
1.0038
0.9959
2.0032
8.9953
1.9990
7.5
7.0
6.5
6.0
5.5
5.0
4.5
4.0
3.5
3.0
2.5
2.0
1.5
1.0
0.5
(ppm)

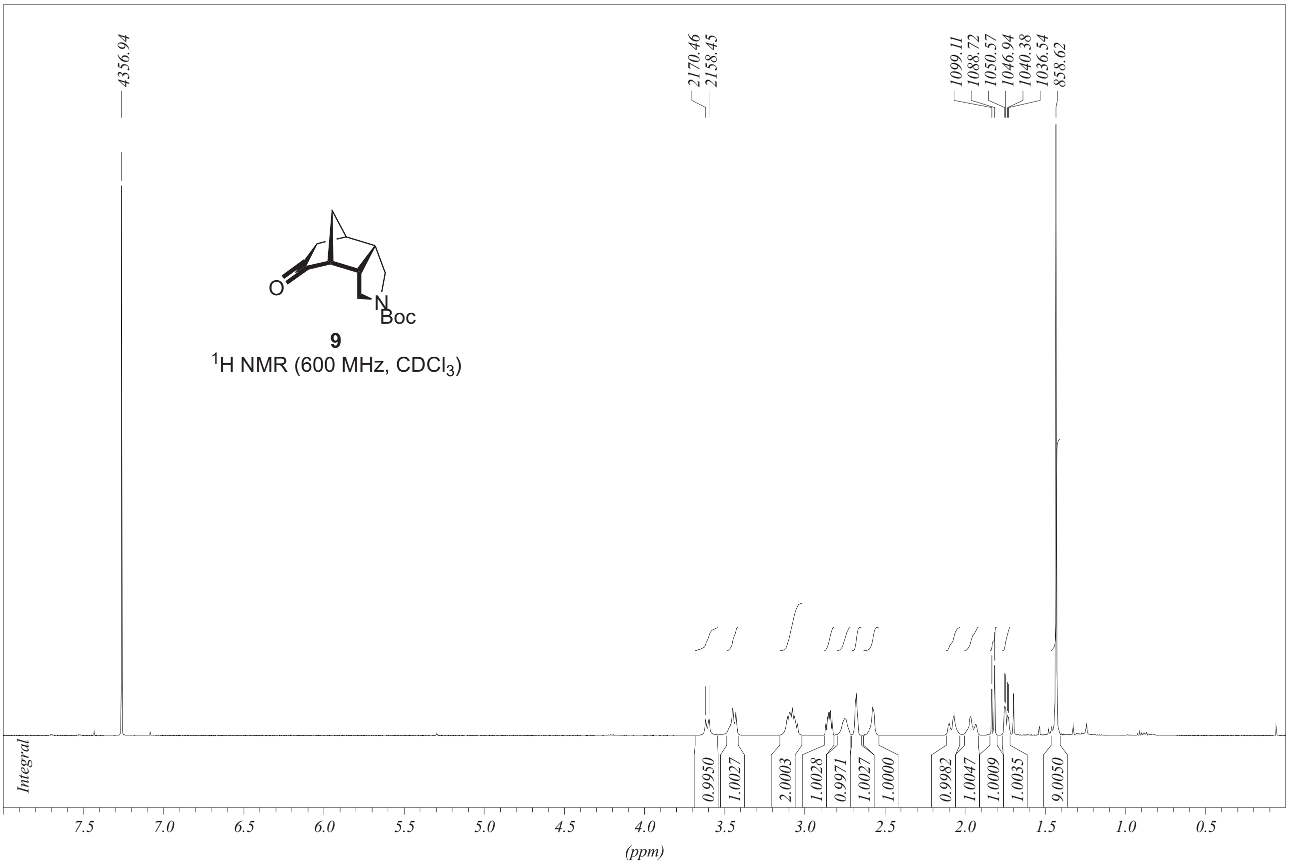
4356.94
2170.46
2158.45
1099.11
1088.72
1050.57
1046.94
1040.38
1036.54
858.62
O
N
Boc
9
^{1}H NMR (600 MHz, $CDCl_3$)
Integral
0.9950
1.0027
2.0003
1.0028
0.9971
1.0027
1.0000
0.9982
1.0047
1.0009
1.0035
9.0050
7.5 7.0 6.5 6.0 5.5 5.0 4.5 4.0 3.5 3.0 2.5 2.0 1.5 1.0 0.5
(ppm)

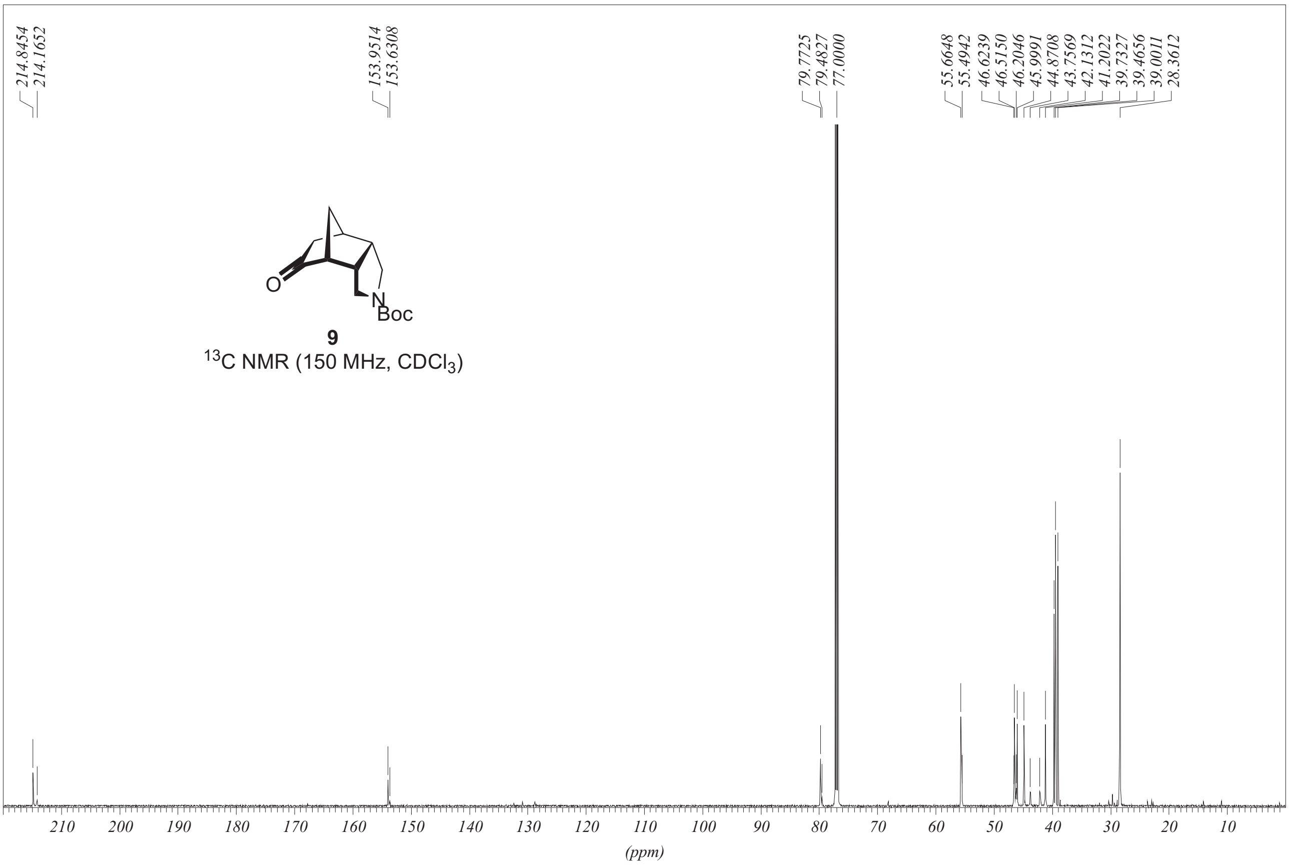
214.8454
214.1652
153.9514
153.6308
79.7725
79.4827
77.0000
55.6648
55.4942
46.6239
46.5150
46.2046
45.9991
44.8708
43.7569
42.1312
41.2022
39.7327
39.4656
39.0011
28.3612
O
N
Boc
9
^{13}C NMR (150 MHz, $CDCl_3$)
210 200 190 180 170 160 150 140 130 120 110 100 90 80 70 60 50 40 30 20 10
(ppm)

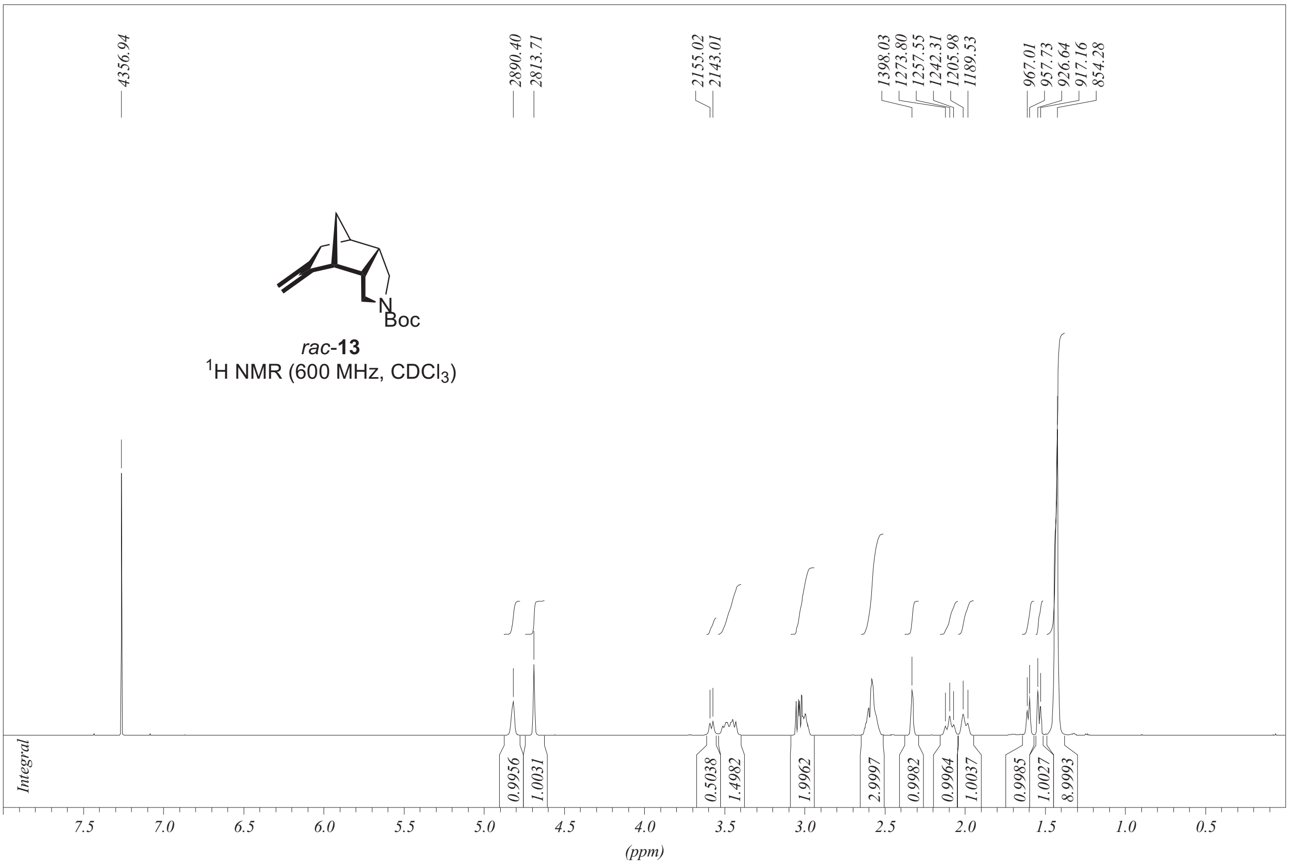
4356.94
2890.40
2813.71
2155.02
2143.01
1398.03
1273.80
1257.55
1242.31
1205.98
1189.53
967.01
957.73
926.64
917.16
854.28
Boc
rac-13
^{1}H NMR (600 MHz, $CDCl_3$)
Integral
0.9956
1.0031
0.5038
1.4982
1.9962
2.9997
0.9982
0.9964
1.0037
0.9985
1.0027
8.9993
7.5
7.0
6.5
6.0
5.5
5.0
4.5
4.0
3.5
3.0
2.5
2.0
1.5
1.0
0.5
(ppm)

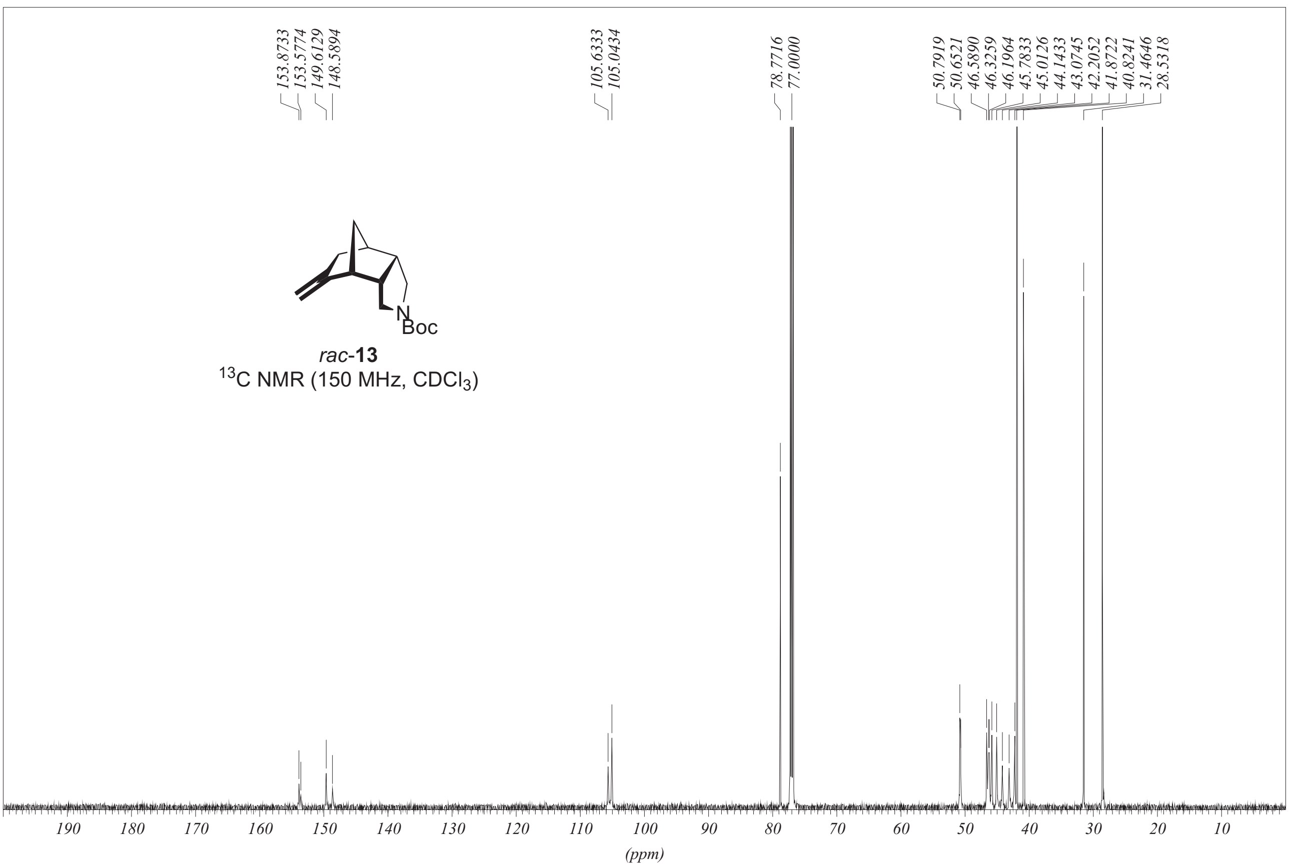
153.8733
153.5774
149.6129
148.5894
105.6333
105.0434
78.7716
77.0000
50.7919
50.6521
46.5890
46.3259
46.1964
45.7833
45.0126
44.1433
43.0745
42.2052
41.8722
40.8241
31.4646
28.5318
Boc
rac-13
^{13}C NMR (150 MHz, $CDCl_3$)
190
180
170
160
150
140
130
120
110
100
90
80
70
60
50
40
30
20
10
(ppm)

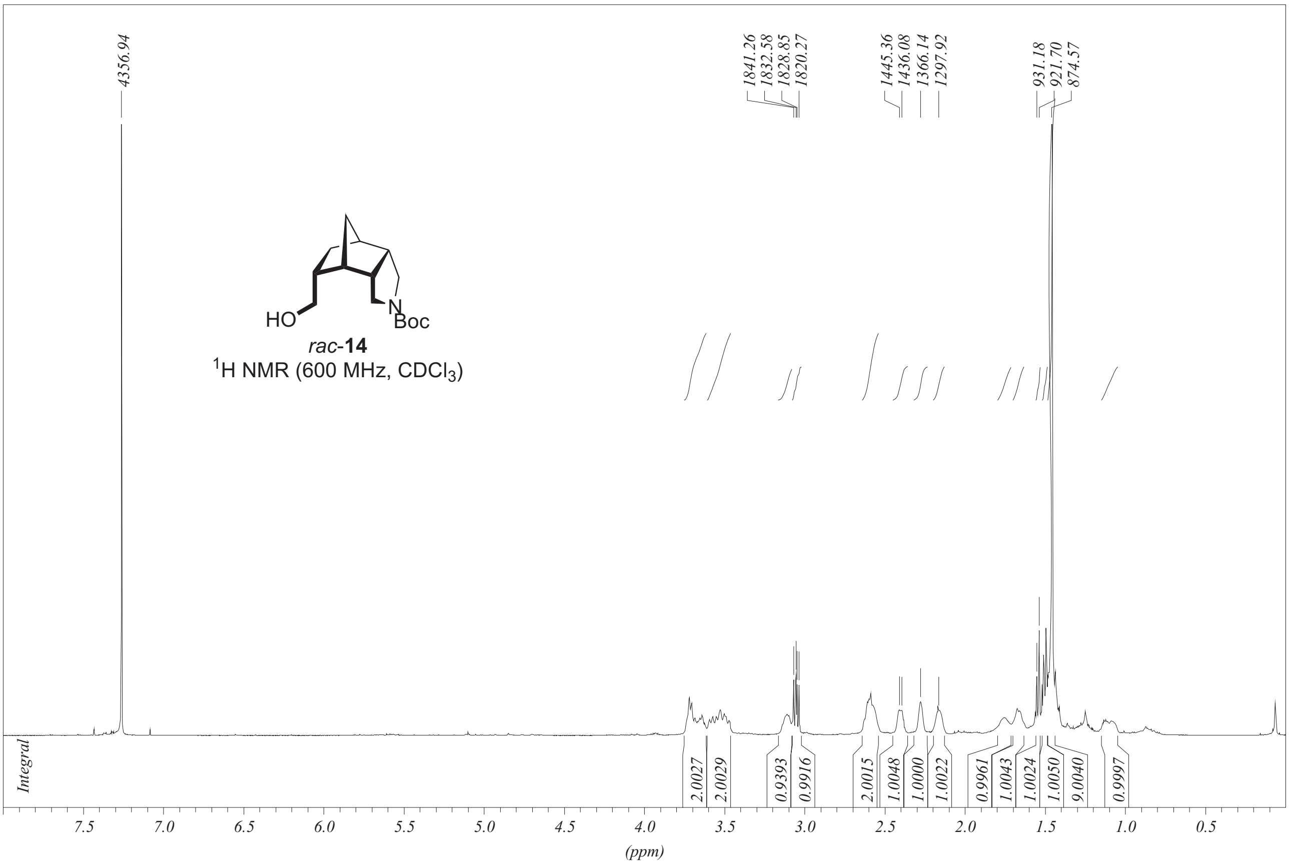
4356.94
1841.26
1832.58
1828.85
1820.27
1445.36
1436.08
1366.14
1297.92
931.18
921.70
874.57
HO
N
Boc
rac-14
1H NMR (600 MHz, CDCl3)
Integral
2.0027
2.0029
0.9393
0.9916
2.0015
1.0048
1.0000
1.0022
0.9961
1.0043
1.0024
1.0050
9.0040
0.9997
7.5
7.0
6.5
6.0
5.5
5.0
4.5
4.0
3.5
3.0
2.5
2.0
1.5
1.0
0.5
(ppm)

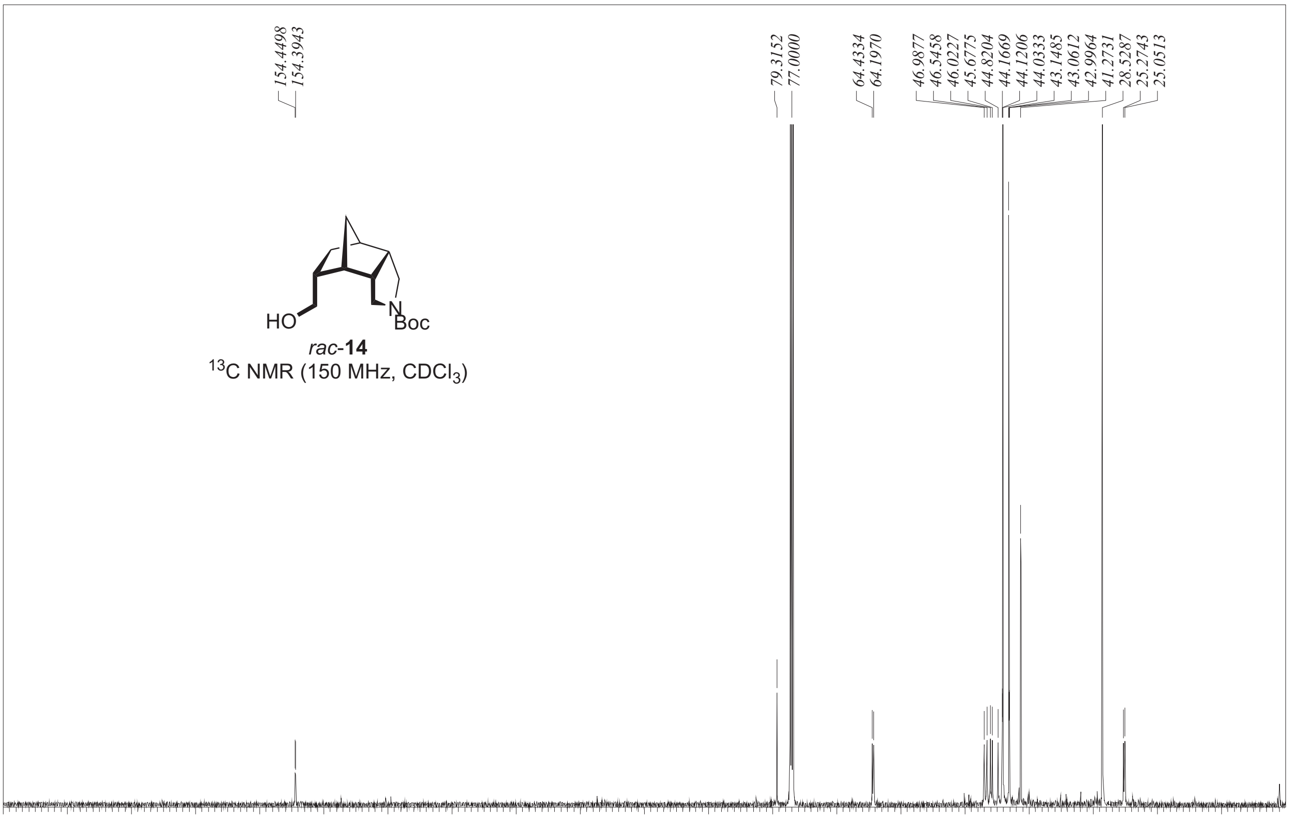
154.4498
154.3943
79.3152
77.0000
64.4334
64.1970
46.9877
46.5458
46.0227
45.6775
44.8204
44.1669
44.1206
44.0333
43.1485
43.0612
42.9964
41.2731
28.5287
25.2743
25.0513
HO
N
Boc
rac-14
13C NMR (150 MHz, CDCl3)
190
180
170
160
150
140
130
120
110
100
90
80
70
60
50
40
30
20
10
(ppm)

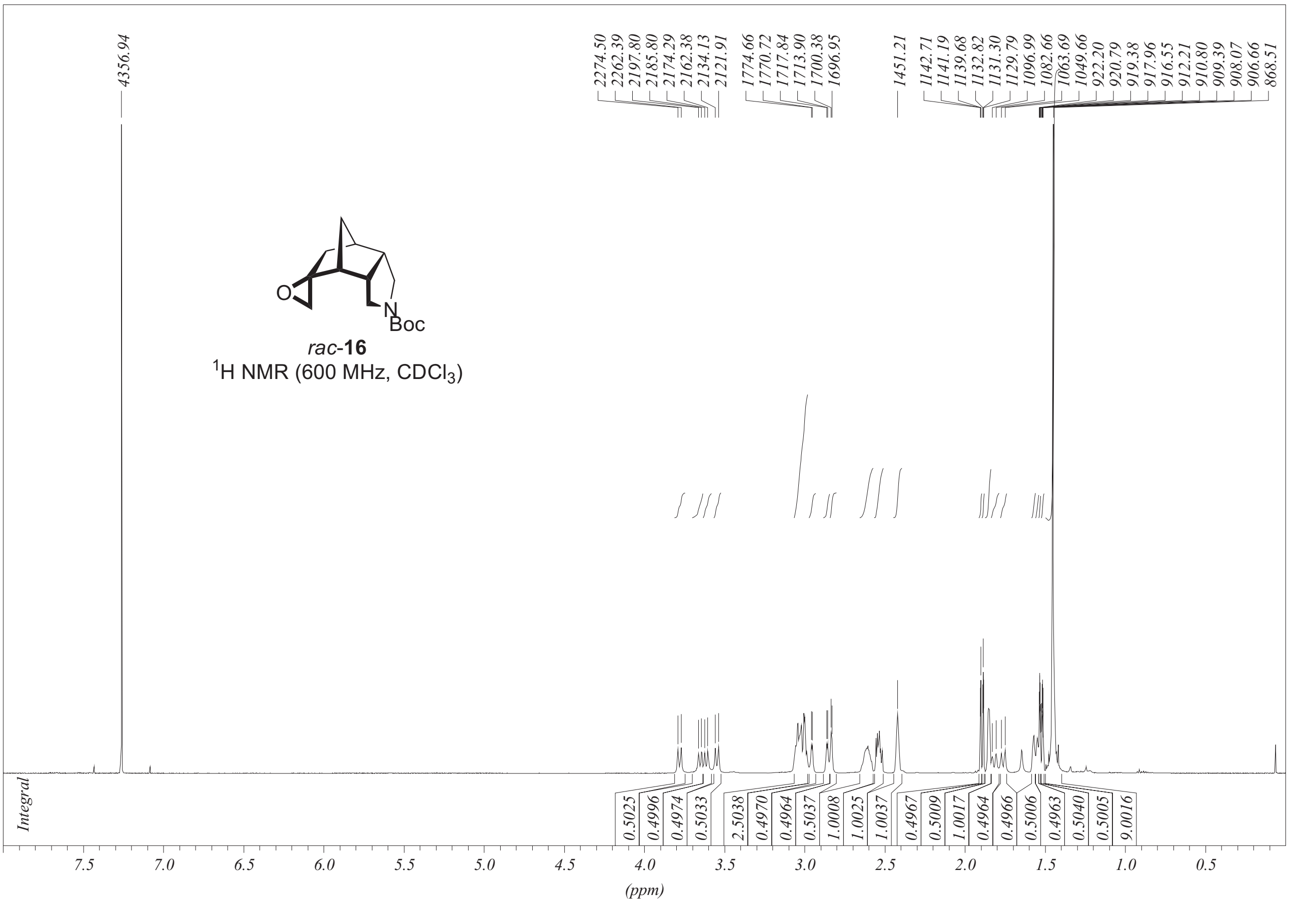
4356.94
2274.50
2262.39
2197.80
2185.80
2174.29
2162.38
2134.13
2121.91
1774.66
1770.72
1717.84
1713.90
1700.38
1696.95
1451.21
1142.71
1141.19
1139.68
1132.82
1131.30
1129.79
1096.99
1082.66
1063.69
1049.66
922.20
920.79
919.38
917.96
916.55
912.21
910.80
909.39
908.07
906.66
868.51
O
N
Boc
rac-**16**
^{1}H NMR (600 MHz, $CDCl_3$)
Integral
0.5025
0.4996
0.4974
0.5033
2.5038
0.4970
0.4964
0.5037
1.0008
1.0025
1.0037
0.4967
0.5009
1.0017
0.4964
0.4966
0.5006
0.4963
0.5040
0.5005
9.0016
7.5 7.0 6.5 6.0 5.5 5.0 4.5 4.0 3.5 3.0 2.5 2.0 1.5 1.0 0.5
(ppm)

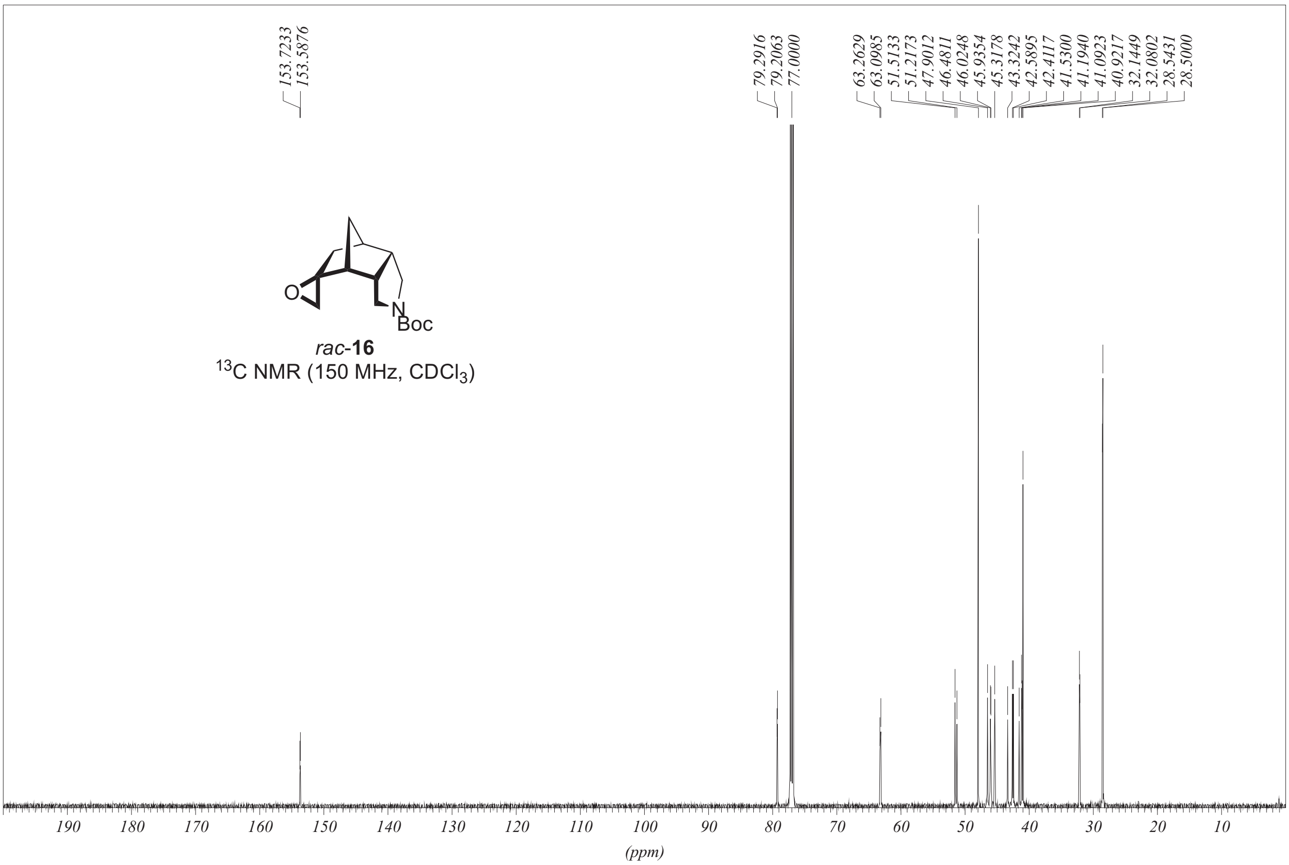
153.7233
153.5876
79.2916
79.2063
77.0000
63.2629
63.0985
51.5133
51.2173
47.9012
46.4811
46.0248
45.9354
45.3178
43.3242
42.5895
42.4117
41.5300
41.1940
41.0923
40.9217
32.1449
32.0802
28.5431
28.5000
O
N
Boc
rac-**16**
^{13}C NMR (150 MHz, $CDCl_3$)
190 180 170 160 150 140 130 120 110 100 90 80 70 60 50 40 30 20 10
(ppm)

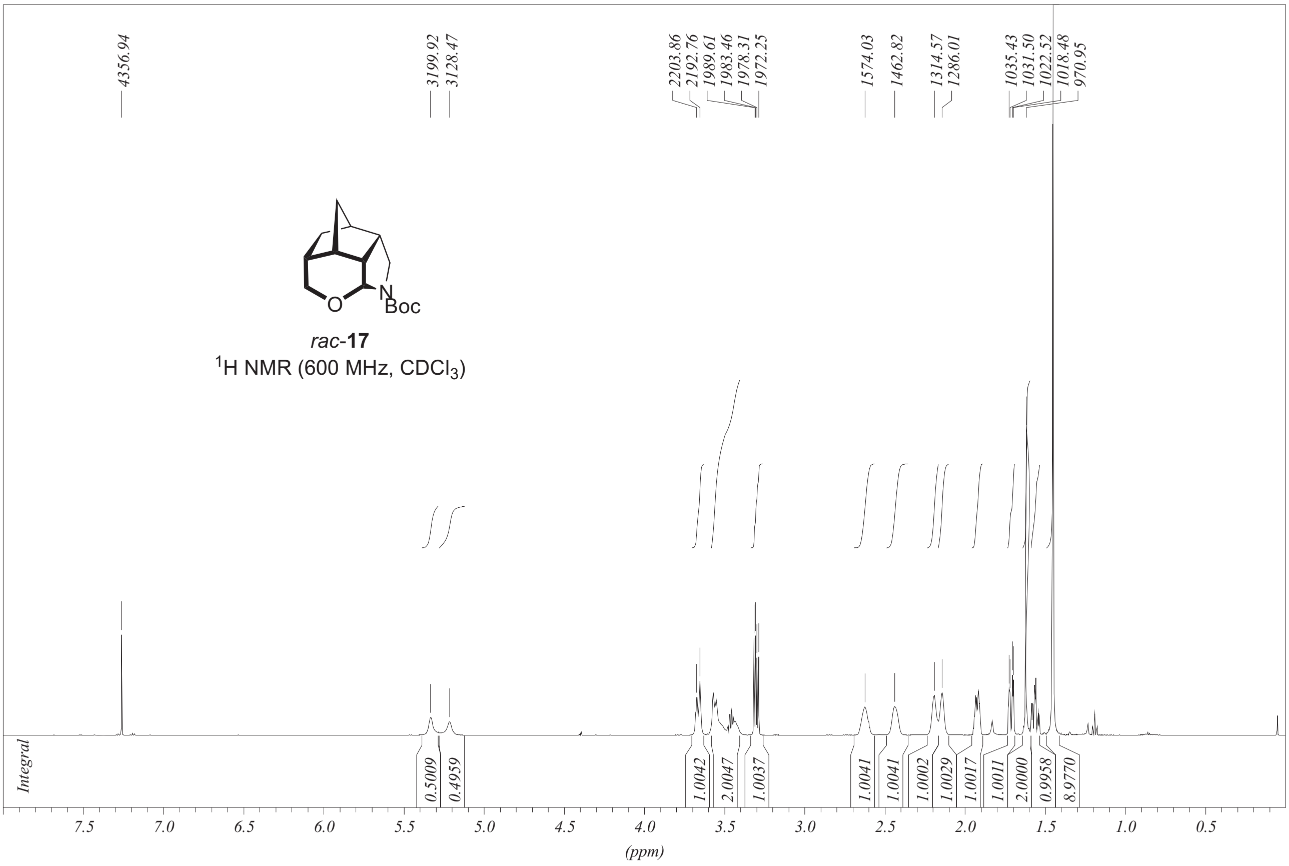
4356.94
3199.92
3128.47
2203.86
2192.76
1989.61
1983.46
1978.31
1972.25
1574.03
1462.82
1314.57
1286.01
1035.43
1031.50
1022.52
1018.48
970.95
O
N
Boc
rac-17
¹H NMR (600 MHz, CDCl3)
Integral
0.5009
0.4959
1.0042
2.0047
1.0037
1.0041
1.0041
1.0002
1.0029
1.0017
1.0011
2.0000
0.9958
8.9770
7.5
7.0
6.5
6.0
5.5
5.0
4.5
4.0
3.5
3.0
2.5
2.0
1.5
1.0
0.5
(ppm)

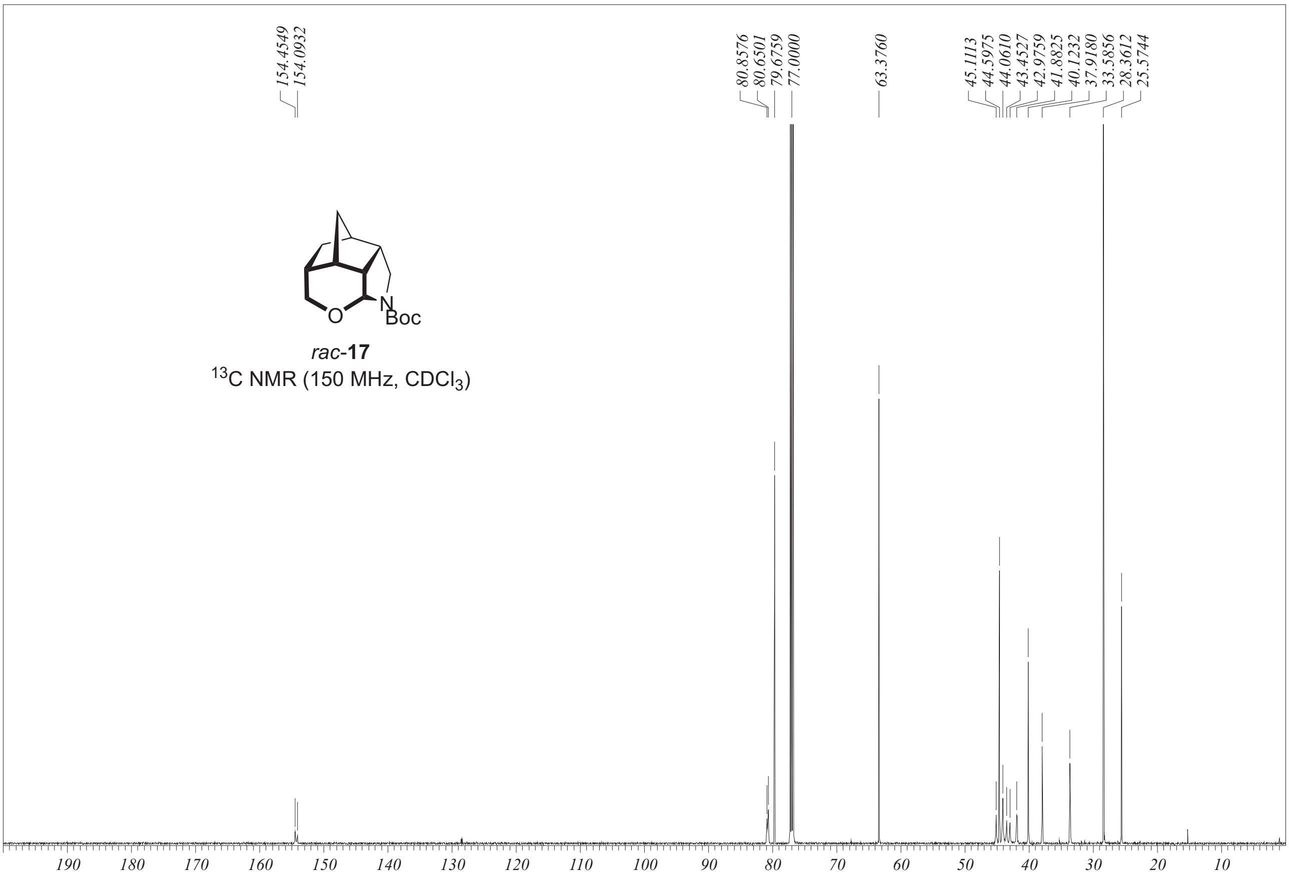
154.4549
154.0932
80.8576
80.6501
79.6759
77.0000
63.3760
45.1113
44.5975
44.0610
43.4527
42.9759
41.8825
40.1232
37.9180
33.5856
28.3612
25.5744
O
N
Boc
rac-17
¹³C NMR (150 MHz, CDCl3)
190
180
170
160
150
140
130
120
110
100
90
80
70
60
50
40
30
20
10
(ppm)

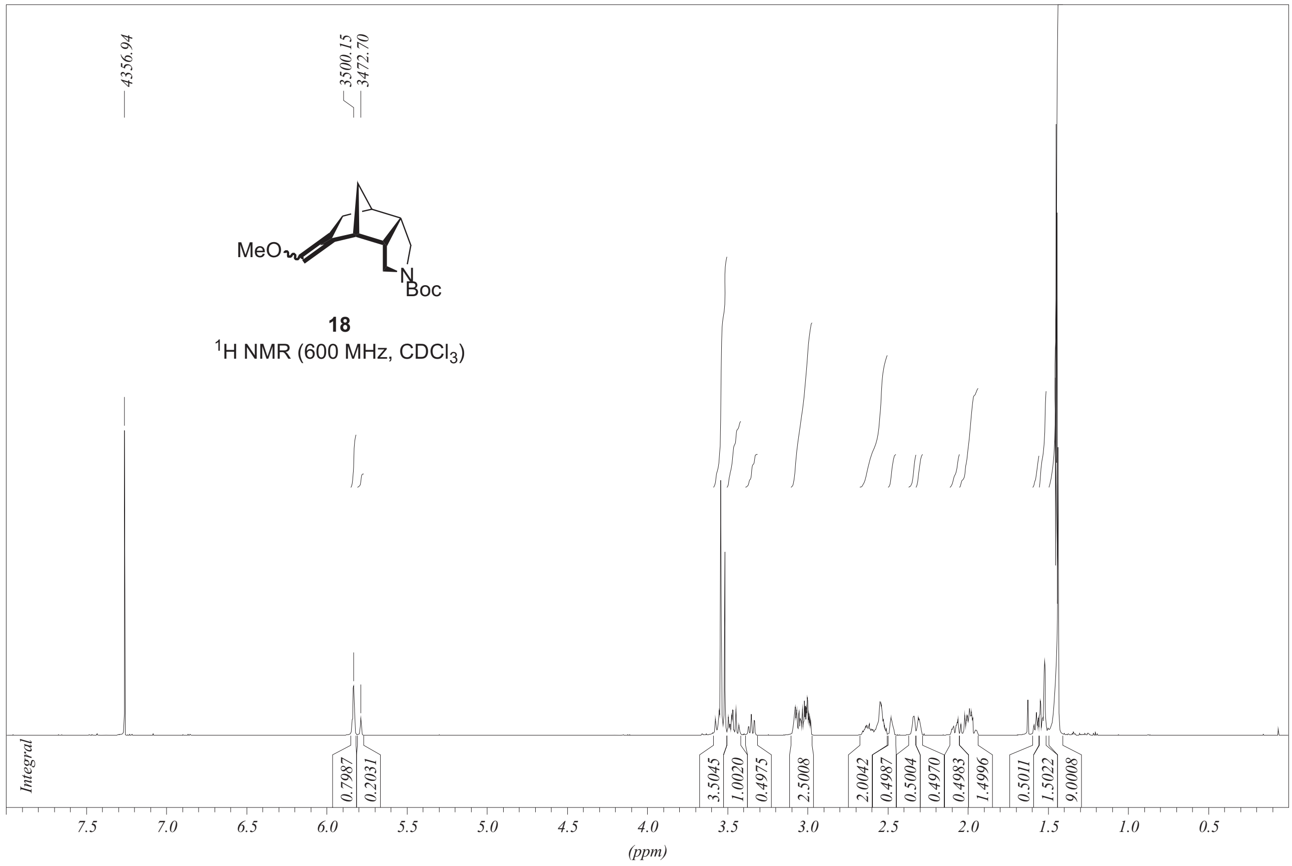
4356.94
3500.15
3472.70
MeO
N
Boc
18
1H NMR (600 MHz, CDCl3)
Integral
0.7987
0.2031
3.5045
1.0020
0.4975
2.5008
2.0042
0.4987
0.5004
0.4970
0.4983
1.4996
0.5011
1.5022
9.0008
7.5 7.0 6.5 6.0 5.5 5.0 4.5 4.0 3.5 3.0 2.5 2.0 1.5 1.0 0.5
(ppm)

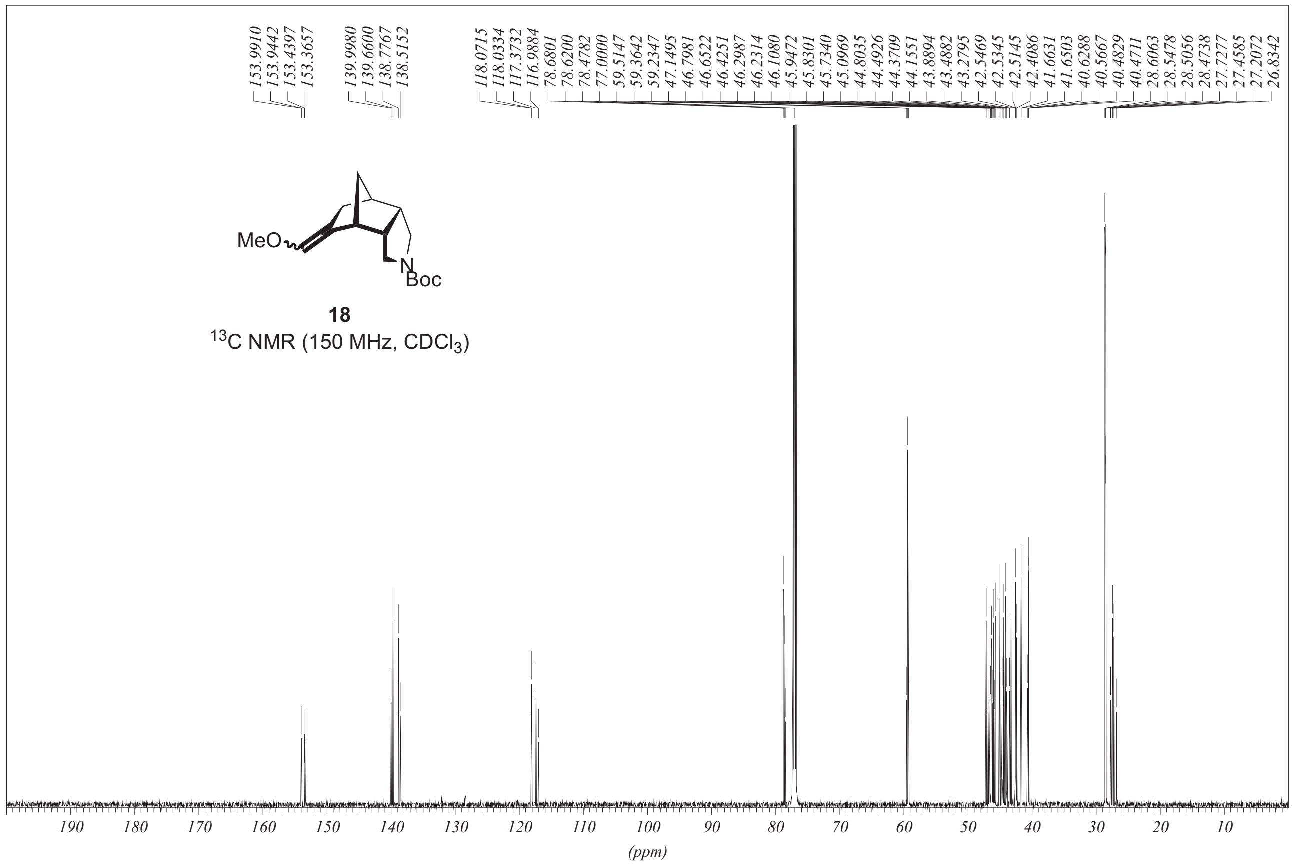
153.9910
153.9442
153.4397
153.3657
139.9980
139.6600
138.7767
138.5152
118.0715
118.0334
117.3732
116.9884
78.6801
78.6200
78.4782
77.0000
59.5147
59.3642
59.2347
47.1495
46.7981
46.6522
46.4251
46.2987
46.2314
46.1080
45.9472
45.8301
45.7340
45.0969
44.8035
44.4926
44.3709
44.1551
43.8894
43.4882
43.2795
42.5469
42.5345
42.5145
42.4086
41.6631
41.6503
40.6288
40.5667
40.4829
40.4711
28.6063
28.5478
28.5056
28.4738
27.7277
27.4585
27.2072
26.8342
MeO
N
Boc
18
13C NMR (150 MHz, CDCl3)
190 180 170 160 150 140 130 120 110 100 90 80 70 60 50 40 30 20 10
(ppm)

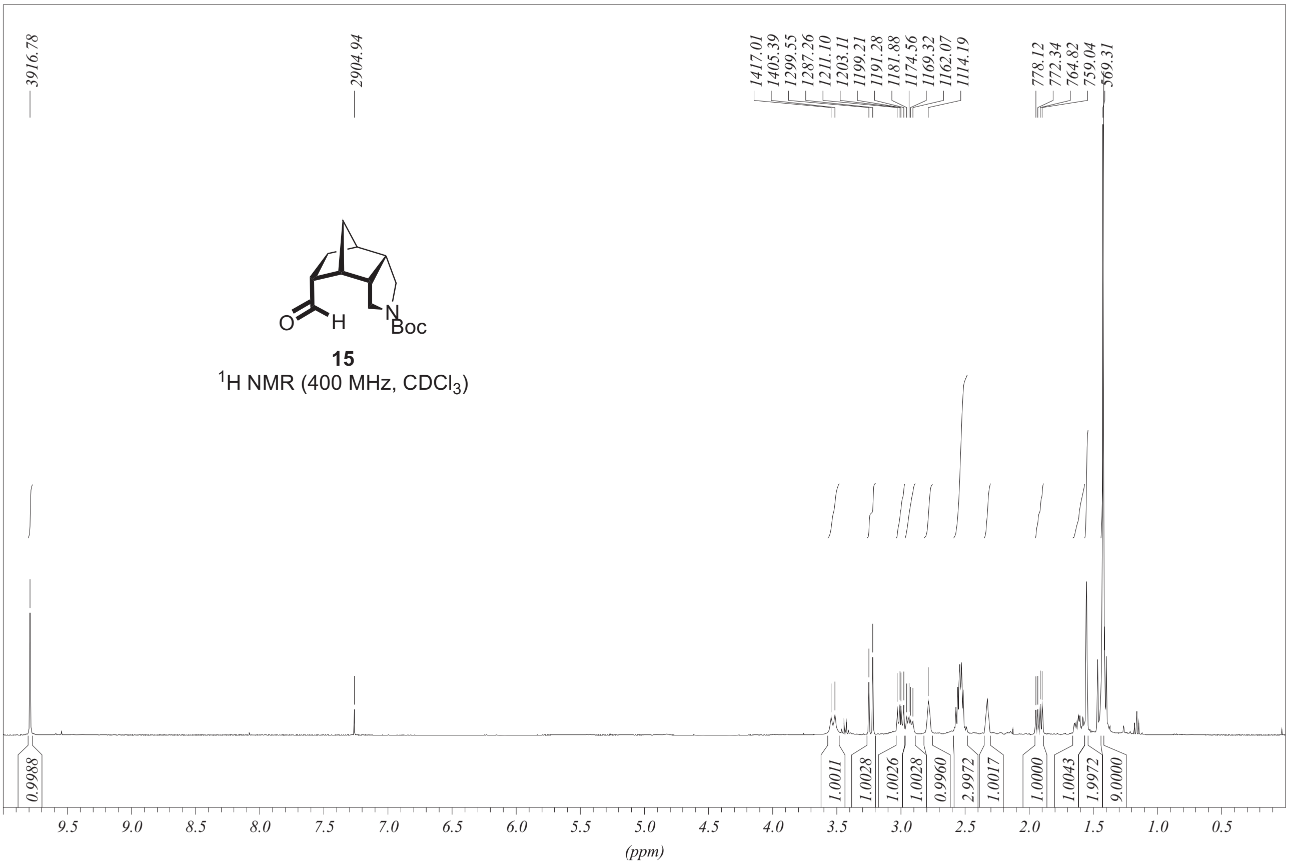

3916.78
2904.94
1417.01
1405.39
1299.55
1287.26
1211.10
1203.11
1199.21
1191.28
1181.88
1174.56
1169.32
1162.07
1114.19
778.12
772.34
764.82
759.04
569.31
O
H
N
Boc
15
¹H NMR (400 MHz, CDCl3)
0.9988
1.0011
1.0028
1.0026
1.0028
0.9960
2.9972
1.0017
1.0000
1.0043
1.9972
9.0000
9.5
9.0
8.5
8.0
7.5
7.0
6.5
6.0
5.5
5.0
4.5
4.0
3.5
3.0
2.5
2.0
1.5
1.0
0.5
(ppm)

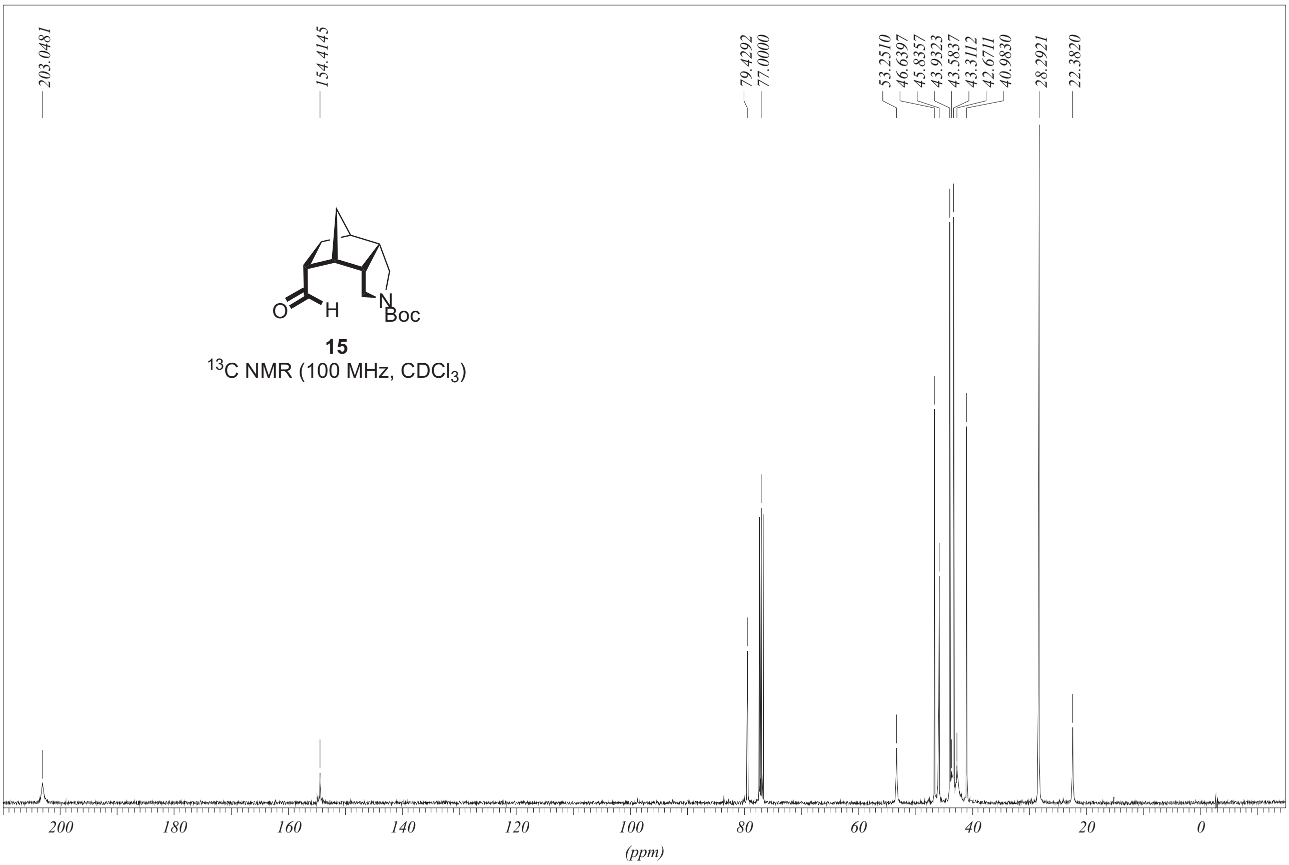

203.0481
154.4145
79.4292
77.0000
53.2510
46.6397
45.8357
43.9323
43.5837
43.3112
42.6711
40.9830
28.2921
22.3820
O
H
N
Boc
15
¹³C NMR (100 MHz, CDCl3)
200
180
160
140
120
100
80
60
40
20
0
(ppm)

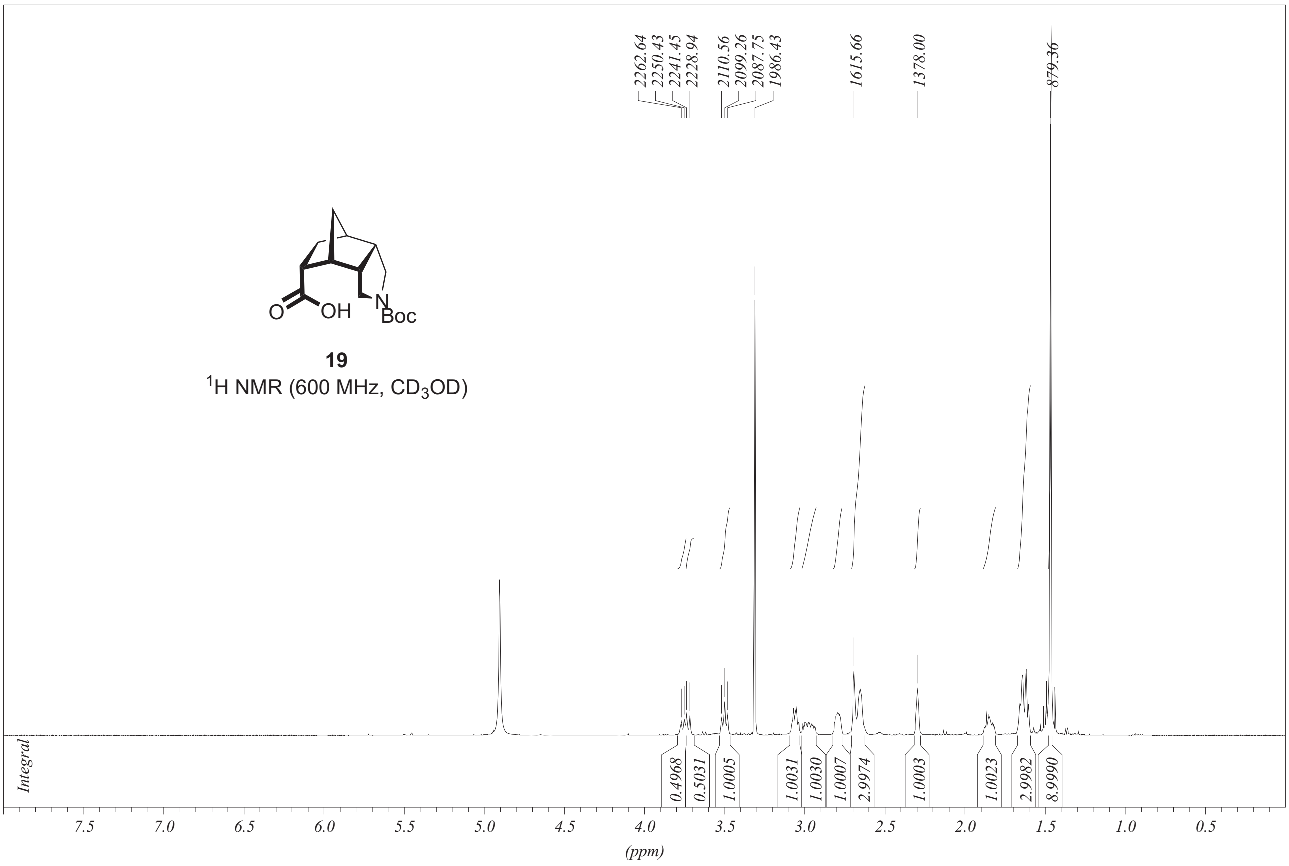
2262.64
2250.43
2241.45
2228.94
2110.56
2099.26
2087.75
1986.43
1615.66
1378.00
879.36
OH
O
N
Boc
19
^{1}H NMR (600 MHz, CD_3OD)
Integral
0.4968
0.5031
1.0005
1.0031
1.0030
1.0007
2.9974
1.0003
1.0023
2.9982
8.9990
7.5
7.0
6.5
6.0
5.5
5.0
4.5
4.0
3.5
3.0
2.5
2.0
1.5
1.0
0.5
(ppm)

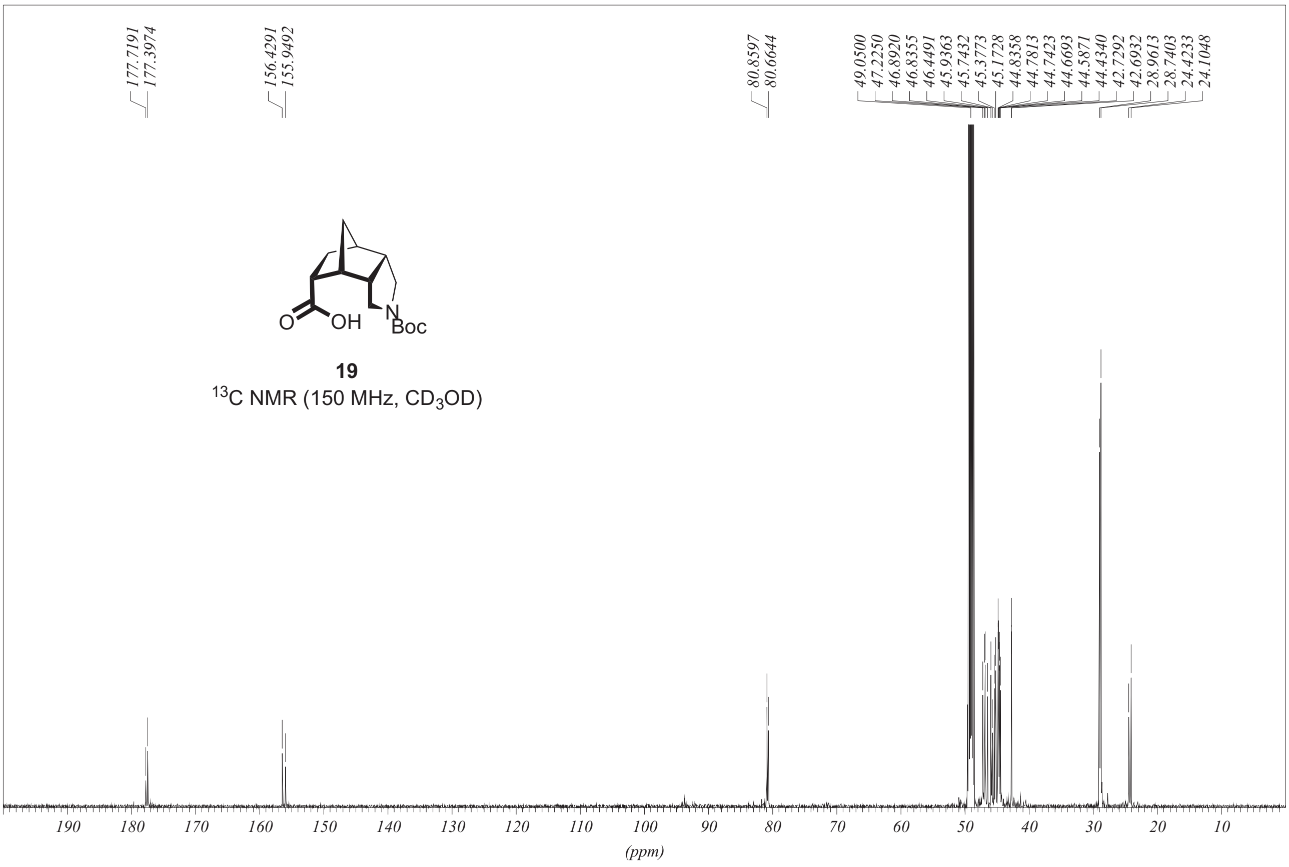
177.7191
177.3974
156.4291
155.9492
80.8597
80.6644
49.0500
47.2250
46.8920
46.8355
46.4491
45.9363
45.7432
45.3773
45.1728
44.8358
44.7813
44.7423
44.6693
44.5871
44.4340
42.7292
42.6932
28.9613
28.7403
24.4233
24.1048
OH
O
N
Boc
19
^{13}C NMR (150 MHz, CD_3OD)
190
180
170
160
150
140
130
120
110
100
90
80
70
60
50
40
30
20
10
(ppm)

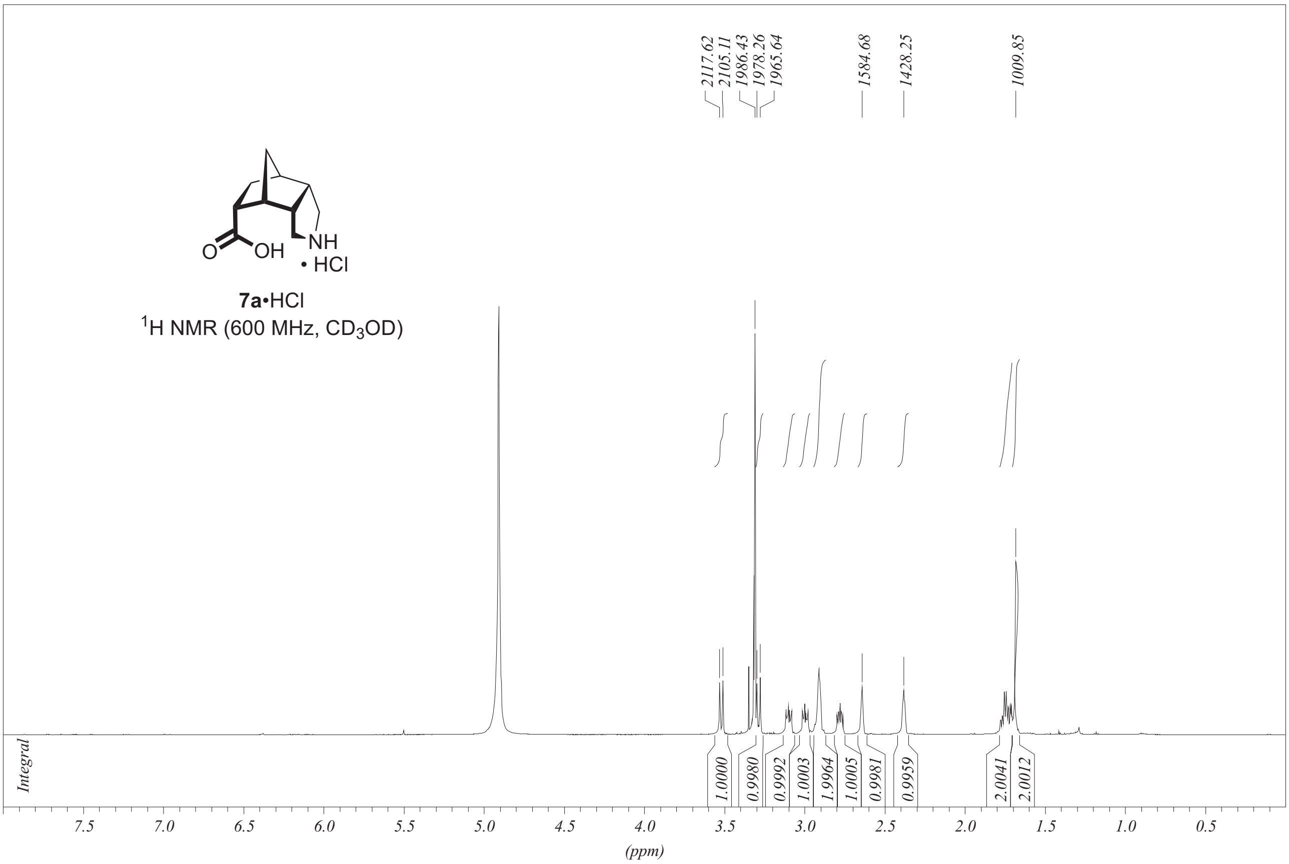
2117.62
2105.11
1986.43
1978.26
1965.64
1584.68
1428.25
1009.85
NH
O
OH
• HCl
7a•HCl
^{1}H NMR (600 MHz, CD_3OD)
Integral
1.0000
0.9980
0.9992
1.0003
1.9964
1.0005
0.9981
0.9959
2.0041
2.0012
7.5 7.0 6.5 6.0 5.5 5.0 4.5 4.0 3.5 3.0 2.5 2.0 1.5 1.0 0.5
(ppm)

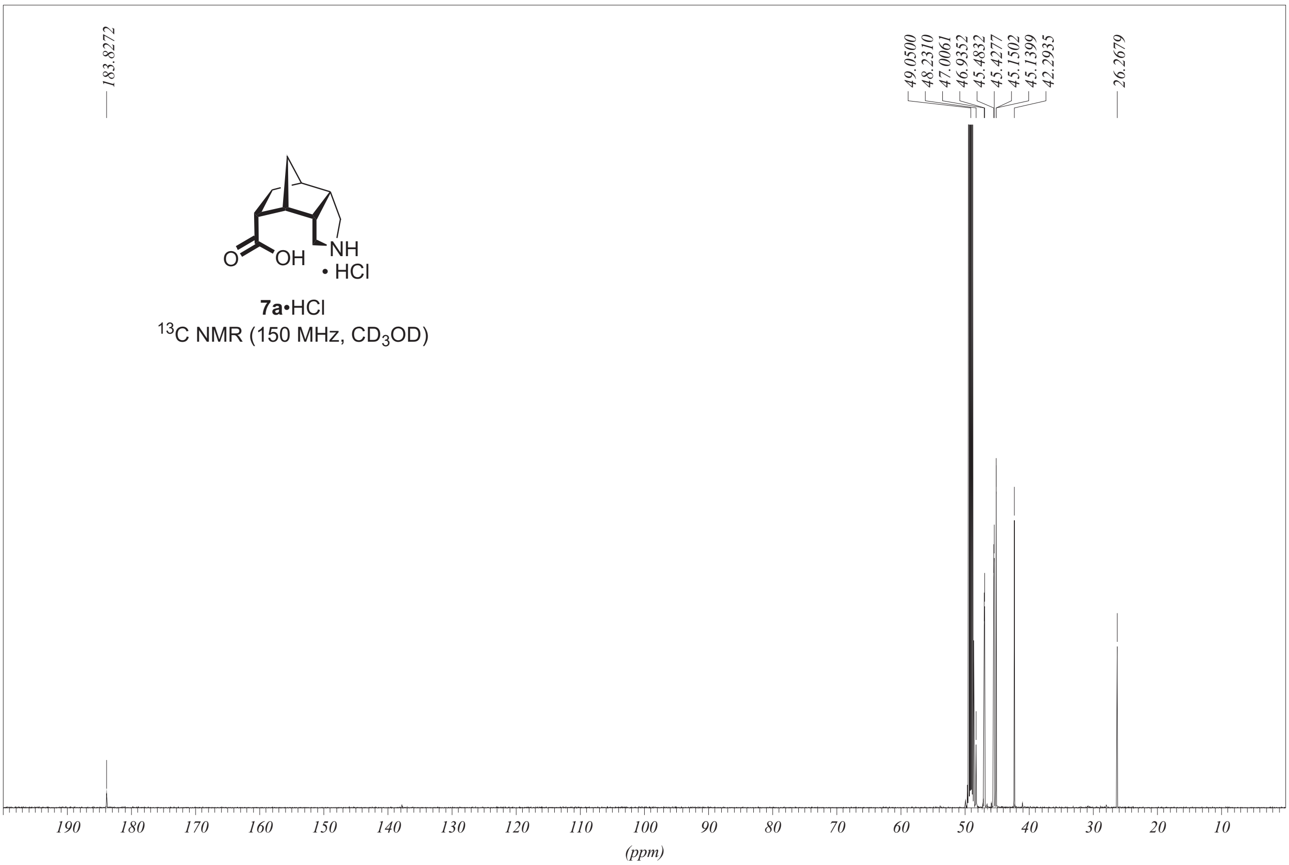
183.8272
49.0500
48.2310
47.0061
46.9352
45.4832
45.4277
45.1502
45.1399
42.2935
26.2679
NH
O
OH
• HCl
7a•HCl
^{13}C NMR (150 MHz, CD_3OD)
190 180 170 160 150 140 130 120 110 100 90 80 70 60 50 40 30 20 10
(ppm)

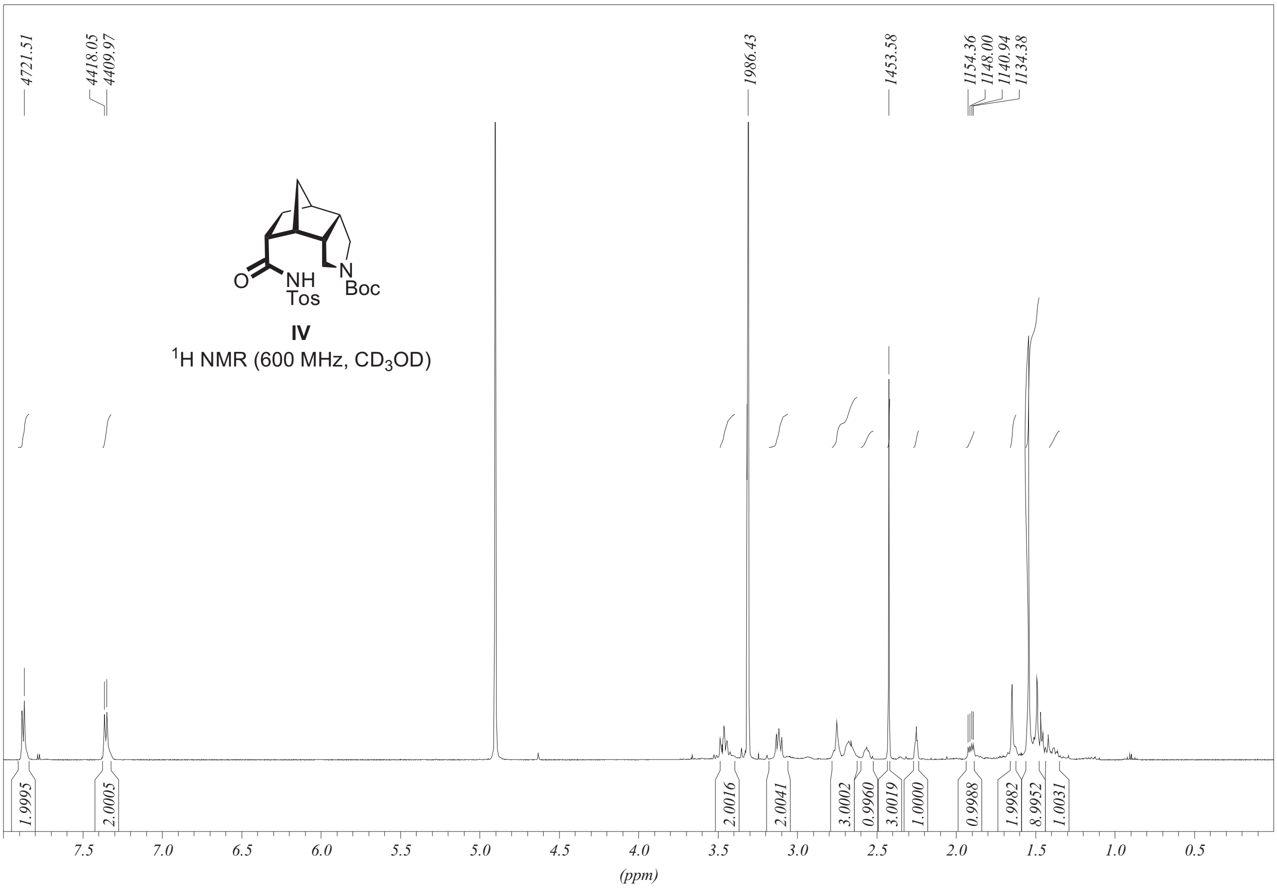
4721.51
4418.05
4409.97
1986.43
1453.58
1154.36
1148.00
1140.94
1134.38
NH
O
N
Boc
Tos
IV
^{1}H NMR (600 MHz, CD_3OD)
1.9995
2.0005
2.0016
2.0041
3.0002
0.9960
3.0019
1.0000
0.9988
1.9982
8.9952
1.0031
7.5
7.0
6.5
6.0
5.5
5.0
4.5
4.0
3.5
3.0
2.5
2.0
1.5
1.0
0.5
(ppm)

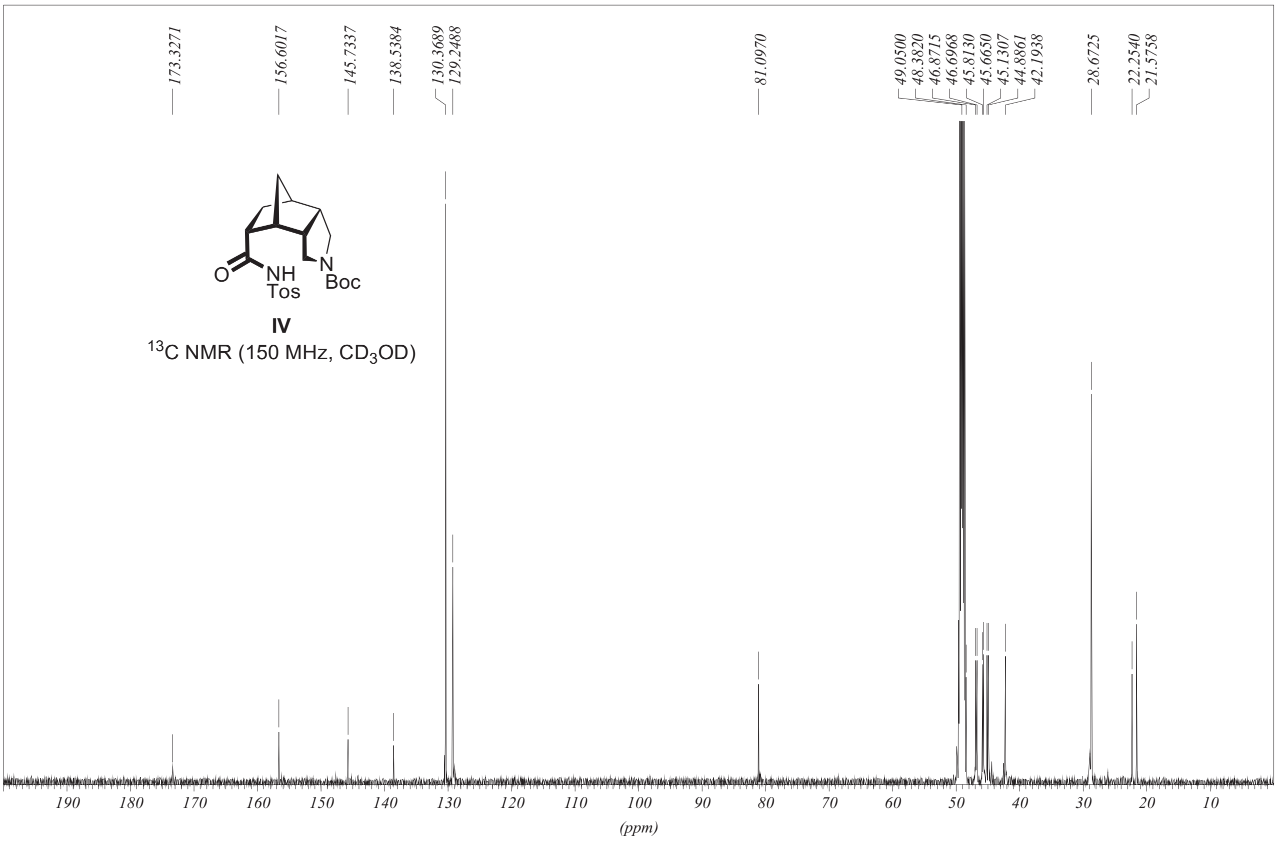
173.3271
156.6017
145.7337
138.5384
130.3689
129.2488
81.0970
49.0500
48.3820
46.8715
46.6968
45.8130
45.6650
45.1307
44.8861
42.1938
28.6725
22.2540
21.5758
NH
O
N
Boc
Tos
IV
^{13}C NMR (150 MHz, CD_3OD)
190
180
170
160
150
140
130
120
110
100
90
80
70
60
50
40
30
20
10
(ppm)

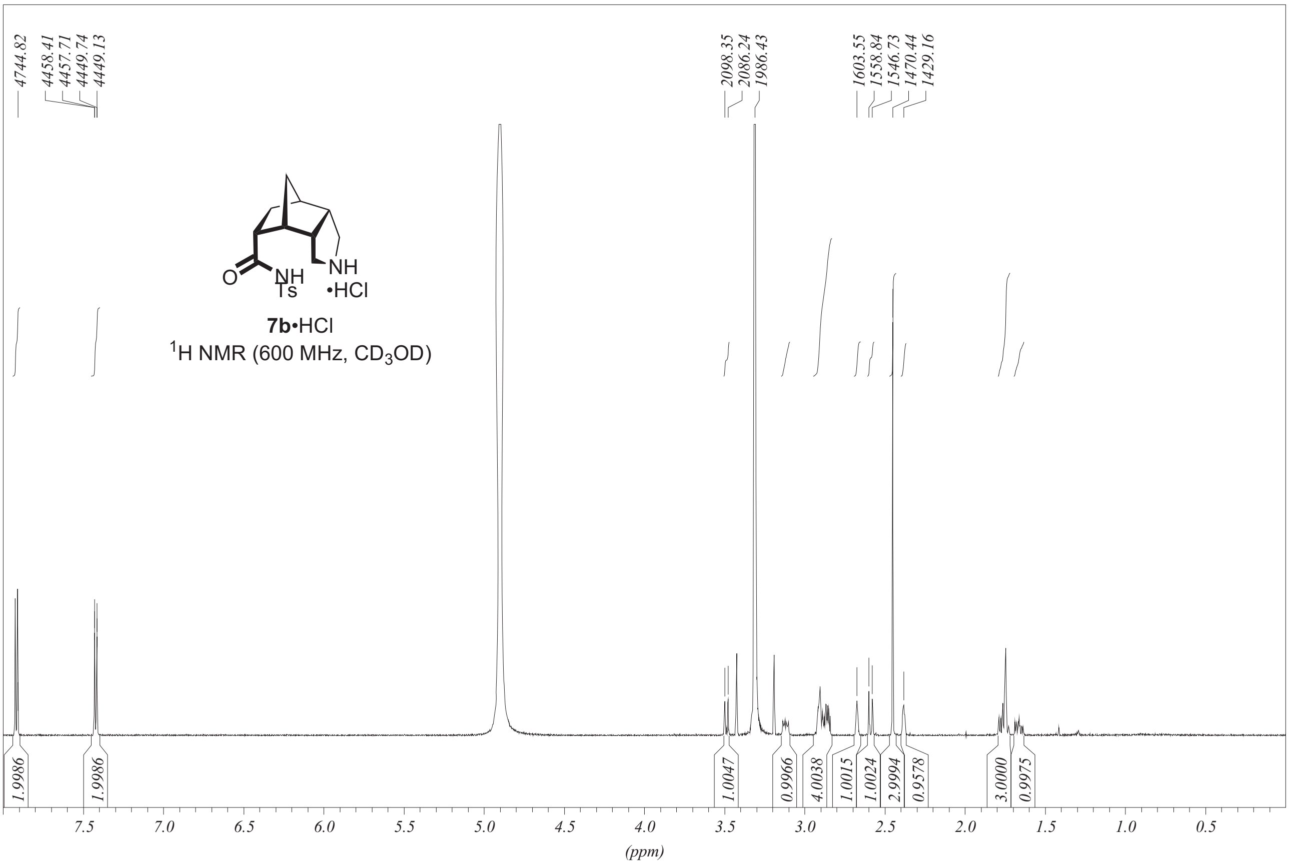
4744.82
4458.41
4457.71
4449.74
4449.13
2098.35
2086.24
1986.43
1603.55
1558.84
1546.73
1470.44
1429.16
NH
O
NH
Ts
•HCl
7b•HCl
1H NMR (600 MHz, CD3OD)
1.9986
1.9986
1.0047
0.9966
4.0038
1.0015
1.0024
2.9994
0.9578
3.0000
0.9975
(ppm)

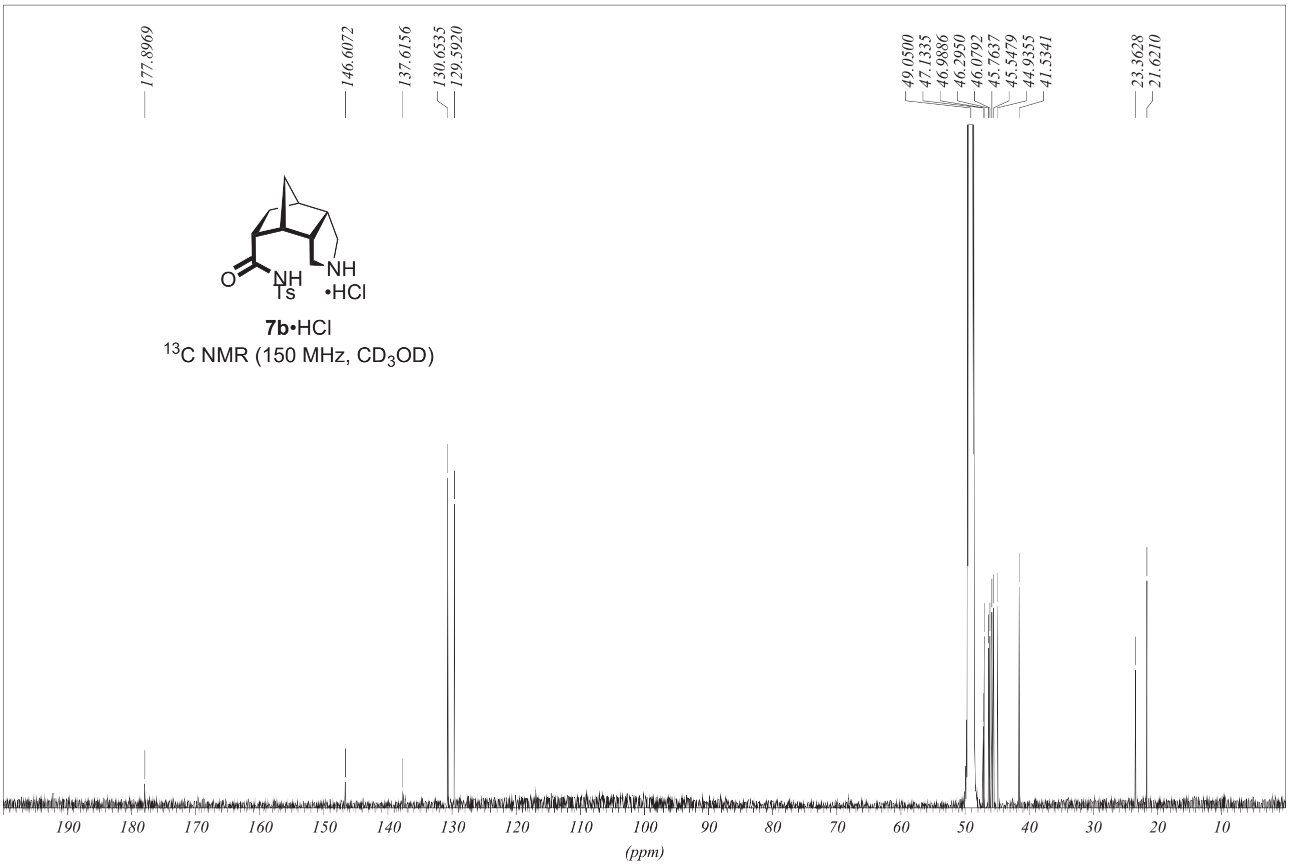
177.8969
146.6072
137.6156
130.6535
129.5920
49.0500
47.1335
46.9886
46.2950
46.0792
45.7637
45.5479
44.9355
41.5341
23.3628
21.6210
NH
O
NH
Ts
•HCl
7b•HCl
13C NMR (150 MHz, CD3OD)
(ppm)

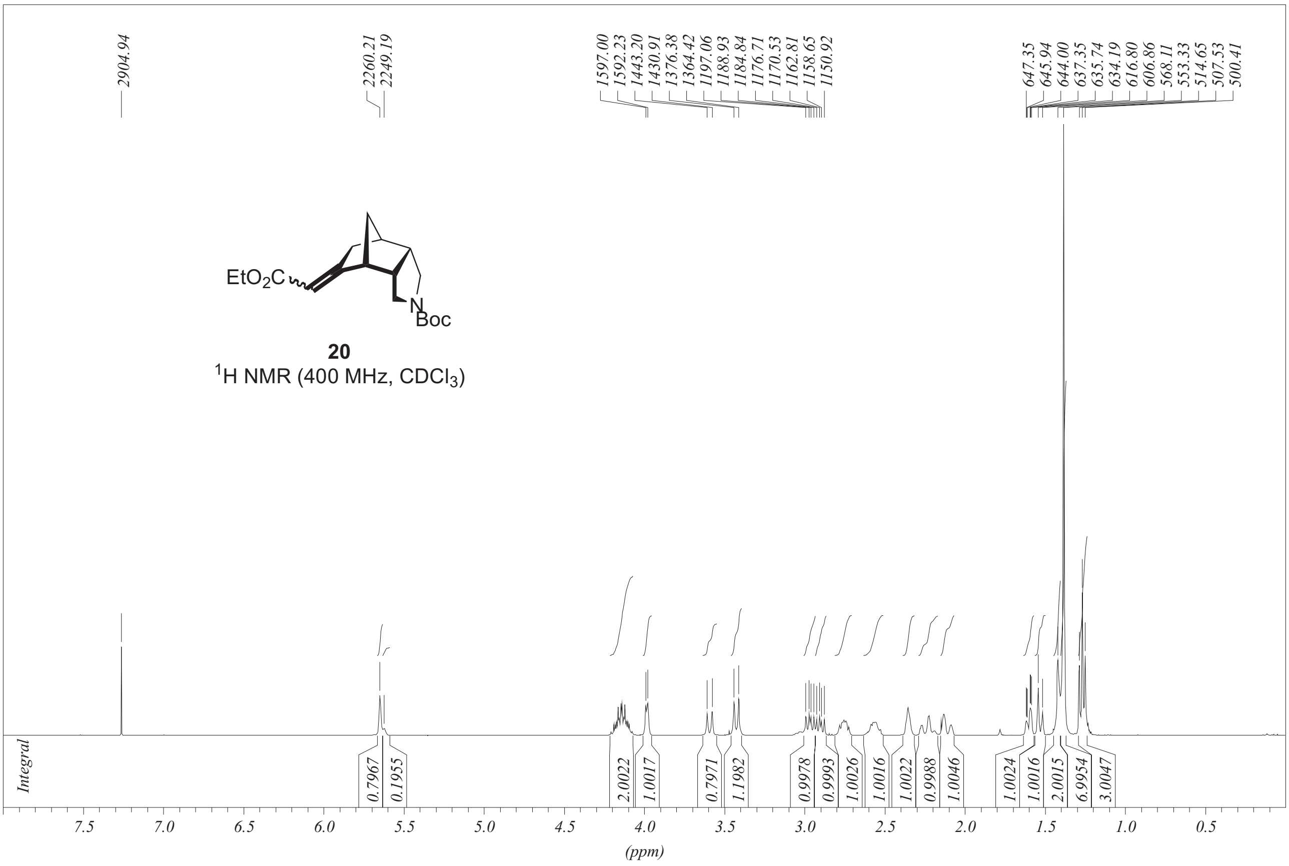

20

^{1}H NMR (400 MHz, $CDCl_3$)

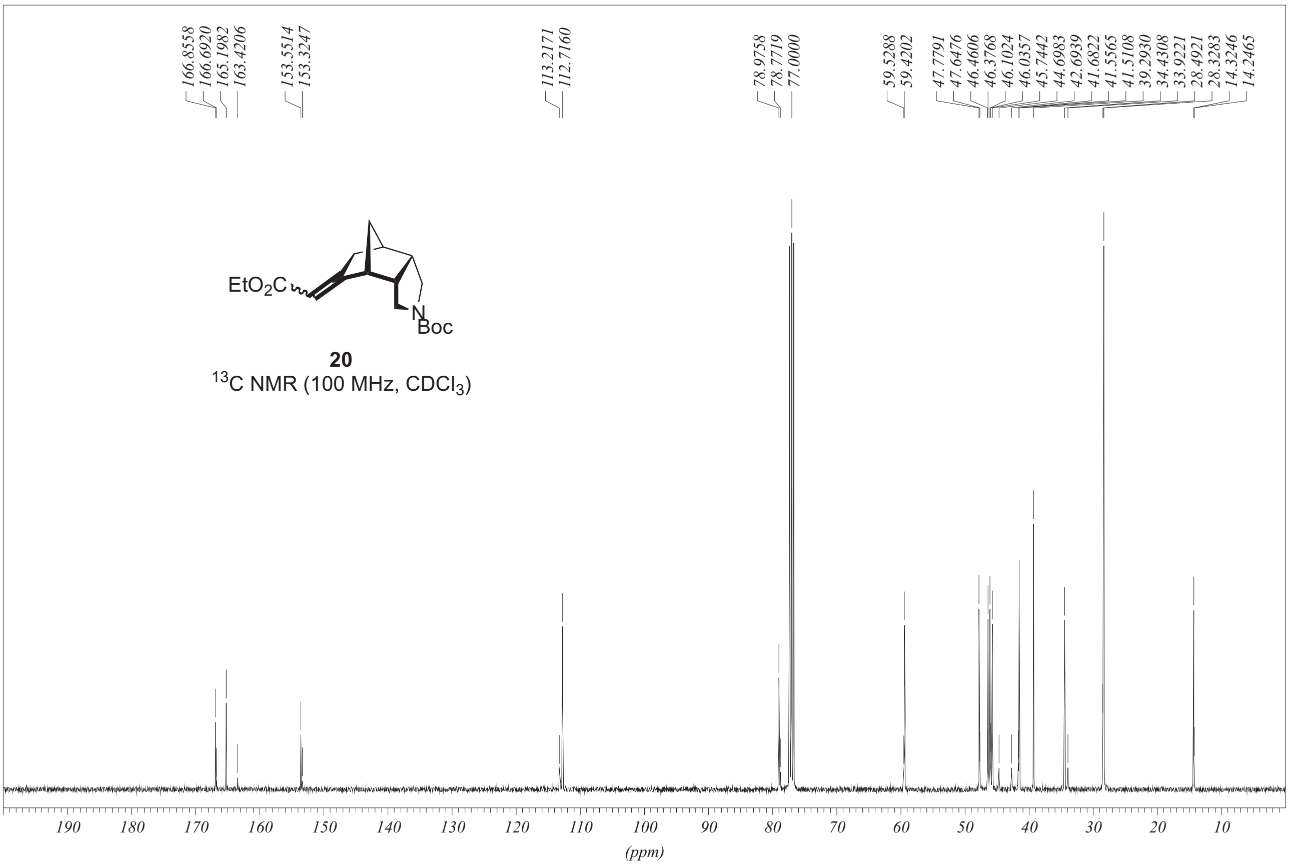

20

^{13}C NMR (100 MHz, $CDCl_3$)

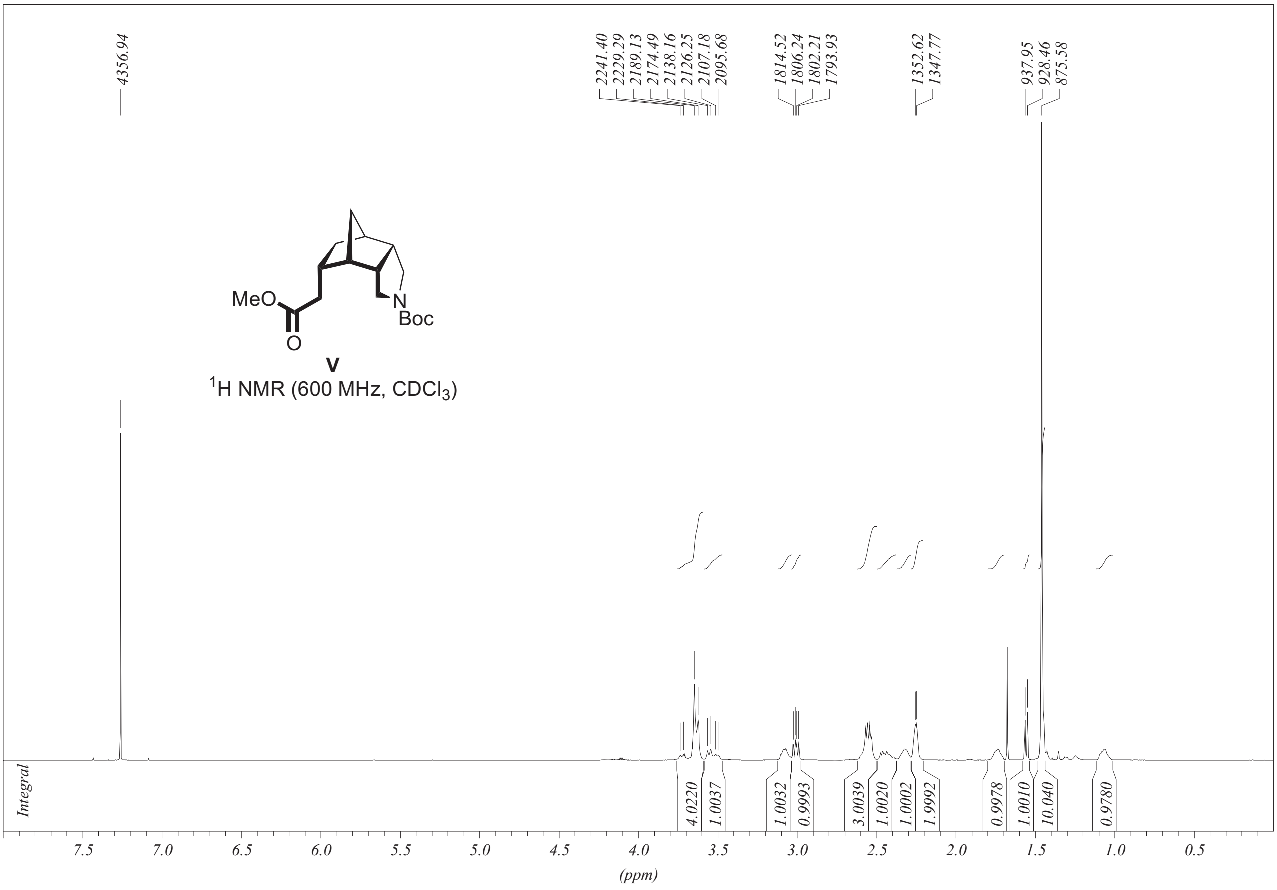
4356.94
2241.40
2229.29
2189.13
2174.49
2138.16
2126.25
2107.18
2095.68
1814.52
1806.24
1802.21
1793.93
1352.62
1347.77
937.95
928.46
875.58
MeO
O
N
Boc
V
^{1}H NMR (600 MHz, CDCl$_3$)
Integral
4.0220
1.0037
1.0032
0.9993
3.0039
1.0020
1.0002
1.9992
0.9978
1.0010
10.040
0.9780
7.5 7.0 6.5 6.0 5.5 5.0 4.5 4.0 3.5 3.0 2.5 2.0 1.5 1.0 0.5
(ppm)

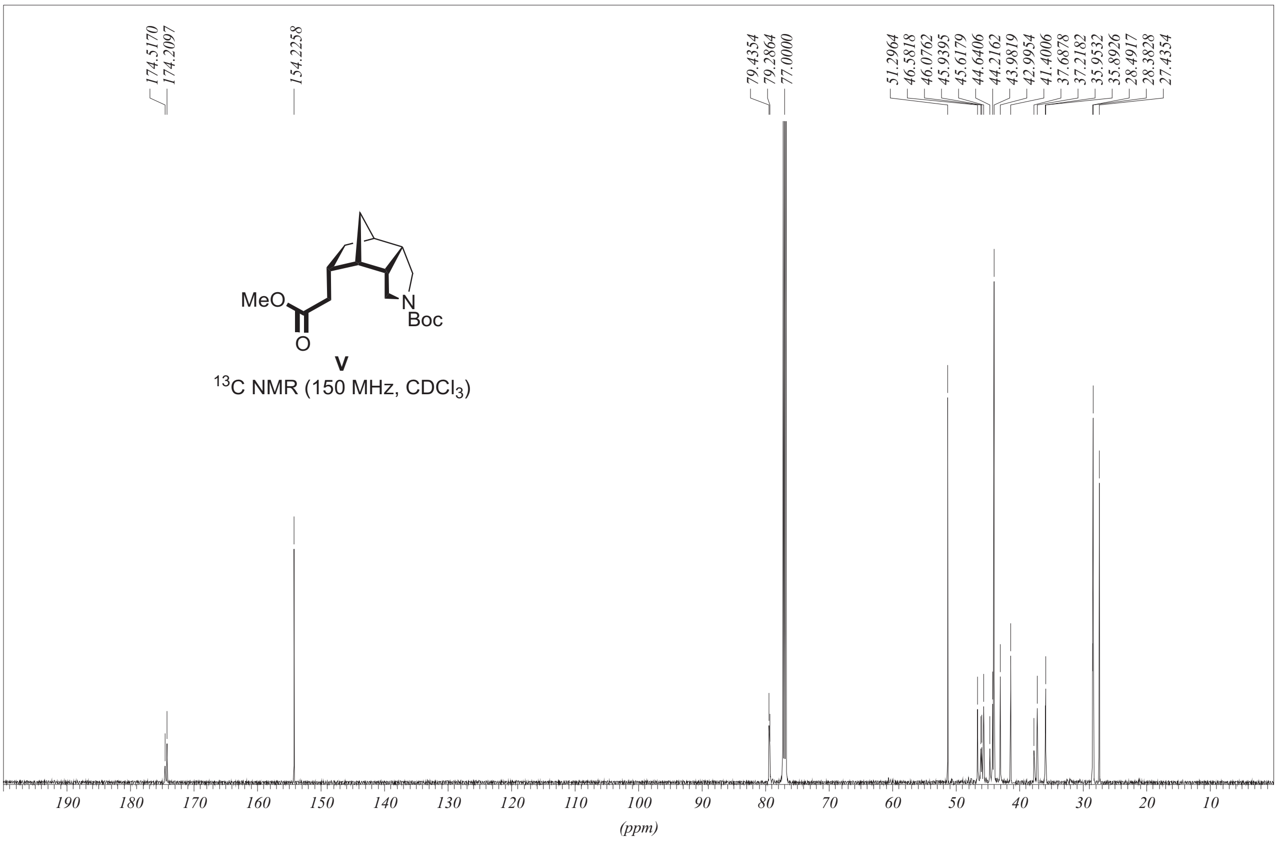
174.5170
174.2097
154.2258
79.4354
79.2864
77.0000
51.2964
46.5818
46.0762
45.9395
45.6179
44.6406
44.2162
43.9819
42.9954
41.4006
37.6878
37.2182
35.9532
35.8926
28.4917
28.3828
27.4354
MeO
O
N
Boc
V
^{13}C NMR (150 MHz, CDCl$_3$)
190 180 170 160 150 140 130 120 110 100 90 80 70 60 50 40 30 20 10
(ppm)

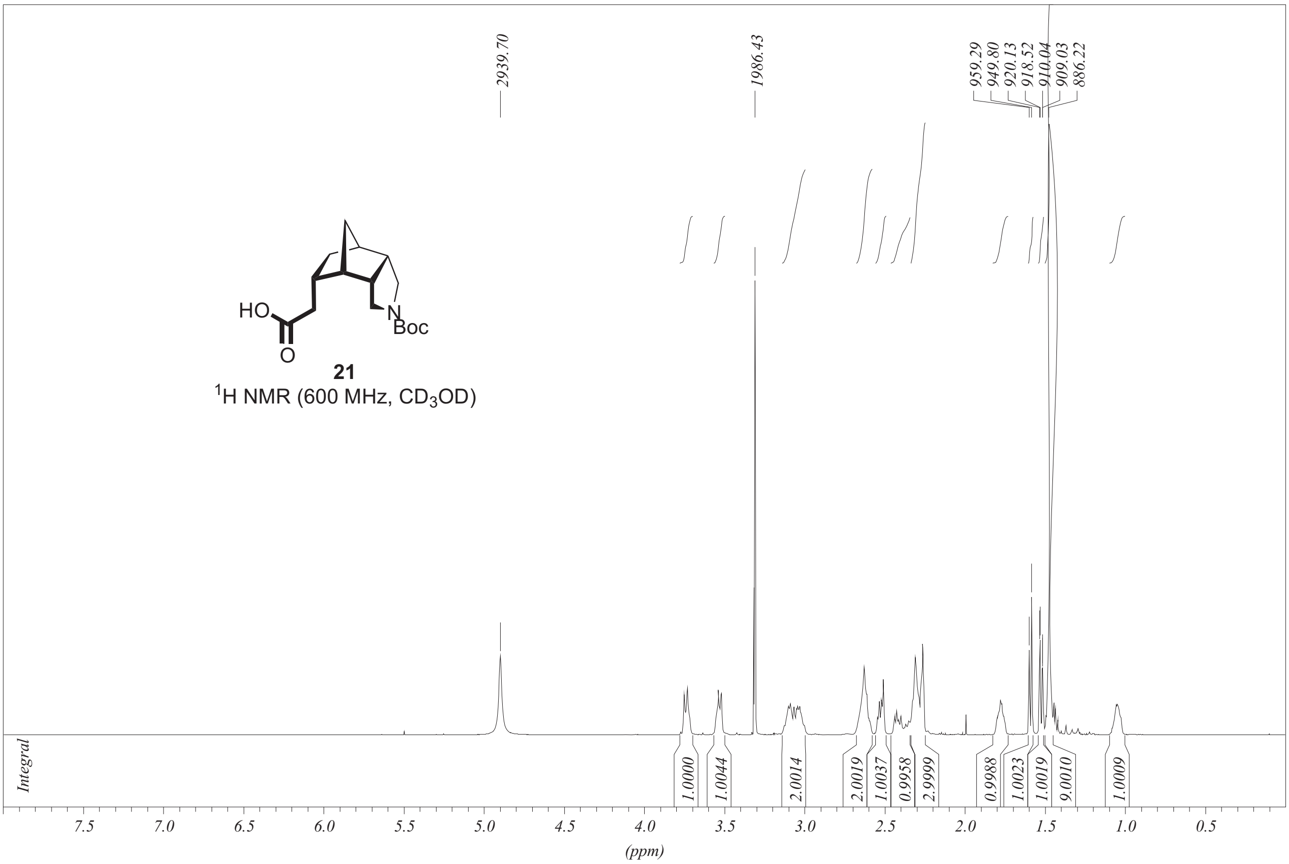
2939.70
1986.43
959.29
949.80
920.13
918.52
910.04
909.03
886.22
HO
O
N
Boc
21
^{1}H NMR (600 MHz, CD_3OD)
Integral
1.0000
1.0044
2.0014
2.0019
1.0037
0.9958
2.9999
0.9988
1.0023
1.0019
9.0010
1.0009
7.5 7.0 6.5 6.0 5.5 5.0 4.5 4.0 3.5 3.0 2.5 2.0 1.5 1.0 0.5
(ppm)

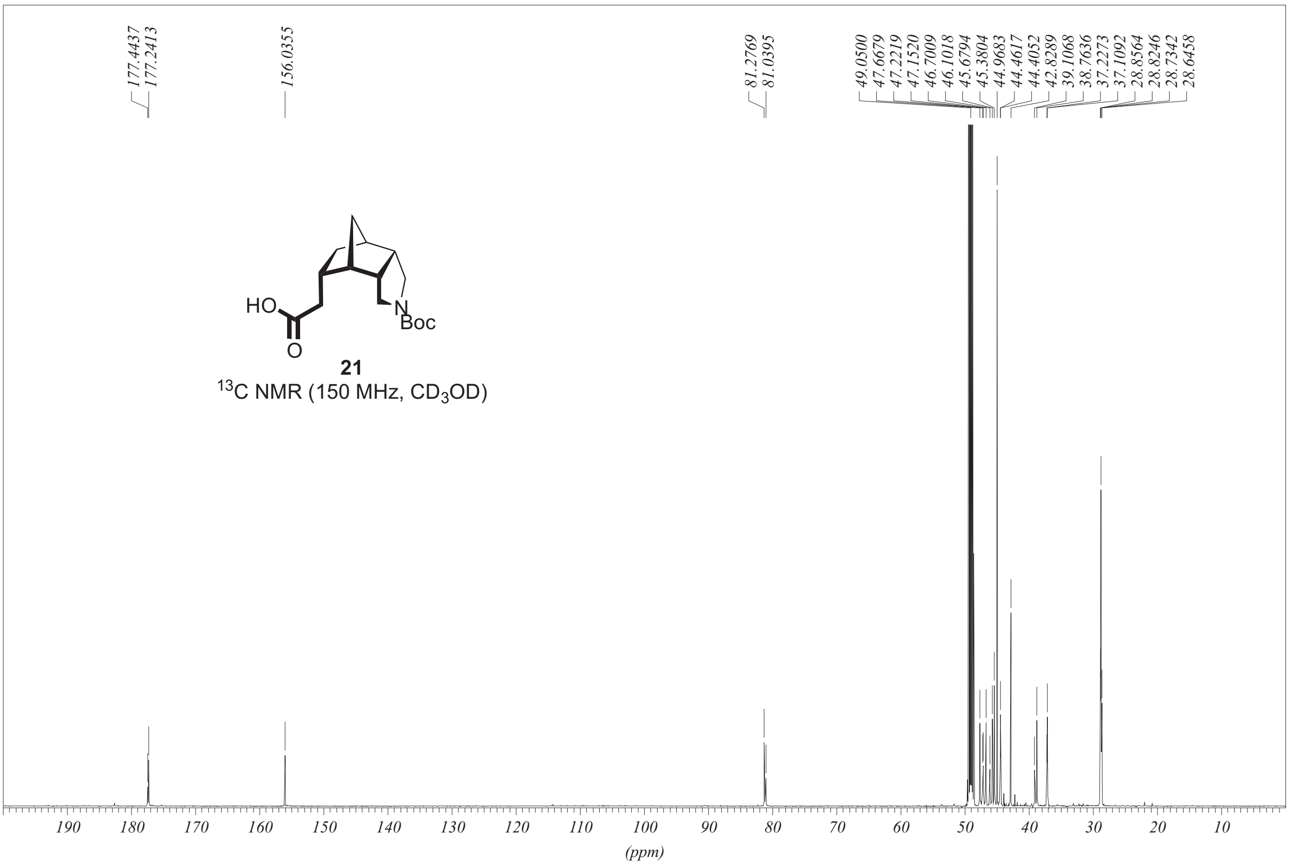
177.4437
177.2413
156.0355
81.2769
81.0395
49.0500
47.6679
47.2219
47.1520
46.7009
46.1018
45.6794
45.3804
44.9683
44.4617
44.4052
42.8289
39.1068
38.7636
37.2273
37.1092
28.8564
28.8246
28.7342
28.6458
HO
O
N
Boc
21
^{13}C NMR (150 MHz, CD_3OD)
190 180 170 160 150 140 130 120 110 100 90 80 70 60 50 40 30 20 10
(ppm)

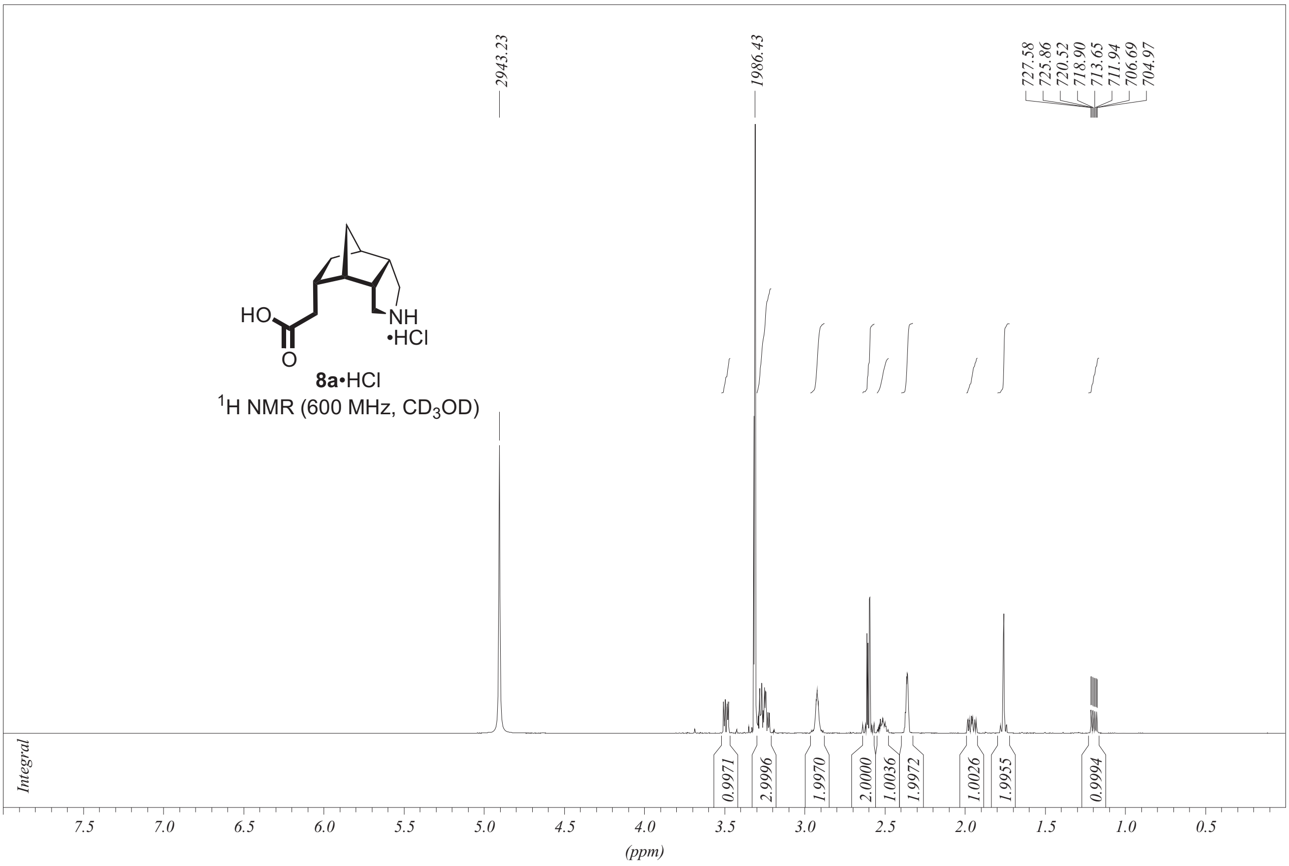

2943.23
1986.43
727.58
725.86
720.52
718.90
713.65
711.94
706.69
704.97
HO
NH
•HCl
O
8a•HCl
1H NMR (600 MHz, CD3OD)
Integral
0.9971
2.9996
1.9970
2.0000
1.0036
1.9972
1.0026
1.9955
0.9994
7.5
7.0
6.5
6.0
5.5
5.0
4.5
4.0
3.5
3.0
2.5
2.0
1.5
1.0
0.5
(ppm)

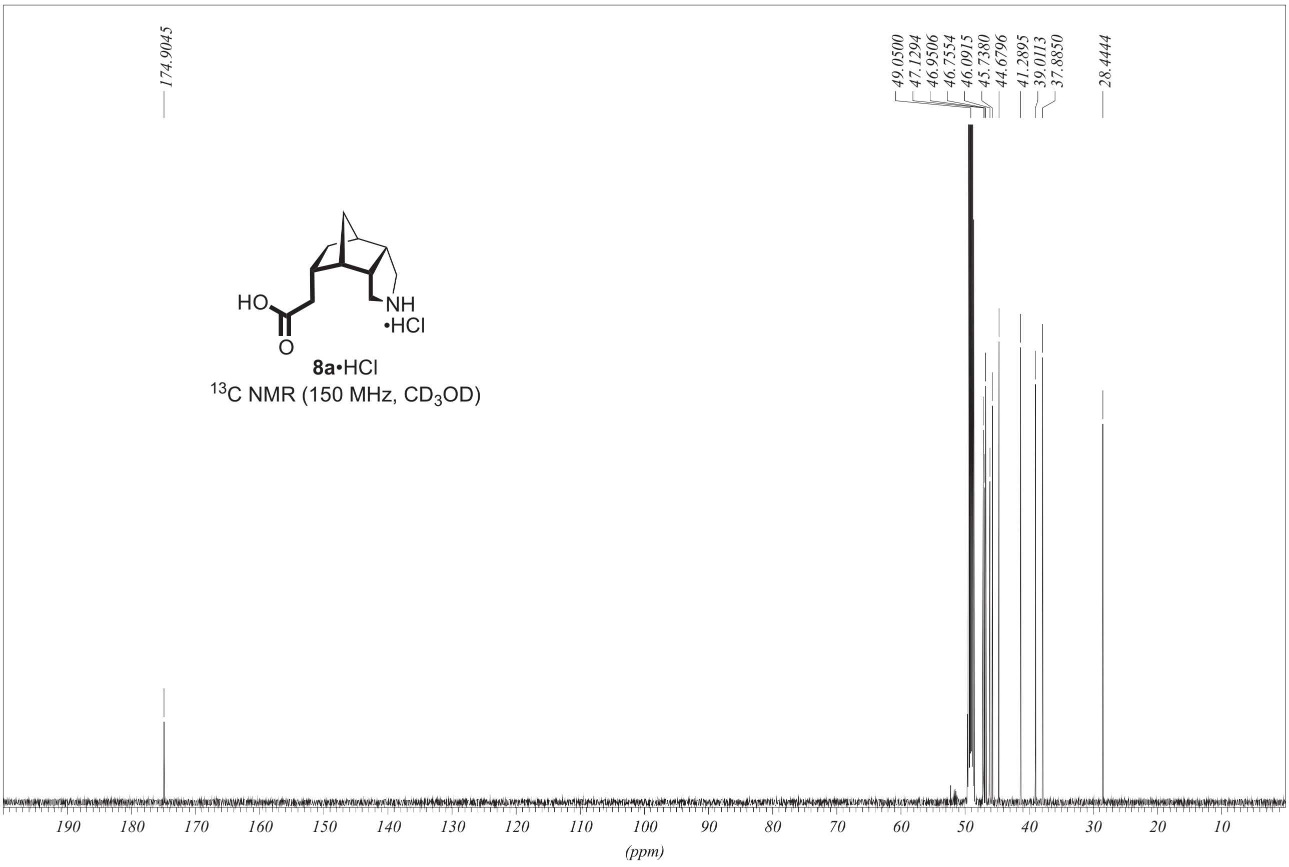

174.9045
49.0500
47.1294
46.9506
46.7554
46.0915
45.7380
44.6796
41.2895
39.0113
37.8850
28.4444
HO
NH
•HCl
O
8a•HCl
13C NMR (150 MHz, CD3OD)
190
180
170
160
150
140
130
120
110
100
90
80
70
60
50
40
30
20
10
(ppm)

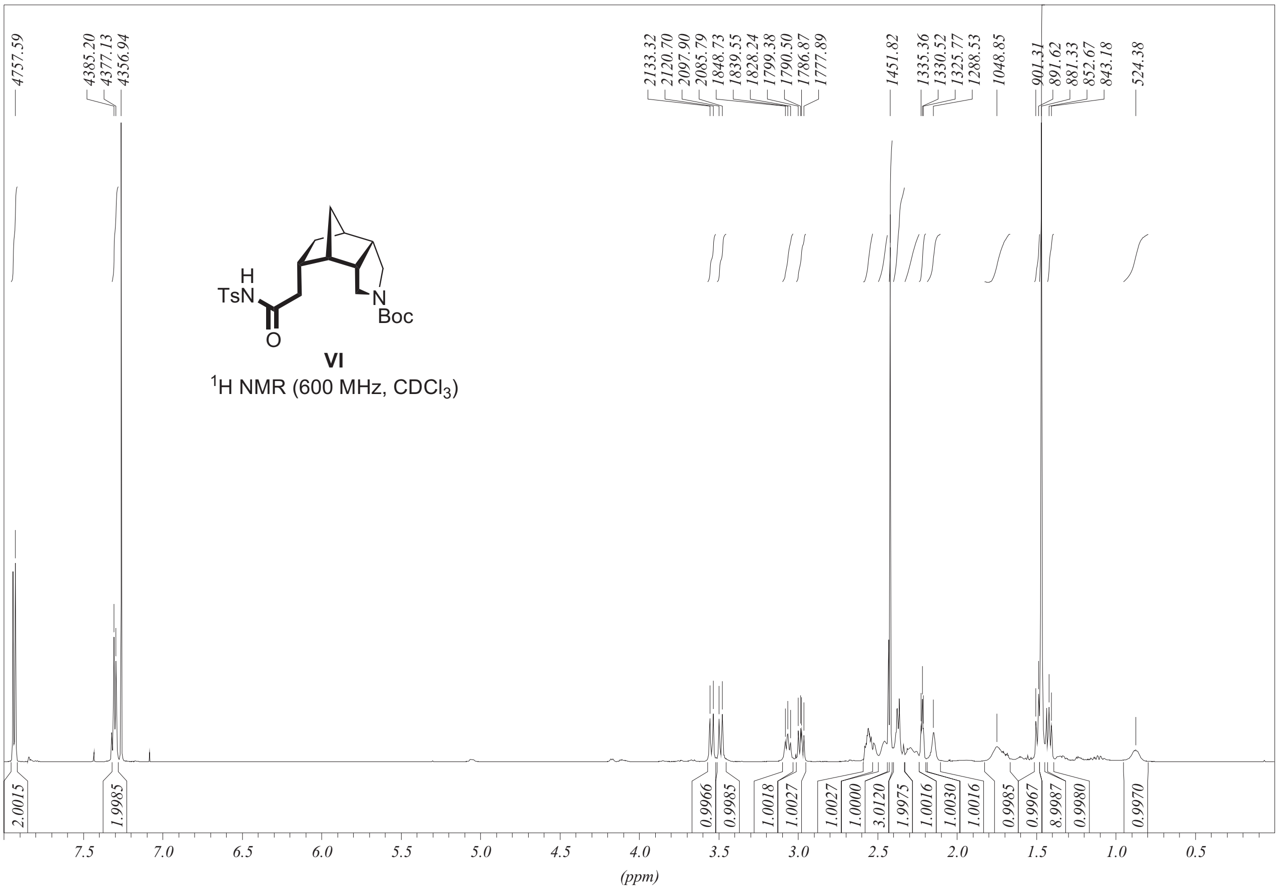
4757.59
4385.20
4377.13
4356.94
2133.32
2120.70
2097.90
2085.79
1848.73
1839.55
1828.24
1799.38
1790.50
1786.87
1777.89
1451.82
1335.36
1330.52
1325.77
1288.53
1048.85
901.31
891.62
881.33
852.67
843.18
524.38
H
TsN
N
Boc
O
VI
^{1}H NMR (600 MHz, $CDCl_3$)
2.0015
1.9985
0.9966
0.9985
1.0018
1.0027
1.0027
1.0000
3.0120
1.9975
1.0016
1.0030
1.0016
0.9985
0.9967
8.9987
0.9980
0.9970
7.5
7.0
6.5
6.0
5.5
5.0
4.5
4.0
3.5
3.0
2.5
2.0
1.5
1.0
0.5
(ppm)

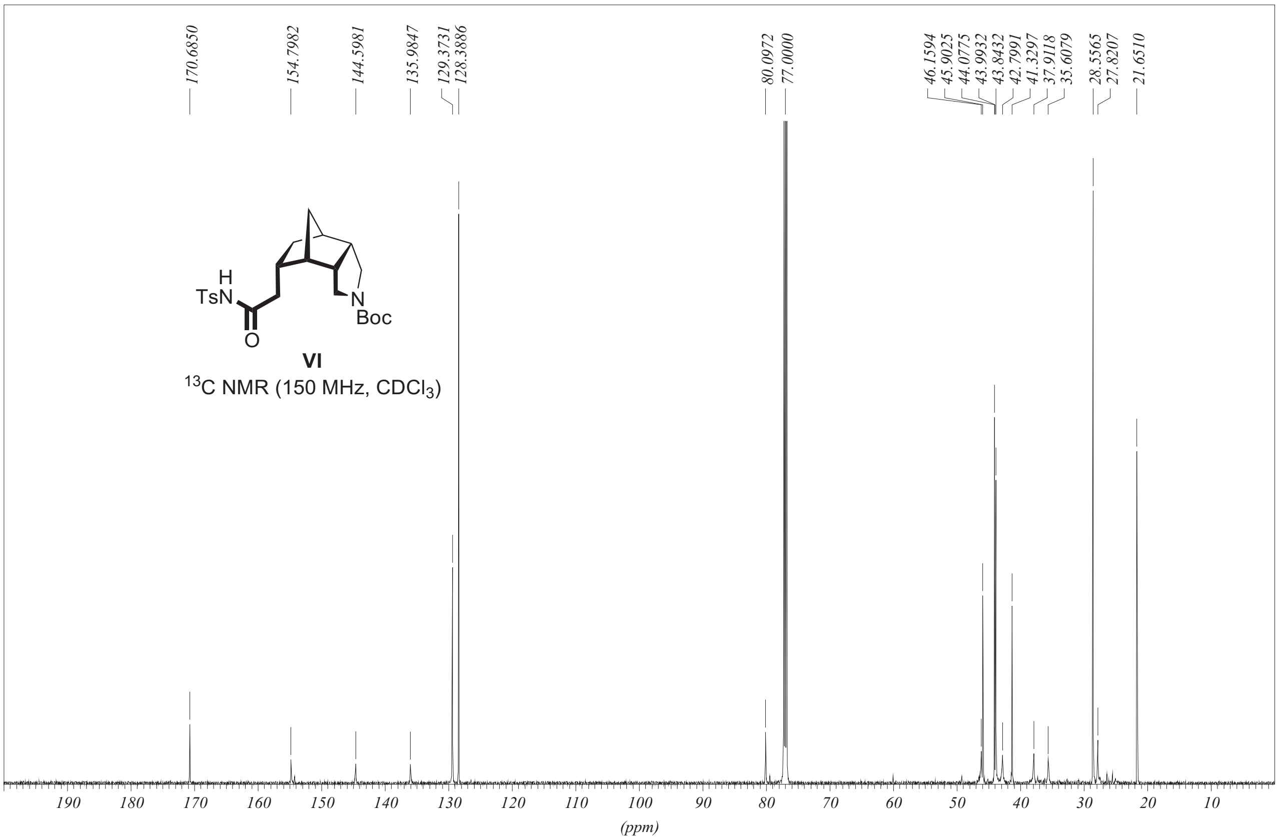
170.6850
154.7982
144.5981
135.9847
129.3731
128.3886
80.0972
77.0000
46.1594
45.9025
44.0775
43.9932
43.8432
42.7991
41.3297
37.9118
35.6079
28.5565
27.8207
21.6510
H
TsN
N
Boc
O
VI
^{13}C NMR (150 MHz, $CDCl_3$)
190
180
170
160
150
140
130
120
110
100
90
80
70
60
50
40
30
20
10
(ppm)

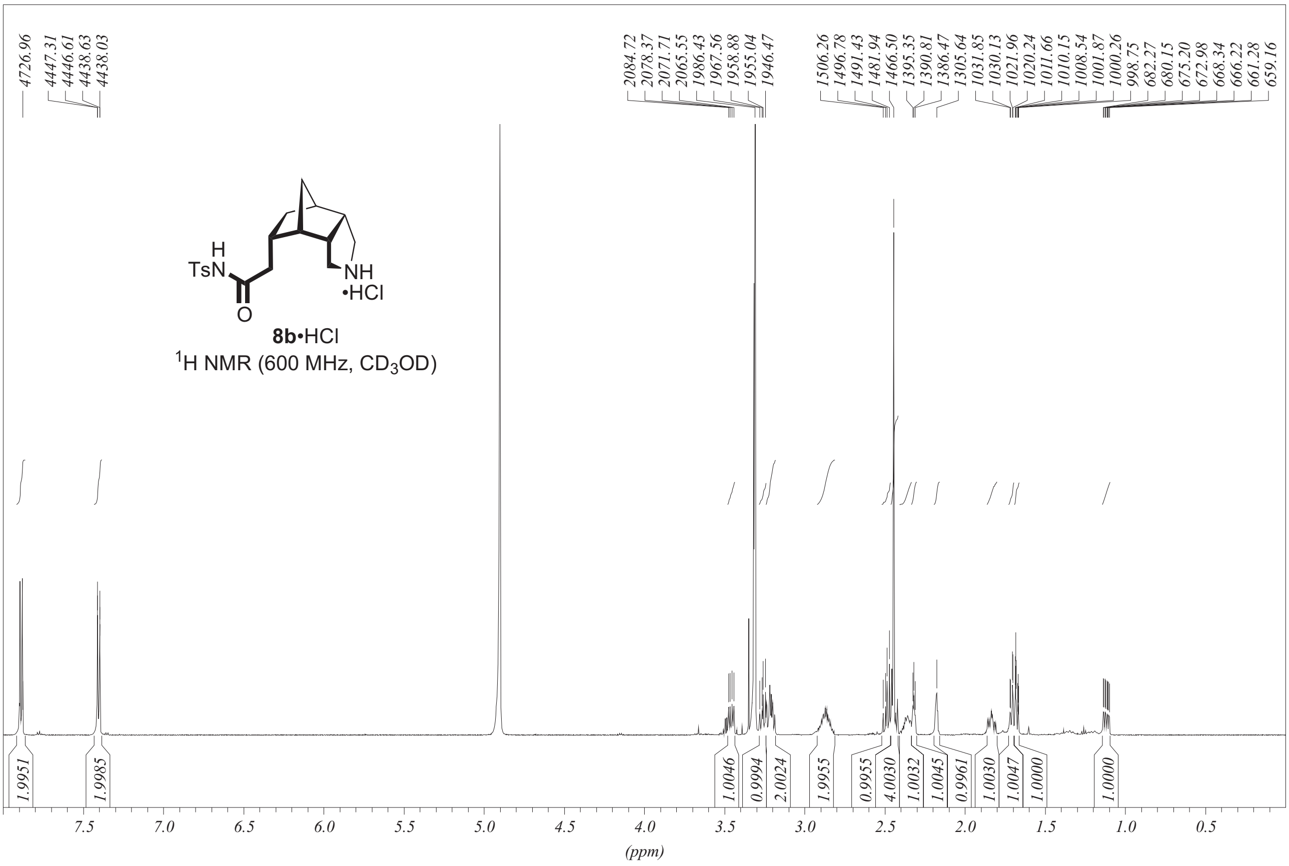
TsN
H
NH
•HCl
O
8b•HCl
1H NMR (600 MHz, CD3OD)
(ppm)

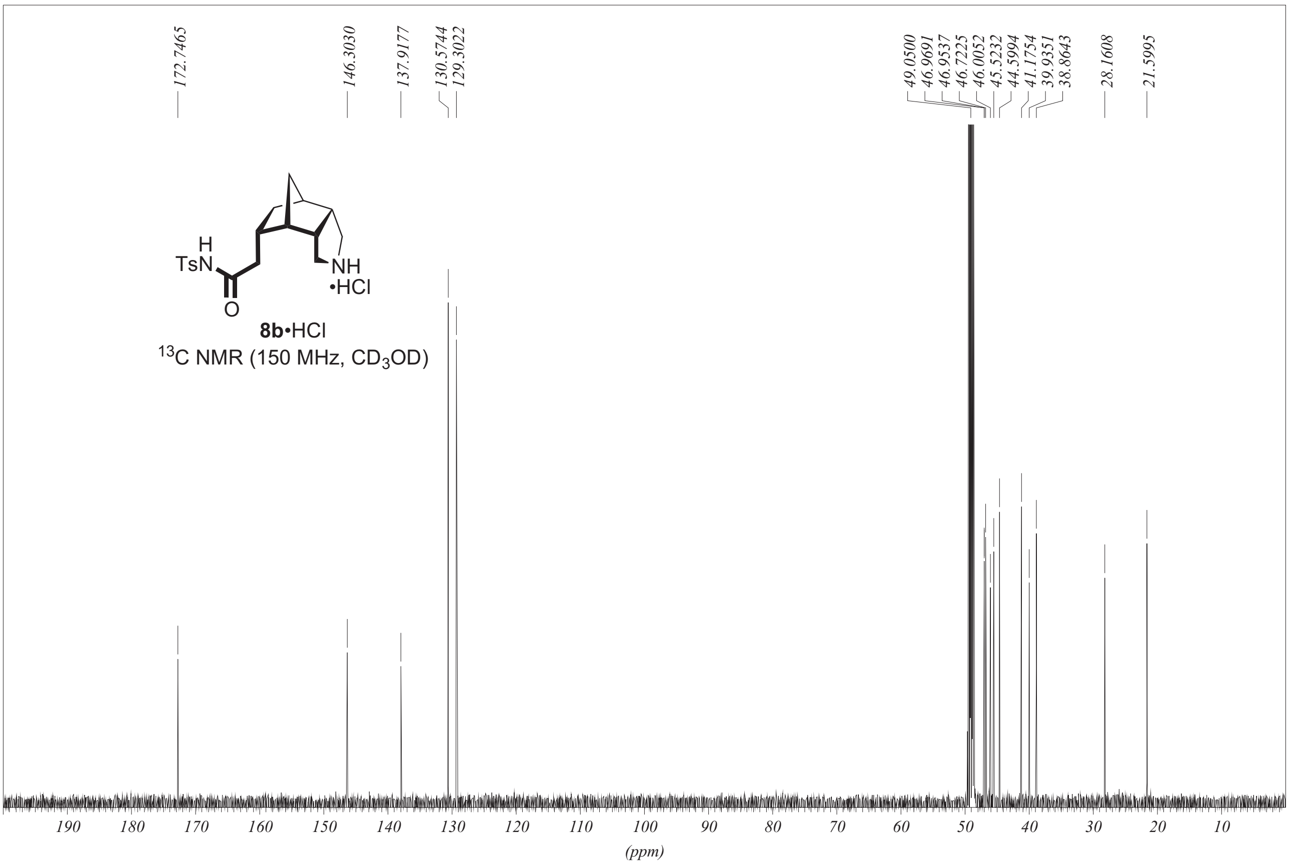
TsN
H
NH
•HCl
O
8b•HCl
13C NMR (150 MHz, CD3OD)
(ppm)
