# Supplementary material for: Enantioselective synthesis of tricyclic amino acid derivatives based on a rigid 4-azatricyclo[5.2.1.02,6]decane skeleton
Source: Beilstein J Org Chem. 2009 Dec 21;5:81. doi: 10.3762/bjoc.5.81 (PMC2839531; doi:10.3762/bjoc.5.81)
Supplement: File 3 — Crystallographic data of the compounds 8a•MeOH and 9. [file Beilstein_J_Org_Chem-05-81-s003.pdf]

Supporting Information File 3:

Crystallographic data of the compounds 8a•MeOH and 9

Enantioselective synthesis of tricyclic amino acid derivatives based on a rigid 4-azatricyclo[5.2.1.0^{2,6}]decane skeleton

Matthias Breuning*,[1], Tobias Häuser[1], Christian Mehler[1], Christian Däschlein[2], Carsten Strohmann[2], Andreas Oechsner[3] and Holger Braunschweig[3]

Address: [1]Institut für Organische Chemie, Universität Würzburg, Am Hubland, 97074 Würzburg, Germany, [2]Anorganische Chemie, Universität Dortmund, Otto-Hahn-Str. 6, 44227 Dortmund, Germany and [3]Institut für Anorganische Chemie, Universität Würzburg, Am Hubland, 97074 Würzburg, Germany

Email: Matthias Breuning* - breuning@chemie.uni-wuerzburg.de

* Corresponding author

Table of Contents

1. General Information S3

2. Crystallographic data of compound **9** S4

3. Crystallographic data of compound **8a**•MeOH S8

1. General Information

1.1 Crystal-Structure Determination of 9

Crystal structure determination of **9** was accomplished on a Bruker APEX diffractometer (D8 three-circle goniometer) (Bruker AXS); data collection, cell determination and refinement: Smart version 5.622 (Bruker AXS, 2001); integration: SaintPlus version 6.02 (Bruker AXS, 1999); empirical absorption correction: Sadabs version 2.01 (Bruker AXS, 1999). The structures were solved by applying direct and Fourier methods, using SHELXS-908 [1] and SHELXL-97 [2]. The non-hydrogen atoms were refined anisotropically. All of the H-atoms were placed in geometrically calculated positions and each was assigned a fixed isotropic displacement parameter based on a riding-model. CCDC 743050 contains the detailed crystallographic data for this publication. This data may be obtained free of charge from the Cambridge Crystallographic Data Center through www.ccdc.cam.ac.uk/data_request/cif.

1.2 Crystal structure determination of 8a•MeOH

The crystal data of **8a**•MeOH were collected at Bruker APEX diffractometer with CCD area detector and graphite monochromated $Mo_{K\alpha}$ radiation. The structure was solved using direct methods, refined with Shelx software package (G. Sheldrick, University of Göttingen 1997), and expanded using Fourier techniques. All non-hydrogen atoms were refined anisotropically. Hydrogen atoms were assigned idealized positions and were included in structure factors calculations.

Crystallographic data have been deposited with the Cambridge Crystallographic Data Center as supplementary publication no. CCDC 742656. These data can be obtained free of charge from The Cambridge Crystallographic Data Centre via www.ccdc.cam.ac.uk/data_request/cif.

1. G. M. Sheldrick, SHELXS-90, *A Program for the Solution of Crystal Structures*, Universität Göttingen **1990**.
2. G. M. Sheldrick, SHELXL-97, *A Program for Crystal Structure Refinement*, Universität Göttingen **1997**.

2. Crystallographic Data of compound 9

**Table 1:** Crystal Data and Structural Refinement Details for compound **9**.	
compound	**9**
empirical formula	$C_{14}H_{21}NO_3$
molecular mass [g·mol^{-1}]	251.32
temperature [K]	173(2)
wavelenght [Å]	0.71073 Å
crystal system	orthorhombic
space group (Nr.)	$P2_12_12_1$ (19)
a [Å]	10.407(2)
b [Å]	11.020(2)
c [Å]	11.661(3)
V [Å^3]	1337.3(5)
Z	4
calculated density ρ [g·cm^{-3}]	1.248
absorption coefficient μ [mm^{-1}]	0.087
F(000)	544
crystal size [mm^3]	0.40 x 0.40 x 0.30
theta range for data collection 2θ [°]	2.54 – 24.99
index ranges	$-9 \leq h \leq 12$
	$-11 \leq l \leq 13$
	$-13 \leq l \leq 12$
reflections collected	5399
independent reflections	2335 (R_{int} = 0.0370)
refinement method	Full-matrix least-squares on F^2
data / restraints / parameter	2335 / 0 / 166
goodness-of-fit on F^2	1.012
final R-values [$I > 2\sigma(I)$]	$R1$ = 0.0380,
	$wR2$ = 0.0782
R-values (all data)	$R1$ = 0.0462,
	$wR2$ = 0.0807
Absolute structure parameter	0.2(13)
largest diff. peak and hole [e·Å^{-3}]	0.176 and –0.168

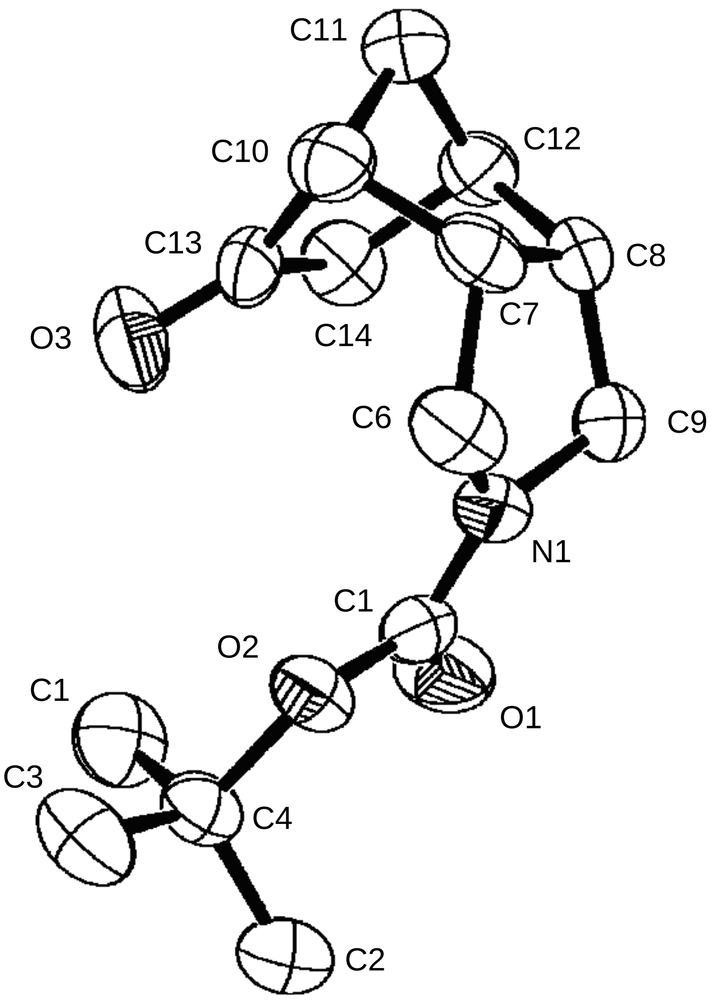

Figure 1: ORTEP plot of the asymmetric unit of compound **9** at 50 % probability level. H atoms were omitted for clarity.

Table 2: Atomic coordinates (x 10^4) and equivalent isotropic displacement parameters ($Å^2$ x 10^3) for compound **9**.

Atom	x	y	z	U(eq)
C(1)	7498(2)	–696(2)	2799(2)	68(1)
C(2)	8881(2)	–1384(2)	4444(2)	71(1)
C(3)	6511(2)	–1137(2)	4698(2)	67(1)
C(4)	7706(2)	–680(2)	4078(2)	47(1)
C(5)	8658(2)	1349(2)	4054(2)	48(1)
C(6)	7332(2)	2916(2)	5075(2)	54(1)
C(7)	7038(2)	4150(2)	4547(2)	49(1)
C(8)	8278(2)	4535(2)	3898(2)	48(1)
C(9)	9230(2)	3518(2)	4086(2)	64(1)
C(10)	6013(2)	4174(2)	3606(2)	51(1)
C(11)	6470(2)	5260(2)	2894(2)	56(1)
C(12)	7799(2)	4746(2)	2674(2)	47(1)
C(14)	6296(2)	3147(2)	2803(2)	49(1)
C(13)	7454(2)	3532(2)	2117(2)	55(1)
N(1)	8431(2)	2491(1)	4421(2)	51(1)
O(1)	9537(1)	1073(1)	3431(2)	73(1)
O(2)	7789(1)	575(1)	4491(1)	48(1)
O(3)	5733(2)	2194(1)	2724(1)	76(1)

Table 3: Anisotropic displacement parameters ($Å^2 \cdot 10^3$) compound **9**.

Atom	U^{11}	U^{22}	U^{33}	U^{23}	U^{13}	U^{12}
C(1)	85(2)	65(1)	54(1)	–15(1)	0(1)	2(1)
C(2)	75(2)	45(1)	93(2)	6(1)	–9(1)	7(1)
C(3)	78(1)	44(1)	80(2)	–6(1)	11(1)	–8(1)
C(4)	58(1)	36(1)	48(1)	–4(1)	1(1)	1(1)
C(5)	44(1)	46(1)	54(1)	10(1)	–8(1)	3(1)
C(6)	82(2)	46(1)	35(1)	1(1)	4(1)	–3(1)
C(7)	73(1)	35(1)	39(1)	–6(1)	6(1)	0(1)
C(8)	51(1)	36(1)	58(1)	0(1)	–14(1)	–8(1)
C(9)	47(1)	48(1)	97(2)	11(1)	–22(1)	–10(1)
C(10)	41(1)	51(1)	62(1)	3(1)	7(1)	4(1)
C(11)	59(1)	48(1)	62(1)	7(1)	–10(1)	5(1)
C(12)	50(1)	44(1)	47(1)	12(1)	4(1)	–5(1)
C(14)	48(1)	51(1)	50(1)	3(1)	–18(1)	–9(1)
C(13)	75(1)	57(1)	33(1)	1(1)	–1(1)	–4(1)
N(1)	48(1)	37(1)	67(1)	10(1)	–10(1)	–2(1)
O(1)	51(1)	55(1)	114(1)	9(1)	19(1)	10(1)
O(2)	64(1)	36(1)	44(1)	1(1)	2(1)	–4(1)
O(3)	76(1)	63(1)	90(1)	–8(1)	–17(1)	–26(1)

3. Crystallographic data of compound **8a**•MeOH

**Table 4:** Crystal Data and Structural Refinement Details for compound **8a**•MeOH.	
compound	**8a**•MeOH
empirical formula	$C_{11}H_{17}NO_2$•MeOH
molecular mass [g·mol^{-1}]	227.30
temperature [K]	168(2)
wavelength [Å]	0.71073
crystal system	monoclinic
space group (Nr.)	$P2_1$ (4)
a [Å]	7.0427(5)
b [Å]	8.5362(6)
c [Å]	9.9706(7)
β [°]	99.6710(10)
V [Å^3]	590.89(7)
Z	2
calculated density ρ [g·m^{-3}]	1.278
absorbtion coefficient μ [mm^{-1}]	0.091
F(000)	248
crystal size [mm^3]	0.35 x 0.12 x 0.12
theta range for data collection 2θ[°]	2.07 – 28.33
index ranges	$-9 \le h \le 9$, $-11 \le k \le 11$
	$-13 \le l \le 13$
reflections collected	14679
independent reflections	2942 (R_{int} = 0.0314)
completeness to theta = 26.00° [%]	100.0
absorption correction	semi-empirical from equivalents
minimum/maximum transmision	0.989 and 0.86316953
refinement method	Full-matrix least-squares on F^2
data / restrains / parameters	2942 / 1 / 148
goodness-of-fit on F^2	1.040
final *R*-values indices [I>2σ(I)]	R_1 = 0.0343, wR^2 = 0.0905
R-values (all data)	R_1 = 0.0350, wR^2 = 0.0910
Absolute structure parameter	0.0(8)
Largest diff. peak and hole [e·Å^{-3}]	0.275 and –0.154

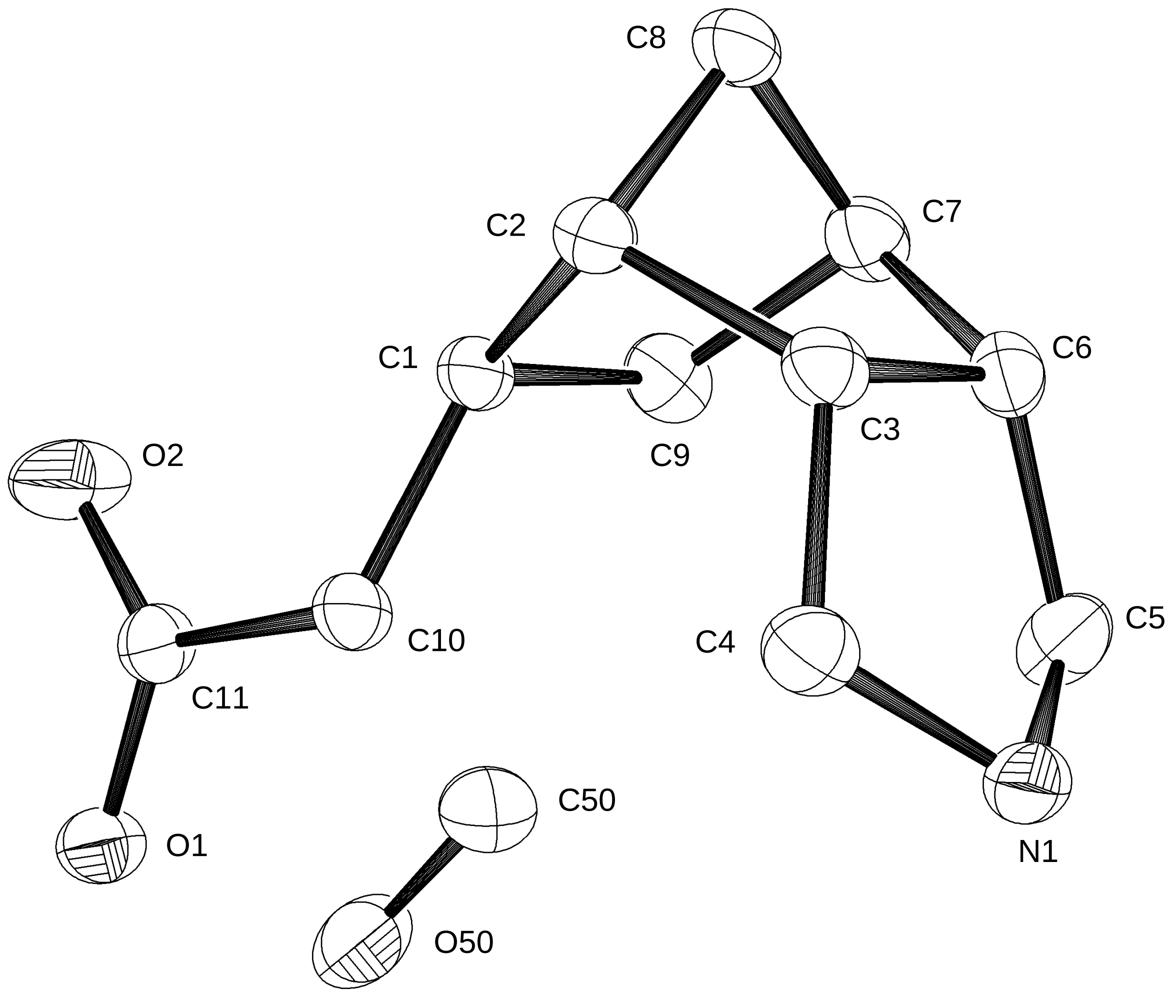

Figure 2: ORTEP plot of the asymmetric unit of compound **8a**•MeOH at 50 % probability level. H atoms were omitted for clarity.

Table 5: Atomic coordinates (x 10^4) and equivalent isotropic displacement parameters ($Å^2$ x 10^3) for **8a**•MeOH.

Atom	x	y	z	U(eq)
O(1)	6424(1)	-169(1)	396(1)	28(1)
N(1)	6273(1)	6632(1)	1112(1)	26(1)
C(1)	9489(2)	2804(1)	2385(1)	22(1)
O(2)	9153(2)	-453(1)	1833(1)	47(1)
C(2)	10701(2)	4293(1)	2251(1)	23(1)
C(3)	9562(2)	5817(1)	1878(1)	21(1)
C(4)	7982(2)	5931(1)	622(1)	25(1)
C(5)	6393(2)	6189(2)	2582(1)	31(1)
C(6)	8552(2)	6113(1)	3142(1)	24(1)
C(7)	9353(2)	4775(1)	4125(1)	26(1)
C(8)	11402(2)	4638(1)	3781(1)	27(1)
C(9)	8495(2)	3181(1)	3628(1)	27(1)
C(10)	8142(2)	2166(1)	1141(1)	23(1)
C(11)	7915(2)	372(1)	1144(1)	24(1)
O(50)	3022(2)	-1043(2)	2693(2)	56(1)
C(50)	4148(2)	235(2)	3231(2)	48(1)

Table 6: Anisotropic displacement parameters ($Å^2 \cdot 10^3$) compound **8a**•MeOH.

Atom	U^{11}	U^{22}	U^{33}	U^{23}	U^{13}	U^{12}
O(1)	26(1)	17(1)	38(1)	-1(1)	-5(1)	-1(1)
N(1)	25(1)	16(1)	32(1)	2(1)	-6(1)	1(1)
C(1)	23(1)	15(1)	26(1)	0(1)	-3(1)	1(1)
O(2)	39(1)	18(1)	73(1)	4(1)	-22(1)	1(1)
C(2)	20(1)	20(1)	26(1)	-2(1)	-1(1)	0(1)
C(3)	23(1)	18(1)	22(1)	1(1)	0(1)	-2(1)
C(4)	28(1)	21(1)	23(1)	3(1)	-1(1)	-1(1)
C(5)	27(1)	32(1)	33(1)	3(1)	4(1)	8(1)
C(6)	28(1)	19(1)	23(1)	-3(1)	1(1)	2(1)
C(7)	32(1)	23(1)	20(1)	1(1)	-1(1)	0(1)
C(8)	27(1)	23(1)	27(1)	-3(1)	-8(1)	1(1)
C(9)	36(1)	20(1)	24(1)	5(1)	1(1)	-3(1)
C(10)	24(1)	16(1)	26(1)	-1(1)	-2(1)	0(1)
C(11)	24(1)	15(1)	32(1)	-1(1)	1(1)	0(1)
O(50)	35(1)	39(1)	88(1)	-6(1)	-4(1)	5(1)
C(50)	34(1)	54(1)	53(1)	-11(1)	1(1)	0(1)
